# Enantioselective Preparation of Cyclopentene-Based Amino Acids with a Quaternary Carbon Center

**DOI:** 10.1021/acs.joc.4c01764

**Published:** 2024-10-29

**Authors:** Michael Franc, Pavel Měrka, Ivana Císařová, Jan Veselý

**Affiliations:** † Department of Organic Chemistry, Faculty of Science, Charles University, Hlavova 2030, 128 43 Praha 2, Czech Republic; ‡ Department of Inorganic Chemistry, Faculty of Science, Charles University, Hlavova 2030, 128 43 Praha 2, Czech Republic

## Abstract

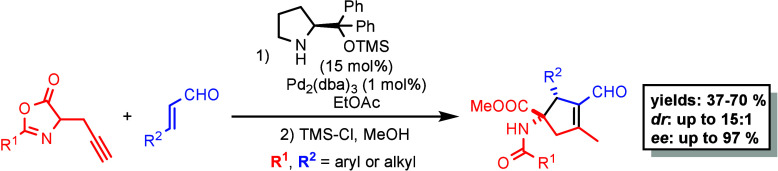

Azlactone is an important starting material for synthesizing amino acids containing a quaternary α-carbon. In this study, we have developed a sequential “one-pot” procedure involving an enantioselective spirocyclization reaction followed by acidic azlactone opening, which led to amino acid derivatives. The key step of this procedure is a spirocyclization between propargylated azlactones and enals by using a cooperative catalytic approach that combines chiral secondary amine and achiral Pd(0) complexes. The final acid opening of the azlactone motif allows isolation of the corresponding amino acid derivatives as major diastereoisomers in yields ranging from 37% to 70% with enantioselectivities of 85–97% ee. These synthesized amino acid derivatives hold great potential in the pharmaceutical and bioactive compound industries. Moreover, the final amino acid products with a cyclopentene moiety can be further derivatized, opening up even more possibilities for their application.

## Introduction

**Scheme 1:**
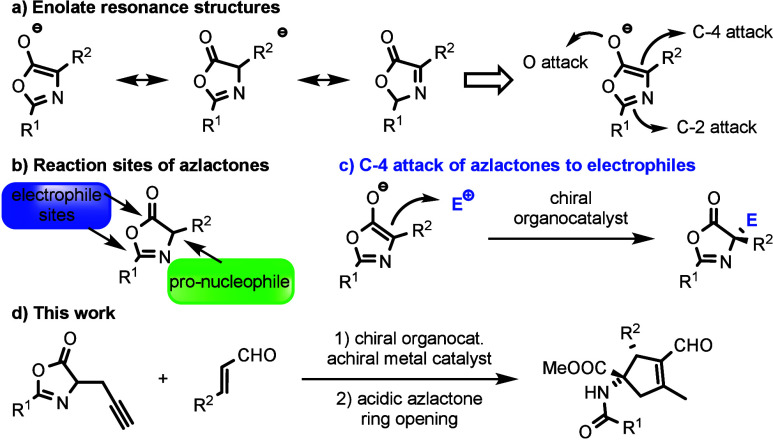
(a) Resonant Structures of Azlactone Enolate, (b) Reactivity of Azlactone, (c) Azlactone Reactivity in the C-4 Position, and (d) Our Work

α-Amino acids (α-AA) are part of proteins, peptides, enzymes, and many hormones. Their derivatives also serve as neurotransmitters or substances that influence cell growth. α-AA are associated with all living organisms; therefore, it is important to develop new ways to synthesize them.^1^ For such a goal, azlactones (also known as oxazolones or oxazol-5(4*H*)-ones) have been an attractive choice because their scaffold consists of “masked” amino acids, which can be used in the synthesis of natural or synthetic bioactive compounds.^2^ Structurally, azlactones or oxazolones are heterocyclic compounds containing a nitrogen atom in the β-position instead of the carbon, as in the well-known butenolide structures. The reactivity of azlactone is associated with the presence of acidic hydrogen (p*K*_a_ ≈ 9), which is caused by the aromatic character of the corresponding enol tautomers that can react with electrophiles (Scheme 1a and 1c). Based on the resonance structures, the electrophile can react with the α-carbonyl (C-4 attack) or aminal (C-2 attack) position. In these cases, at least one stereogenic center is generated. On the other hand, they also have two extra electrophilic sites where nucleophiles can be directed (Scheme 1b).^3−5^ These electrophilic sites are used in dynamic kinetic resolution reactions leading to chiral functionalized amino esters and amides.^6^ In the case of the pronucleophilic nature of the azlactone motif, the alkylation of oxazolones at their C-4 position is sought, leading to chiral α-substituted amino acids. Besides alkylation of the C-4 position using catalysis with transition metals,^7−14^ organocatalysis also allows efficient construction of quaternary carbon centers on an azlactone moiety. In 2008, the Jorgensen group (and Hayashi soon after) published the first azlactone addition to unsaturated aldehydes catalyzed by simple secondary amines.^15,16^ Successfully, other electrophiles were used in asymmetric Michael addition with oxazolones, such as nitroalkenes,^17^ enones,^18^ vinylsulfones,^19^ maleimides,^20^ itaconimides,^21^ and dehydroalanines.^22^ Furthermore, 1,6- and 1,8-conjugated additions of oxazolones to various electrophiles were also described.^23^ Another way to form a chiral quaternary carbon center on azlactone is through 1,2-addition reactions such as the aldol^24^ or Mannich^25^ reaction. Next, the aldol-type reaction of azlactones with vinyl ethers, catalyzed by chiral phosphoric acid, also provides enantiomerically enriched azlactone derivatives, which can be subsequently transformed into β-hydroxy-α-amino acid derivatives.^26^ Interestingly, the enantioselective α-sulfenylation of azlactone can also be performed.^27^ Last but not least, azlactones have been used in stereoselective [3 + 2] or [4 + 2] cycloaddition reactions due to their ability to form münchones (mesoionic oxazolone derivatives).^28,29^ Despite numerous scientific works, the azlactone motif is still an attractive tool for the preparation of unnatural amino acid derivatives, and its application in asymmetric synthesis is still relevant.

The cooperative catalysis approach, combining organocatalysis and metal catalysis, has recently emerged as a promising method for discovering new reactions or enhancing the efficiency of existing synthetic methods. This multicatalytic strategy involves two different catalysts in two separate catalytic cycles, simultaneously activating nucleophiles and electrophiles.^30^ The use of the azlactone motif in enantioselective transformations catalyzed by a combination of an organocatalyst and a metal catalyst is extremely rare. To the best of our knowledge, only two works have been published in this area. In 2019, Mukherjee et al. reported enantioselective decarboxylative [4 + 2]-annulation of ethynyl benzoxazinanones with azlactones under cooperative copper and bifunctional tertiary aminourea catalysis.^31^ In the same year, Shi and co-workers discovered chemodivergent and stereoselective reactions of vinyl benzoxazinones with azlactones cooperatively catalyzed by Bronsted acid and iridium catalyst.^32^

As previously mentioned, the azlactone motif is key to preparing chiral unnatural amino acids (UAAs), which can have various biological effects as single molecules or as a part of larger biomolecules, such as peptides.^33^ Therefore, it is important to continuously develop new methods for producing UAAs and discover new potentially bioactive UAAs. Based on these reasons and expanding our experience in synergistic catalysis,^34^ we designed a chiral secondary amine/palladium complex cocatalyzed cyclization reaction between propargylated azlactones and enals. This methodology enables the preparation of amino acid derivatives bearing a chiral quaternary carbon center (Scheme 1d).

## Results and Discussion

**Figure 1:**
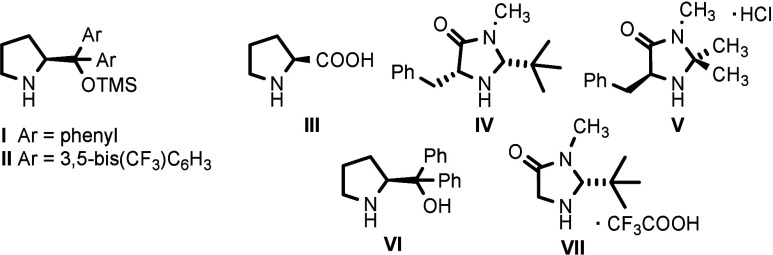
Tested chiral secondary amines.

We initially examined the cyclization reaction between propargylated azlactone **1a** and cinnamaldehyde **2a** catalyzed by a combination of organocatalyst **I** and Pd_2_(dba)_3_ in EtOAc (Table 1). The corresponding spiroazlactone **3a** was isolated in high yield (87%) with moderate diastereoselectivity (3:1 dr) and excellent enantiomeric excess (96/99% ee). Based on the promising initial result, we continued testing other chiral secondary amines **II**–**VII** (Figure 1) as organocatalysts in combination with Pd_2_(dba)_3_ (Table 1, entries 1–8). Regrettably, none of them outperformed the efficiency of organocatalyst **I**. Subsequently, we studied the effect of transition metal catalysts in the spirocyclization reaction. Several representative palladium catalysts, such as Pd_2_(dba)_3_, Pd(PPh_3_)_4_, Pd(OAc)_2_, PdCl_2_, and other metal complexes, were screened (for all tested conditions, please, see the ESI). Despite that, the tris(dibenzylideneacetone)dipalladium was a proper option for yield, diastereocontrol, and enantiocontrol of the reaction. The tested reaction showed high tolerance to various solvents (Table 1, entries 8–13), including nonpolar (toluene), chlorinated (CH_2_Cl_2_, CHCl_3_), ethereal (THF, TBME), and dipolar aprotic solvents (CH_3_CN, EtOAc). Unfortunately, the corresponding product **3a** was not obtained in a protic solvent due to the decomposition of the starting material (Table 1, entry 13). Next, the influence of catalyst loading on the course of the model reaction was tested. We reduced the amount of Pd_2_(dba)_3_ without decreasing the yield, diastereo-, and enantiocontrol of the reaction (Table 1, entry 14). In optimized conditions, the reaction between azlactone **1a** and cinnamaldehyde **2a** under the catalysis with a combination of **I** and Pd_2_(dba)_3_ provided the spiro compound **3a** in high yield (89%) with moderate diastereomeric ratio (3.1:1 dr) and excellent enantiopurity (98/99% ee).

**Table 1:**
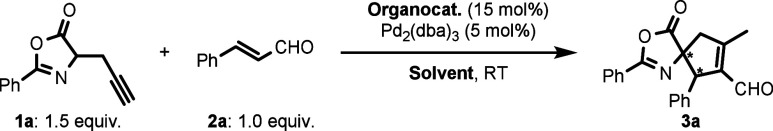
Condition Screening of Catalytic Reactions between Azlactone **1a** and Enal **2a**

Entry	Organocat.	Solvent	Time [days]	dr^a^	Yield [%]	ee [%]^b^
1	**I**	EtOAc	1	3:1	87	96/99
2	**II**	EtOAc	6	1.7:1	49	91/99
3	**III**	EtOAc	6	–	n.r.	-
4	**IV**	EtOAc	6	–	n.r.	-
5	**V**	EtOAc	6	1,6:1	25	78/62
6	**VI**	EtOAc	6	3.4:1	22	79/85
7	**VII**	EtOAc	6	1.8:1	traces.	n.d.
8	**I**	CH_3_CN	6	3:1	30	86/96
9	**I**	Acetone	2	3:1	76	97/98
10	**I**	Toluene	1	2.5:1	93	95/98
11	**I**	CH_2_Cl_2_	2	2.6:1	81	96/98
12	**I**	THF	4	2.5:1	68	93/94
13^c^	**I**	MeOH	1	–	n.r.	-
14^d^	**I**	EtOAc	1	3.1:1	89	98/99

a Determined by ^1^H NMR.

b Determined by chiral HPLC.

c Decomposition of starting material.

d 1 mol % of Pd_2_(dba)_3_.

**Scheme 2:**
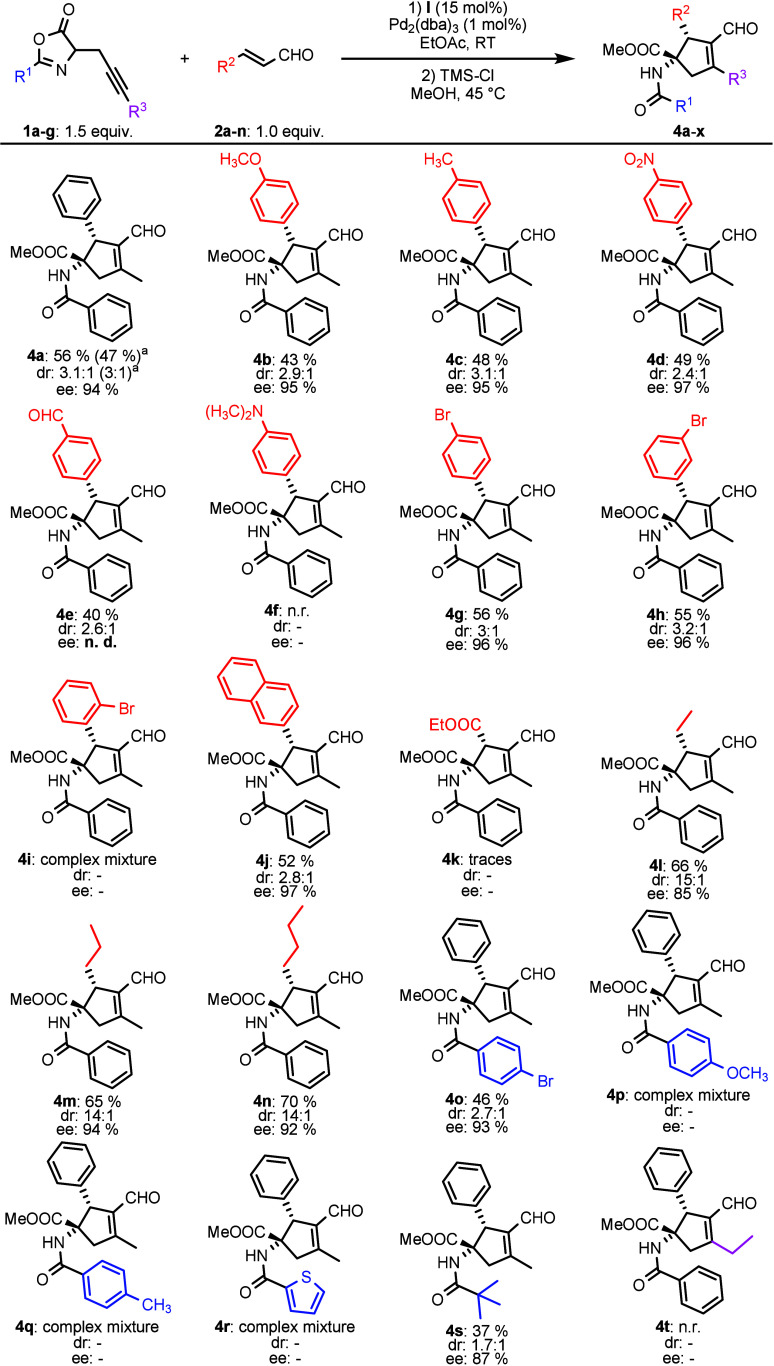
Substrate Scope of the Catalytic Reaction between Azlactones **1a**–**g** and Enals **2a**–**n** Scale up: 1.8 mmol of **1a** enal and 1.2 mmol of **2a**.

With the optimized reaction conditions in hand, we conducted the stereoselective spirocyclization reaction followed by an acidic opening of the azlactone moiety. The acidic opening was performed by *in situ* generated hydrochloric acid from trimethylsilyl chloride in the presence of methanol. This sequential one-pot procedure allowed us to obtain the cyclopentene derivative **4a** as a pure major diastereoisomer in good yield (56%) with an excellent enantiomeric excess (94% ee). Encouraged by this result, we continued to test various enals (Scheme 2). Initially, the cinnamic type enals were tested with **2a**–**i** in reaction with azlactone **1a**. Cinnamaldehyde **2b**–**g** with an electron-donating (EDG) and electron-withdrawing (EWG) group in the *para* position provided similar results, and the cyclopentene products **4b**–**g** were isolated as the major diastereoisomers in good yields (40–56%) with excellent enantiopurity (up to 97% ee). Except for cinnamaldehyde with a dimethylamino group **2f**, the formation of product **4f** in the reaction with azlactone **1a** was not observed. However, for example, the reaction of *para*-bromo cinnamaldehyde **2e** with azlactone **1a** provided cyclopentene **4g** in 56% yield with excellent enantiomeric purity (96% ee). Additionally, the *ortho* and *meta* substitution on the aromatic part of cinnamaldehyde was studied. In the case of *meta*-bromo cinnamaldehyde **2h**, the desired product **4h** was isolated as the major diastereoisomer in good yield (55%) and excellent enantiomeric excess (96% ee). On the other hand, the *ortho*-bromo-substituted product **4i** was not obtained, and the formation of the complex mixture was observed. An example of an extended aromatic system, enal (**2j**), afforded the major diastereoisomer **4j** in 52% yield with an enantiomeric excess of 97%.

Subsequently, we tested nonaromatic enals (**2k**–**n**) in a reaction with azlactone **1a**. In the case of enal with ester group **2k**, the traces of product **4k** were observed. On the other hand, the increase in diastereoselectivity of the spirocyclization reaction was observed when aliphatic aldehydes (**2l**–**n**) were used (up to 15:1 dr) but with a slight reduction of enantiocontrol (85–94% ee). For example, the reaction of heptenal **2n** with azlactone **1a** gave cyclopentene **4n** in 70% yield with an enantiopurity of 92%.

After that, we tested the reactivity of azlactones modified at position 2 with various *para*-substituted phenyl groups. Nevertheless, only the azlactone **1b** with *para*-bromo phenyl in position 2 in reaction with cinnamaldehyde **2a** provided the desired product **4o** in good yield (46%) with excellent enantiopurity (93% ee). Conversely, reactions of **2a** with azlactones bearing electron-rich groups such as *para*-methoxy- and methylphenyl (**1c** or **1d**) provided inseparable complex mixtures. Similarly, the cyclopentene **4r** was not isolated, and the complex mixture was obtained in the reaction between azlactone **1e** and cinnamaldehyde **2a**. On the other hand, azlactone **1f** modified with the *t*-butyl group was tolerated in reaction with cinnamaldehyde **2a**, and the corresponding product **4s** was separated as a major diastereoisomer in moderate yield (37%) with high enantiomeric excess (87% ee). Finally, azlactone **1g** with an internal triple bond was also tested in reaction with cinnamaldehyde **2a**, but no reaction was observed.

**Figure 2:**
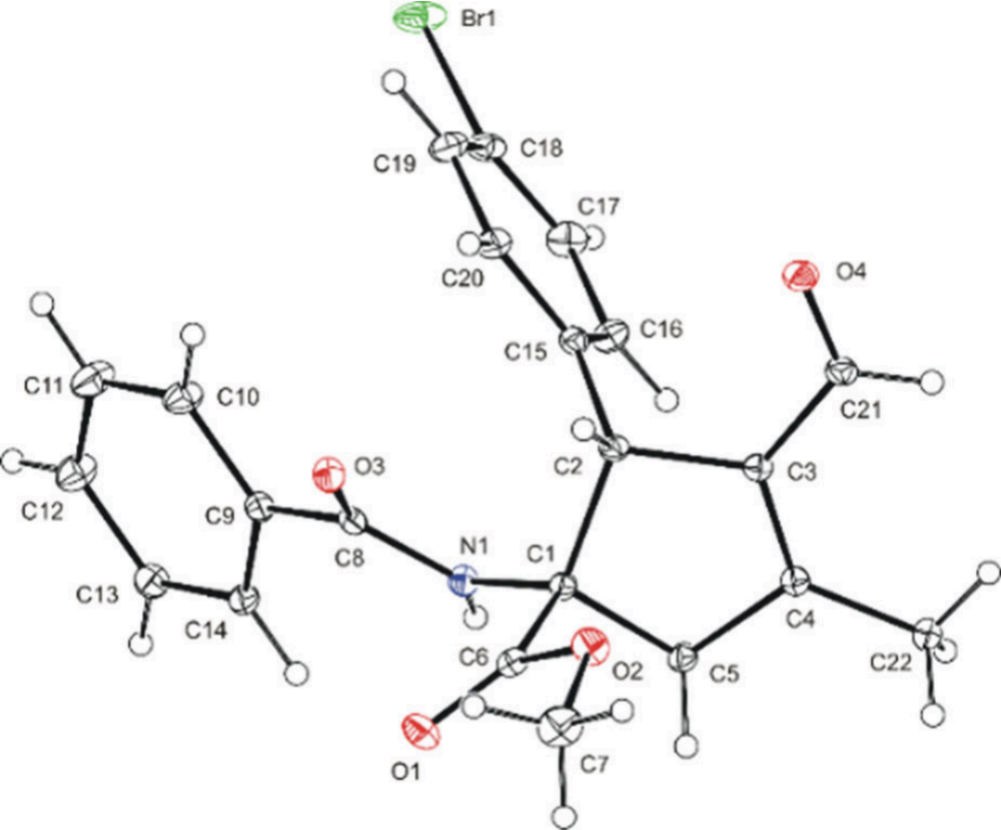
View of molecule of **4g** with atom numbering schema indicating *S* and *R* configuration on C1 and C2, respectively.

The absolute configuration of the main diastereomers of prepared cyclopentenes **4** was assigned based on the X-ray diffraction analysis of cyclopentene **4g**. The absolute configuration was determined as (1*S*, 2*R*) on the cyclopentene ring of compound **4g** (Figure 2).

**Scheme 3:**
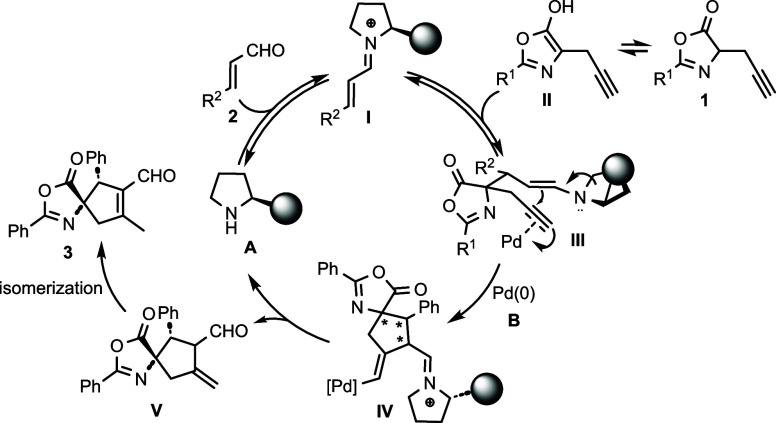
Proposed Mechanism

Based on the determined absolute configuration of **4g** and in agreement with our previous study,^34c^ a reaction mechanism for an asymmetric cyclization of enals **2** with azlactone **1** can be proposed (Scheme 3). The catalytic cycle starts with the formation of iminium **I** via the condensation of enal **2** with the chiral secondary amine **A**. Then, iminium **I** undergoes conjugate addition with enol **II**, generated from enolizable azlactone **1**, affording enamine **III**. Next, the palladium catalyst is coordinated to a triple bond, resulting in a Conia–ene reaction that leads to spirocyclic compound **IV**. After both catalysts are released, the exocyclic double bond is then isomerized to the final spiro azlactone **3**.

**Scheme 4:**
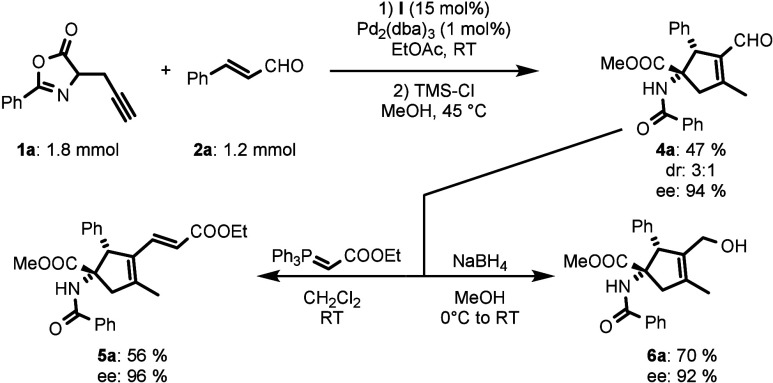
Molar Scale Reaction between **1a** and **2a** and Subsequent Transformations of **4a**

Subsequently, the synthetic utility of the developed methodology was demonstrated by a molar-scale reaction between azlactone **1a** and cinnamaldehyde **2a** (Scheme 4). The scale-up condition provided cyclopentene product **4a** in good yield (47%) without a significant decrease of enantiocontrol of the process (94% ee). To increase the molecular complexity of the obtained compounds **4**, selected follow-up transformations of compound **4a** were performed (Scheme 3). For example, aldehyde **4a** was converted to ester **5a** by Wittig olefination in good yield and without loss of enantiomeric purity. Similarly, aldehyde **4a** was reduced to alcohol **6a** in high yield with retained enantiomeric purity.

## Conclusion

In summary, we have developed the asymmetric synthesis of amino acid derivatives containing chiral quaternary carbon under cooperative catalysis of Pd_2_(dba)_3_ and simple chiral secondary amine followed by the opening azlactone motif. Corresponding products were separated as single diastereoisomers in good yields (up to 70%) and with excellent enantiomeric purities (up to 97 ee).

## Experimental Section

### General Information

Chemicals and solvents were either purchased (puriss p.A.) from commercial suppliers or purified by standard techniques. For thin-layer chromatography (TLC), silica gel plates Merck 60 F_254_ were used, and compounds were visualized by irradiation with UV light and/or by treatment with a solution of phosphomolybdenic acid (25 g), Ce(SO_4_)_2_·H_2_O (10 g), conc. H_2_SO_4_ (60 mL), and H_2_O (940 mL) followed by heating. Column chromatography was performed using silica gel Merck 60 (particle size 0.040–0.063 mm). ^1^H NMR, ^13^C NMR, and 2D NMR were recorded with a Bruker DPX400 NMR. Chemical shifts (δ) are reported in ppm relative to residual solvent signals (CHCl_3_, 7.26 ppm for ^1^H NMR; CDCl_3_, 77.1 ppm for ^13^C NMR). High-resolution mass spectra were recorded on an LCQ Fleet spectrometer using a Bruker Compact QTOF-MS controlled by the Compass 1.9 Control software to measure the ESI high-resolution mass spectra. The monoisotopic mass values were calculated using Data analysis software v 4.4. The analysis was conducted in the positive ion mode at a scan range from *m*/*z* 50 to 1000, and nitrogen was used as a nebulizer gas at a pressure of 4 psi and flow of 3 L/min for the dry gas. The capillary voltage and temperature were set at 4500 V and 220 °C, respectively. Optical rotations were performed on an AU-Tomatica polarimeter, Autopol III. IR DRIFT spectra were recorded with a Nicolet AVATAR 370 FT-IR in cm^–1^. The HPLC analysis was performed on a LC20AD Shimadzu liquid chromatograph with an SPD-M20A diode array detector with columns of Daicel Chiralpak.

Starting azlactones **1a**–**g** were prepared according to a known procedure.^35^

#### 2-Phenyl-4-(prop-2-yn-1-yl)oxazol-5(4*H*)-one (**1a**)

^1^H NMR (400 MHz, CDCl_3_) δ 8.06–7.97 (m, 2H), 7.62–7.54 (m, 1H), 7.53–7.44 (m, 2H), 4.54 (t, *J* = 5.3 Hz, 1H), 2.91 (qdd, *J*_1_ = 16.9, *J*_2_ = 5.3, *J*_3_ = 2.6 Hz, 2H), 2.02 (t, *J* = 2.7 1H). ^13^C{^1^H} NMR (101 MHz, CDCl_3_) δ: 176.6, 162.9, 133.2, 132.0, 128.9 (2C), 128.2 (2C), 77.5, 71.9, 64.2, 21.7. IR (ATR): ν = 3331, 3209, 1817, 1631, 1450 cm^–1^. HRMS (ESI) *m*/*z* calcd for C_12_H_9_NO_2_ [M + H]^+^ 200.07061, found 200.07036.

#### 2-(4-Bromophenyl)-4-(prop-2-yn-1-yl)oxazol-5(4*H*)-one (**1b**)

^1^H NMR (400 MHz, CDCl_3_) δ 7.89 (d, *J* = 8.6 Hz, 2H), 7.64 (d, *J* = 8.6 Hz, 2H), 4.53 (t, *J* = 5.3 Hz, 1H), 2.92 (qdd, *J*_1_ = 16.9, *J*_2_ = 5.3, *J*_3_ = 2.6 Hz, 2H), 2.02 (t, *J* = 2.6 Hz, 1H). ^13^C{^1^H} NMR (101 MHz, CDCl_3_) δ: 176.3, 162.3, 132.4 (2C), 129.7 (2C), 128.2, 124.6, 77.4, 72.0, 64.3, 21.7. IR (ATR): ν = 3275, 1817, 1651, 1485, 1319 cm^–1^. HRMS (ESI-TOF) *m*/*z* calcd for C_12_H_9_BrNO_2_ [M + H]^+^ 277.9811, found 277.9812.

#### 2-(4-Methoxyphenyl)-4-(prop-2-yn-1-yl)oxazol-5(4*H*)-one (**1c**)

^1^H NMR (400 MHz, CDCl_3_) δ 8.00–7.93 (m, 2H), 7.01–6.93 (m, 2H), 4.51 (t, *J* = 5.3 Hz, 1H), 3.87 (s, 3H), 2.89 (qdd, *J*_1_ = 16.9, *J*_2_ = 5.3, *J*_3_ = 2.6 Hz, 2H), 2.02 (t, *J* = 2.6 Hz, 1H). ^13^C{^1^H} NMR (101 MHz, CDCl_3_) δ: 176.8, 163.6, 162.8, 130.2 (2C), 117.8, 114.4 (2C), 77.6, 71.8, 64.1, 55.6, 21.8. IR (ATR): ν = 3271, 1805, 1647, 1512, 1261 cm^–1^. HRMS (ESI-TOF) *m*/*z* calcd for C_13_H_12_NO_3_ [M + H]^+^ 230.0812, found 230.0813.

#### 4-(Prop-2-yn-1-yl)-2-(p-tolyl)oxazol-5(4*H*)-one (**1d**)

^1^H NMR (400 MHz, CDCl_3_) δ 7.91 (d, *J* = 8.3 Hz, 2H), 7.29 (d, *J* = 8.1 Hz, 2H), 4.53 (t, *J* = 5.3 Hz, 1H), 2.90 (qdd, *J* = 17.0, 5.3, 2.6 Hz, 2H), 2.42 (s, 3H), 2.01 (t, *J* = 2.6 Hz, 1H). ^13^C{^1^H} NMR (101 MHz, CDCl_3_) δ: 176.8, 163.0, 144.0, 129.7, 128.2, 127.9, 77.6, 71.8, 64.2, 55.0, 21.8. IR (ATR): ν = 3263, 1811, 1647, 1508, 1319 cm^–1^. HRMS (ESI-TOF) *m*/*z* calcd for C_13_H_12_NO_2_ [M + H]^+^ 214.0863, found 214.0862.

#### 4-(Prop-2-yn-1-yl)-2-(thiophen-2-yl)oxazol-5(4*H*)-one (**1e**)

^1^H NMR (400 MHz, CDCl_3_) δ 7.75 (dd, *J*_1_ = 3.8, *J*_2_ = 1.3 Hz, 1H), 7.62 (dd, *J*_1_ = 5.0, *J*_2_ = 1.2 Hz, 1H), 7.16 (dd, *J*_1_ = 5.0, *J*_2_ = 3.8 Hz, 1H), 4.53 (t, *J* = 5.2 Hz, 1H), 2.98–2.83 (m, 2H), 2.03 (t, *J* = 2.6 Hz, 1H). ^13^C{^1^H} NMR (101 MHz, CDCl_3_) δ: 176.1, 158.7, 132.6, 132.4 (2C), 128.3, 77.4, 72.0, 64.1, 21.7. IR (ATR): ν = 3271, 1809, 1645, 1537, 1311 cm^–1^. HRMS (ESI-TOF) *m*/*z* calcd for C_10_H_8_NO_2_S [M + H]^+^ 206.0607, found 206.0611.

#### 2-(*tert*-Butyl)-4-(prop-2-yn-1-yl)oxazol-5(4*H*)-one (**1f**)

^1^H NMR (400 MHz, CDCl_3_) δ 4.29 (t, *J* = 4.8 Hz, 1H), 2.90–2.73 (m, 2H), 1.99 (t, *J* = 2.6 Hz, 1H), 1.29 (s, 9H). ^13^C{^1^H} NMR (101 MHz, CDCl_3_) δ: 177.4, 173.6, 77.3, 71.6, 63.3, 34.4, 26.9 (3C), 21.4. IR (ATR): ν = 3219, 1824, 1658, 1479, 1281 cm^–1^. HRMS (ESI-TOF) *m*/*z* calcd for C_10_H_14_NO_2_ [M + H]^+^ 180.1019, found 180.1018.

#### 4-(But-2-yn-1-yl)-2-phenyloxazol-5(4*H*)-one (**1g**)

^1^H NMR (400 MHz, CDCl_3_) δ 8.06–8.00 (m, 2H), 7.62–7.55 (m, 1H), 7.49 (t, *J* = 7.7 Hz, 2H), 4.50 (t, *J* = 5.2 Hz, 1H), 2.96–2.74 (m, 2H), 1.69 (t, *J* = 2.5 Hz, 3H). ^13^C{^1^H} NMR (101 MHz, CDCl_3_) δ: 177.2, 162.7, 133.0, 128.9 (2C), 128.2 (2C), 125.9, 79.5, 72.2, 64.9, 22.2, 3.6. IR (ATR): ν = 3219, 1824, 1658, 1479, 1281 cm^–1^. HRMS (ESI-TOF) *m*/*z* calcd for C_13_H_12_NO_2_ [M + H]^+^ 214.0863, found 214.0862.

### General Procedure for the Synthesis of Compounds **4**

The corresponding α,β-unsaturated aldehyde **2** (0.12 mmol, 1 equiv), azlactone derivative **1** (0.18 mmol, 1.5 equiv), and Pd_2_(dba)_3_ (0.0012 mmol, 0.01 equiv) were added to a solution of organic catalyst 2-(diphenyl((trimethylsilyl)oxy)methyl)pyrrolidine **I** (0.018 mmol, 0.15 equiv) in EtOAc (0.5 mL). The reaction mixture was stirred at room temperature and checked by ^1^H NMR. After full conversion, the crude mixture was evaporated. Crude product was dissolved in MeOH (1 mL), and trimethylsilyl chloride (0.36 mol, 3 equiv) was added to this solution. The reaction mixture was stirred at 45 °C (oil bath) for 2 h. The solvent was evaporated, and product **4** was isolated after silica separation in Hex/EtOAc (3:2).

### Scale up Synthesis of Compound **4a**

The corresponding α,β-unsaturated aldehyde **2a** (1.2 mmol, 1 equiv), azlactone derivative **1a** (1.8 mmol, 1.5 equiv), and Pd_2_(dba)_3_ (0.012 mmol, 0.01 equiv) were added to a solution of organic catalyst 2-(diphenyl((trimethylsilyl)oxy)methyl)pyrrolidine **I** (0.18 mmol, 0.15 equiv) in EtOAc (5 mL). The reaction mixture was stirred at room temperature and checked by ^1^H NMR. After full conversion the crude mixture was evaporated. Crude product was dissolved in MeOH (1 mL), and trimethylsilyl chloride (3.6 mol, 3 equiv) was added to this solution. The reaction mixture was stirred at 45 °C (oil bath) for 2 h. The solvent was evaporated, and product **4a** was isolated after silica separation in Hex/EtOAc (3:2). Compound **4a** was obtained as a yellowish foam in 47% yield (205 mg) with ee of 94%.

#### Methyl (1*S*,2*R*)-1-Benzamido-3-formyl-4-methyl-2-phenylcyclopent-3-ene-1-carboxylate (**4a**)

The title compound was synthesized according to the general procedure and purified by column chromatography (hexane/EtOAc 3:2): yellowish foam, yield 56% (24 mg), ee 94%. The ee was determined by HPLC analysis using a Chiralpak IA column (80/20 heptane/i-PrOH, flow rate 1.0 mL/min; λ = 190 nm, 25 °C), *t*_minor_ = 14.3 min; *t*_major_ = 18.0 min. ^1^H NMR (400 MHz, CDCl_3_) δ 9.99 (s, 1H), 7.49–7.37 (m, 5H), 7.31–7.20 (m, 5H), 6.06 (s, 1H), 4.52 (s, 1H), 3.97 (d, *J* = 19.2 Hz, 1H), 3.77 (s, 3H), 2.92 (dt, *J*_1_ = 19.3, *J*_2_ = 1.6 Hz, 1H), 2.36 (d, *J* = 1.3 Hz, 3H). ^13^C{^1^H} NMR (101 MHz, CDCl_3_) δ: 186.7, 173.7, 166.6, 162.5, 136.2, 136.0, 133.2, 131.9, 129.6 (2C), 128.8 (2C), 128.6 (2C), 127.3, 126.8 (2C), 64.0, 57.9, 53.3, 49.9, 14.6. IR (ATR): ν = 2951, 1736, 1639, 1525, 1205 cm^–1^. [α]^25^_D_ = +54.1° (0.85, CHCl_3_); HRMS (ESI-TOF) *m*/*z*: [M + Na]^+^ calcd for C_22_H_21_NO_4_Na 386.1363; found 386.1365.

#### Methyl (1*S*,2*R*)-1-Benzamido-3-formyl-2-(4-methoxyphenyl)-4-methylcyclopent-3-ene-1-carboxylate (**4b**)

The title compound was synthesized according to the general procedure and purified by column chromatography (hexane/EtOAc 3:2): yellowish foam, yield 43% (20 mg), ee 95%. The ee was determined by HPLC analysis using a Chiralpak IA column (70:30 heptane/i-PrOH, flow rate 1.0 mL/min; λ = 190 nm, 25 °C), *t*_minor_ = 9.7 min; *t*_major_ = 14.2 min. ^1^H NMR (400 MHz, CDCl_3_) δ 9.97 (s, 1H), 7.47–7.27 (m, 5H), 7.13 (d, *J* = 8.1 Hz, 2H), 6.99–6.93 (m, 2H), 6.13 (s, 1H), 4.47 (s, 1H), 3.95 (d, *J* = 20.3 Hz, 1H), 3.83 (s, 3H), 3.76 (s, 3H), 2.90 (dt, *J*_1_ = 19.3, *J*_2_ = 1.6 Hz, 1H), 2.34 (q, *J* = 1.3 Hz, 3H). ^13^C{^1^H} NMR (101 MHz, CDCl_3_) δ: 186.8, 173.8, 166.6, 162.3, 159.9, 136.4, 133.3, 131.9, 130.0 (2C), 128.7 (2C), 127.7, 126.8 (2C), 115.0 (2C), 64.0, 57.2, 55.5, 53.2, 49.8, 14.6 ppm. IR (ATR): ν = 2951, 1734, 1631, 1510, 1205 cm^–1^; [α]^25^_D_ = +41.7° (0.60, CHCl_3_); HRMS (ESI-TOF) *m*/*z*: [M + Na]^+^ calcd for C_23_H_23_NO_5_Na 416.1468; found 416.1467.

#### Methyl (1*S*,2*R*)-1-Benzamido-3-formyl-4-methyl-2-(*p*-tolyl)cyclopent-3-ene-1-carboxylate (**4c**)

The title compound was synthesized according to the general procedure and purified by column chromatography (hexane/EtOAc 3:2): yellowish foam, yield 48% (22 mg), ee 95%. The ee was determined by HPLC analysis using a Chiralpak IA column (80:20 heptane/i-PrOH, flow rate 1.0 mL/min; λ = 190 nm, 25 °C), *t*_minor_ = 12.0 min; *t*_major_ = 15.3 min. ^1^H NMR (400 MHz, CDCl_3_) δ 9.98 (s, 1H), 7.46–7.39 (m, 1H), 7.33–7.22 (m, 6H), 7.09 (d, *J* = 7.5 Hz, 2H), 6.11 (s, 1H), 4.48 (t, *J* = 1.7 Hz, 1H), 3.95 (d, *J* = 19.2 Hz, 1H), 3.76 (s, 3H), 2.90 (dt, *J*_1_ = 19.3, *J*_2_ = 1.6 Hz, 1H), 2.38 (s, 3H), 2.35 (q, *J* = 1.3 Hz, 3H). ^13^C{^1^H} NMR (101 MHz, CDCl_3_) δ: 186.8, 173.8, 166.6, 162.4, 138.6, 136.2, 133.3, 132.7, 131.9, 130.2 (2C), 128.7 (2C), 128.6 (2C), 126.8 (2C), 63.9, 57.6, 53.2, 49.9, 21.3, 14.5. IR (ATR): ν = 2951, 1736, 1639, 1514, 1205 cm^–1^; [α]^25^_D_ = +37.2° (0.90, CHCl_3_); HRMS (ESI-TOF) *m*/*z*: [M + Na]^+^ calcd for C_23_H_23_NO_4_Na 400.1519; found 400.1515.

#### Methyl (1*S*,2*R*)-1-Benzamido-3-formyl-4-methyl-2-(4-nitrophenyl)cyclopent-3-ene-1-carboxylate (**4d**)

The title compound was synthesized according to the general procedure and purified by column chromatography (hexane/EtOAc 3:2): yellowish foam, yield 49% (24 mg), ee 97%. The ee was determined by HPLC analysis using a Chiralpak IA column (70:30 heptane/i-PrOH, flow rate 1.0 mL/min; λ = 190 nm, 25 °C), *t*_minor_ = 11.7 min; *t*_major_ = 21.4 min. ^1^H NMR (400 MHz, CDCl_3_) δ 9.97 (s, 1H), 8.20–8.12 (m, 2H), 7.48–7.25 (m, 7H), 6.23–6.11 (m, 1H), 4.82 (s, 1H), 3.81–3.79 (m, 3H), 3.76–3.65 (m, 1H), 3.14 (d, *J* = 21.2 Hz, 1H), 2.36 (q, *J* = 1.5 Hz, 3H). ^13^C{^1^H} NMR (101 MHz, CDCl_3_) δ: 186.4, 173.3, 167.3, 161.6, 147.6, 144.4, 136.9, 133.1, 132.2, 130.0 (2C), 128.8 (2C), 126.6 (2C), 124.0 (2C), 65.0, 56.9, 53.5, 49.8, 14.6. IR (ATR): ν = 2951, 1738, 1655, 1514, 1344, 1205 cm^–1^; [α]^25^_D_ = −56.3° (1.11, CHCl_3_); HRMS (ESI-TOF) *m*/*z*: [M + Na]^+^ calcd for C_22_H_20_N_2_O_6_Na 431.1214, found 431.1213.

#### Methyl (1*S*,2*R*)-1-Benzamido-3-formyl-2-(4-formylphenyl)-4-methylcyclopent-3-ene-1-carboxylate (**4e**)

The title compound was synthesized according to the general procedure and purified by column chromatography (hexane/EtOAc 1:1): yellowish foam, yield 40% (19 mg), ee n.d. The ee was not determined due to not finding suitable conditions using HPLC. ^1^H NMR (400 MHz, CDCl_3_) δ 10.02 (s, 1H), 10.00 (s, 1H), 7.94–7.88 (m, 2H), 7.59–7.21 (m, 7H), 6.05 (s, 1H), 4.69 (s, 1H), 3.88 (d, *J* = 19.4 Hz, 1H), 3.81 (s, 3H), 3.06 (d, *J* = 19.4 Hz, 1H), 2.39 (s, 3H). ^13^C{^1^H} NMR (101 MHz, CDCl_3_) δ: 191.6, 186.5, 173.4, 167.0, 162.3, 143.4, 136.5, 136.3, 133.2, 132.1, 130.5, 129.7, 128.8, 128.7 (2C), 127.5, 126.7 (2C), 64.6, 57.6, 53. 5, 50. 0, 14.6. IR (ATR): ν = 2951, 1736, 1655, 1525, 1435, 1209 cm^–1^; [α]^25^_D_ = −28.6° (0.56, CHCl_3_); HRMS (ESI-TOF) *m*/*z*: [M + H]^+^ calcd for C_23_H_22_NO_5_ 392.1493; found 392.1492.

#### Methyl (1*S*,2*R*)-1-Benzamido-2-(4-bromophenyl)-3-formyl-4-methylcyclopent-3-ene-1-carboxylate (**4g**)

The title compound was synthesized according to the general procedure and purified by column chromatography (hexane/EtOAc 3:2): yellowish foam, yield 56% (30 mg), ee 96%. The ee was determined by HPLC analysis using a Chiralpak IA column (70:30 heptane/i-PrOH, flow rate 1.0 mL/min; λ = 190 nm, 25 °C), *t*_minor_ = 9.2 min; *t*_major_ = 14.5 min. ^1^H NMR (400 MHz, CDCl_3_) δ 9.96 (s, 1H), 7.53 (d, *J* = 8.6 Hz, 2H), 7.48–7.39 (m, 1H), 7.35–7.26 (m, 4H), 7.08 (d, *J* = 7.9 Hz, 2H), 6.04 (s, 1H), 4.52 (t, *J* = 1.7 Hz, 1H), 3.88 (d, *J* = 19.4 Hz, 1H), 3.77 (s, 3H), 2.96 (dt, *J*_1_ = 19.4, *J*_2_ = 1.5 Hz, 1H), 2.34 (q, *J* = 1.3 Hz, 3H). ^13^C{^1^H} NMR (101 MHz, CDCl_3_) δ: 186.5, 173.5, 166.9, 162.4, 136.3, 135.2, 133.2, 132.5 (2C), 132.1, 130.5 (2C), 128.8 (2C), 126.7 (2C), 122.6, 64.1, 57.2, 53.3, 49.8, 14.6. IR (ATR): ν = 2951, 1736, 1637, 1527, 1487, 1203 cm^–1^; [α]^25^_D_ = +6.1° (1.23, CHCl_3_); HRMS (ESI-TOF) *m*/*z*: [M + Na]^+^ calcd for C_22_H_20_BrNO_4_Na 464.0468; found 464.0471.

#### Methyl (1*S*,2*R*)-1-Benzamido-2-(3-bromophenyl)-3-formyl-4-methylcyclopent-3-ene-1-carboxylate (**4h**)

The title compound was synthesized according to the general procedure and purified by column chromatography (hexane/EtOAc 3:2): yellowish foam, yield 55% (29 mg), ee 96%. The ee was determined by HPLC analysis using a Chiralpak IA column (70:30 heptane/i-PrOH, flow rate 1.0 mL/min; λ = 190 nm, 25 °C), *t*_minor_ = 9.0 min; *t*_major_ = 14.3 min. ^1^H NMR (400 MHz, CDCl_3_) δ 9.97 (s, 1H), 7.53–7.46 (m, 1H), 7.47–7.40 (m, 1H), 7.36–7.27 (m, 6H), 7.15 (d, *J* = 7.7 Hz, 1H), 6.07 (s, 1H), 4.53 (t, *J* = 1.7 Hz, 1H), 3.87 (d, *J* = 19.4 Hz, 1H), 3.77 (s, 3H), 2.96 (dt, *J*_1_ = 19.3, *J*_2_ = 1.5 Hz, 1H), 2.35 (s, 3H). ^13^C{^1^H} NMR (101 MHz, CDCl_3_) δ: 186.5, 173.4, 166.9, 162.6, 138.6, 136.1, 133.2, 132.1, 131.8, 131.7, 130.9, 128.7 (2C), 127.7, 126.8 (2C), 123.6, 64.2, 57.2, 53.4, 49.8, 14.6. IR (KBr): ν = 2951, 1738, 1635, 1525, 1433, 1203 cm^–1^; [α]^25^_D_ = +4.7° (1.28, CHCl_3_); HRMS (ESI-TOF) *m*/*z*: [M + Na]^+^ calcd for C_22_H_20_BrNO_4_Na 464.0468; found 464.0465.

#### Methyl (1*S*,2*R*)-1-Benzamido-3-formyl-4-methyl-2-(naphthalen-2-yl)cyclopent-3-ene-1-carboxylate (**4j**)

The title compound was synthesized according to the general procedure and purified by column chromatography (hexane/EtOAc 3:2): yellowish foam, yield 52% (26 mg), ee 97%. The ee was determined by HPLC analysis using a Chiralpak IA column (80:20 heptane/i-PrOH, flow rate 1.0 mL/min; λ = 190 nm, 25 °C), *t*_minor_ = 18.8 min; *t*_major_ = 23.2 min. ^1^H NMR (400 MHz, CDCl_3_) δ 10.04 (s, 1H), 7.96–7.80 (m, 3H), 7.73 (s, 1H), 7.58–7.51 (m, 2H), 7.38–7.28 (m, 2H), 7.19–7.11 (m, 4H), 6.16 (s, 1H), 4.73 (s, 1H), 4.03 (d, *J* = 19.3 Hz, 1H), 3.83 (s, 3H), 3.03 (dt, *J*_1_ = 19.4, *J*_2_ = 1.6 Hz, 1H), 2.43 (s, 3H). ^13^C{^1^H} NMR (101 MHz, CDCl_3_) δ: 186.7, 173.7, 166.7, 162.6, 136.3, 133.6, 133.3, 133.3, 133.2, 131.8, 129.4, 128.7, 128.5 (2C), 128.1, 127.9, 127.3, 126.9, 126.7 (3C), 64.2, 58.0, 53.3, 50.0, 14.6. IR (KBr): ν = 2951, 1736, 1662, 1529, 1203 cm^–1^; [α]^25^_D_ = +3.0° (0.84, CHCl_3_); HRMS (ESI-TOF) *m*/*z*: [M + Na]^+^ calcd for C_26_H_23_NO_4_Na 436.1519; found 436.1520.

#### Methyl (1*S*,2*R*)-1-Benzamido-2-ethyl-3-formyl-4-methylcyclopent-3-ene-1-carboxylate (**4l**)

The title compound was synthesized according to the general procedure and purified by column chromatography (hexane/EtOAc 3:2): yellowish foam, yield 66% (25 mg), ee 85%. The ee was determined by HPLC analysis using a Chiralpak IA column (80:20 heptane/i-PrOH, flow rate of 1.0 mL/min; λ = 190 nm, 25 °C), *t*_minor_ = 8.7 min; *t*_major_ = 14.7 min. ^1^H NMR (400 MHz, CDCl_3_) δ 9.97 (s, 1H), 7.80–7.74 (m, 2H), 7.55–7.49 (m, 1H), 7.48–7.41 (m, 2H), 6.99 (s, 1H), 3.70 (s, 3H), 3.53 (d, *J* = 19.6 Hz, 1H), 3.48–3.43 (m, 1H), 2.98 (dt, *J*_1_ = 18.9, *J*_2_ = 1.5 Hz, 1H), 2.19 (s, 3H), 1.80 (p, *J* = 7.0 Hz, 2H), 0.94 (t, *J* = 7.5 Hz, 3H). ^13^C{^1^H} NMR (101 MHz, CDCl_3_) δ: 187.5, 174.3, 167.6, 160.6, 137.3, 133.9, 132.1, 128.8 (2C), 127.1 (2C), 65.0, 53.1, 51.8, 49.7, 21.5, 14.3, 11.7. IR (KBr): ν = 2952, 1738, 1639, 1525, 1317, 1207 cm^–1^; [α]^25^_D_ = +71.4° (1.05, CHCl_3_); HRMS (ESI-TOF) *m*/*z*: [M + Na]^+^ calcd for C_18_H_21_NO_4_Na 338.1363; found 338.1364.

#### Methyl (1*S*,2*R*)-1-Benzamido-3-formyl-4-methyl-2-propylcyclopent-3-ene-1-carboxylate (**4m**)

The title compound was synthesized according to the general procedure and purified by column chromatography (hexane/EtOAc 3:2): yellowish foam, yield 65% (26 mg), ee 94%. The ee was determined by HPLC analysis using a Chiralpak IA column (80:20 heptane/i-PrOH, flow rate 1.0 mL/min; λ = 190 nm, 25 °C), *t*_minor_ = 8.6 min; *t*_major_ = 12.5 min. ^1^H NMR (400 MHz, CDCl_3_) δ 9.97 (s, 1H), 7.79–7.73 (m, 2H), 7.56–7.50 (m, 1H), 7.49–7.41 (m, 2H), 6.94 (s, 1H), 3.72 (s, 3H), 3.54 (d, *J* = 18.8 Hz, 1H), 3.50–3.43 (m, 1H), 3.01 (dt, *J* = 18.9, 1.5 Hz, 1H), 2.19 (d, *J* = 1.3 Hz, 3H), 1.81–1.59 (m, 2H), 1.44–1.30 (m, 2H), 0.96–0.85 (m, 3H). ^13^C{^1^H} NMR (101 MHz, CDCl_3_) δ: 187.5, 174.4, 167.5, 160.4, 137.7, 134.0, 132.1, 128.9 (2C), 127.0 (2C), 65.2, 53.2, 50.9, 49.6, 30.9, 20.9, 14.5, 14.3. IR (KBr): ν = 2954, 1738, 1635, 1529, 1321, 1205 cm^–1^; [α]^25^_D_ = +69.1° (0.81, CHCl_3_); HRMS (ESI-TOF) *m*/*z*: [M + Na]^+^ calcd for C_19_H_23_NO_4_Na 352.1519; found 352.1520.

#### Methyl (1*S*,2*R*)-1-Benzamido-2-butyl-3-formyl-4-methylcyclopent-3-ene-1-carboxylate (**4n**)

The title compound was synthesized according to the general procedure and purified by column chromatography (hexane/EtOAc 3:2): yellowish foam, yield 70% (29 mg), ee 92%. The ee was determined by HPLC analysis using a Chiralpak IA column (80:20 heptane/i-PrOH, flow rate 1.0 mL/min; λ = 190 nm, 25 °C), *t*_minor_ = 8.6 min; *t*_major_ = 12.4 min. ^1^H NMR (400 MHz, CDCl_3_) δ 9.96 (s, 1H), 7.78–7.73 (m, 2H), 7.55–7.49 (m, 1H), 7.48–7.39 (m, 2H), 6.98 (s, 1H), 3.71 (s, 3H), 3.53 (d, *J* = 19.0 Hz, 1H), 3.00 (dt, *J*_1_ = 18.9, *J*_2_ = 1.5 Hz, 1H), 2.18 (q, *J* = 1.4 Hz, 3H), 1.79–1.67 (m, 2H), 1.35–1.26 (m, 2H), 0.89–0.84 (m, 3H). ^13^C{^1^H} NMR (101 MHz, CDCl_3_) δ: 187.5, 174.4, 167.5, 160.2, 137.7, 134.0, 132.0, 128.8 (2C), 127.0 (2C), 65.1, 53.1, 50.9, 49.6, 29.6, 28.3, 23.1, 14.3, 14.0. IR (KBr): ν = 2952, 1739, 1635, 1527, 1315, 1203 cm^–1^; [α]^25^_D_ = +70.9° (1.19, CHCl_3_); HRMS (ESI-TOF) *m*/*z*: [M + Na]^+^ calcd for C_20_H_25_NO_4_Na 366.1676; found 366.1677.

#### Methyl (1*S*,2*R*)-1-(4-Bromobenzamido)-3-formyl-4-methyl-2-phenylcyclopent-3-ene-1-carboxylate (**4o**)

The title compound was synthesized according to the general procedure and purified by column chromatography (hexane/EtOAc 3:2): yellowish foam, yield 46% (26 mg), ee 93%. The ee was determined by HPLC analysis using a Chiralpak IB column (70:30 heptane/i-PrOH, flow rate 1.0 mL/min; λ = 190 nm, 25 °C), *t*_minor_ = 9.2 min; *t*_major_ = 12.7 min. ^1^H NMR (400 MHz, CDCl_3_) δ 9.98 (s, 1H), 7.48–7.35 (m, 5H), 7.22–7.17 (m, 2H), 7.12–7.04 (m, 2H), 5.98 (s, 1H), 4.52 (s, 1H), 3.93 (d, *J* = 19.3 Hz, 1H), 3.77 (s, 3H), 2.91 (dt, *J* = 19.3, 1.6 Hz, 1H), 2.36 (d, *J* = 1.3 Hz, 3H). ^13^C{^1^H} NMR (101 MHz, CDCl_3_) δ: 186.7, 173.5, 165.7, 162.3, 136.2, 135.9, 132.1, 132.0, 131.9 (2C), 129.6 (2C), 128.9, 128.8, 128.3 (2C), 126.7, 63.9, 57.9, 53.3, 49.9, 14.6. IR (KBr): ν = 2951, 1738, 1635, 1481, 1340, 1205 cm^–1^; [α]^25^_D_ = +53.6° (0.69, CHCl_3_); HRMS (ESI-TOF) *m*/*z*: [M + Na]^+^ calcd for C_19_H_20_NO_4_Na 464.0468; found 464.0473.

#### Methyl (1*S*,2*R*)-3-Formyl-4-methyl-2-phenyl-1-pivalamidocyclopent-3-ene-1-carboxylate (**4s**)

The title compound was synthesized according to the general procedure and purified by column chromatography (hexane/EtOAc 3:2): yellowish foam, yield 37% (15 mg), ee 87%. The ee was determined by HPLC analysis using a Chiralpak IA column (80:20 heptane/i-PrOH, flow rate of 1.0 mL/min; λ = 190 nm, 25 °C), *t*_minor_ = 6.2 min; *t*_major_ = 8.0 min. ^1^H NMR (400 MHz, CDCl_3_) δ 9.98 (s, 1H), 7.45–7.31 (m, 3H), 7.15 (d, *J* = 7.2 Hz, 2H), 5.55 (s, 1H), 4.44 (s, 1H), 3.85 (d, *J* = 19.4 Hz, 1H), 3.76 (s, 3H), 2.77 (dt, *J*_1_ = 19.4, *J*_2_ = 1.6 Hz, 1H), 2.34 (s, 3H), 0.86 (s, 9H). ^13^C{^1^H} NMR (101 MHz, CDCl_3_) δ: 186.7, 178.2, 173.9, 162.4, 136.3, 136.1, 129.4 (3C), 128.6 (2C), 63.4, 57.7, 53.1, 50.3, 27.5, 26.9 (3C), 14.5. IR (KBr): ν = 2952, 1720, 1653, 1508, 1435, 1201 cm^–1^; [α]^25^_D_ = −27.3° (0.55, CHCl_3_); HRMS (ESI-TOF) *m*/*z*: [M + Na]^+^ calcd for C_20_H_25_NO_4_Na 366.1676; found 366.1675.

### Preparation of Compounds **5a** and **6a**

#### Methyl (1*S*,2*R*)-1-Benzamido-3-((*E*)-3-ethoxy-3-oxoprop-1-en-1-yl)-4-methyl-2-phenylcyclopent-3-ene-1-carboxylate (**5a**)

The ylide reagent (0.4127 mmol, 144 mg, 5 equiv) was added to the compound **4a** (0.0826 mmol, 30 mg, 1 equiv) dissolved in CH_2_Cl_2_ (2 mL). The reaction mixture was stirred at room temperature until full conversion was reached (TLC monitoring). Product **5a** was isolated after silica separation in Hex/EtOAc (1:1) as a white solid foam: yield 56% (20 mg), ee 96%. The ee was determined by HPLC analysis using a Chiralpak IA column (70:30 heptane/i-PrOH, flow rate 1.0 mL/min; λ = 190 nm, 25 °C), *t*_minor_ = 5.8 min; *t*_major_ = 8.8 min. ^1^H NMR (400 MHz, CDCl_3_) δ 7.57 (d, *J* = 15.8 Hz, 1H), 7.46–7.36 (m, 4H), 7.30–7.19 (m, 6H), 6.01 (s, 1H), 5.38 (d, *J* = 15.8 Hz, 1H), 4.44 (s, 1H), 4.19–4.05 (m, 2H), 3.77 (s, 3H), 3.75 (d, *J* = 18.7 Hz, 1H), 2.78 (dd, *J*_1_ = 18.7, *J*_2_ = 1.7 Hz, 1H), 2.11 (d, *J* = 1.5 Hz, 3H), 1.22 (t, *J* = 7.1 Hz, 3H). ^13^C{^1^H} NMR (101 MHz, CDCl_3_) δ: 174.0, 167.4, 166.6, 149.4, 136.3 (2C), 133.5, 131.8, 131.5, 129.7 (2C), 129.1 (2C), 128.7, 128.6 (2C), 126.8 (2C), 119.1, 64.8, 60.4, 59.3, 53.2, 48.8, 14.8, 14.4. IR (KBr): ν = 2947, 1712, 1657, 1522, 1304, 1153 cm^–1^; [α]^25^_D_ = +69.5° (0.77, CHCl_3_); HRMS (ESI-TOF) *m*/*z*: [M + Na]^+^ calcd for C_26_H_27_NO_5_Na 456.1781; found 456.1782.

#### Methyl (1*S*,2*R*)-1-Benzamido-3-(hydroxymethyl)-4-methyl-2-phenylcyclopent-3-ene-1-carboxylate (**6a**)

NaBH_4_ (0.1652 mmol, 6.3 mg, 2 equiv) was added to a solution of compound **4a** (0.0826 mmol, 30 mg, 1 equiv) in methanol (1.5 mL) at 0 °C. The reaction mixture was stirred at room temperature until full conversion was reached (TLC monitoring). The reaction mixture was then evaporated, and the crude product was purified on silica gel in Hex/EtOAc (1:1). The corresponding alcohol derivative **6a** was obtained as white solid foam: yield 70% (21 mg), ee 92%. The ee was determined by HPLC analysis using a Chiralpak IA column (70:30 heptane/i-PrOH, flow rate 1.0 mL/min; λ = 250 nm, 25 °C), *t*_minor_ = 7.4 min; *t*_major_ = 9.7 min. ^1^H NMR (400 MHz, CDCl_3_) δ 7.46–7.33 (m, 4H), 7.30–7.21 (m, 6H), 6.03 (s, 1H), 4.49 (s, 1H), 4.33 (d, *J* = 12.5 Hz, 1H), 3.86 (d, *J* = 13.1 Hz, 1H), 3.77 (s, 3H), 3.59 (d, *J* = 17.2 Hz, 1H), 2.71 (d, *J* = 17.4 Hz, 1H), 1.89 (s, 3H). ^13^C{^1^H} NMR (101 MHz, CDCl_3_) δ: 174.5, 166.7, 137.6, 136.8, 133.7, 133.5, 131.7, 129.7 (2C), 129.4 (2C), 128.5 (3C), 126.8 (2C), 64.7, 60.3, 57.8, 53.1, 48.5, 14.0. IR (KBr): ν = 2924, 1732, 1637, 1525, 1248 cm^–1^; [α]^25^_D_ = −6.0° (0.67, CHCl_3_); HRMS (ESI-TOF) *m*/*z*: [M + Na]^+^ calcd for C_22_H_23_NO_4_Na 388.1519; found 388.1523.

## Data Availability

The data underlying this study are available in the published article and its Supporting Information.

## Supporting Information Available

Copies of ^1^H and ^13^C NMR spectra of **1b**, **1e**, **1f**, **1g**, **4a**–**4e**, **4g**, **4h**, **4j**, **4l**–**4o**, **4s**, **5a**, and **6a** and chiral HPCL chromatograms of **4a**–**4e**, **4g**, **4h**, **4j**, **4l**–**4o**, **4s**, **5a**, and **6a** (PDF)
FAIR data, including the primary NMR FID files, for compounds **1b**, **1e**, **1f**, **1g 4a**–**4e**, **4g**, **4h**, **4j**, **4l**–**4o**, **4s**, **5a**, and **6a** (ZIP)
