# Supplementary material for: Enantioselective Preparation of Cyclopentene-Based Amino Acids with a Quaternary Carbon Center
Source: J Org Chem. 2024 Oct 29;89(22):16522–30. doi: 10.1021/acs.joc.4c01764 (PMC11574854; doi:10.1021/acs.joc.4c01764)
Supplement: Supplementary file 1 — jo4c01764_si_001.pdf [file jo4c01764_si_001.pdf]

Supporting information

Enantioselective Preparation of Cyclopentene-Based Amino Acids With Quaternary Carbon Center

Michael Franc,[a] Pavel Měrka,[a] Ivana Císařová [b] and Jan Veselý *[a]

[a] Department of Organic Chemistry, Faculty of Science, Charles University, Hlavova 2030, 128 43 Praha 2, Czech Republic.

[b] Department of Inorganic Chemistry, Faculty of Science, Charles University, Hlavova 2030, 128 43 Praha 2, Czech Republic

E-mail: jxvesely@natur.cuni.cz

General information

1 Starting compounds preparation S-3

2 Optimization of reaction condition for asymmetric reaction S-5

3 Asymmetric preparation of amino acid derivatives with chiral quaternary carbon center S-7

4 Further transformations S-15

4.1 Wittig reaction of compound **4a** S-15

4.2 Preparation of alcohol **6a** S-16

5 Crystalographic data for 4g S-17

6 NMR spectra **^{1}H NMR** (400 MHz, $CDCl_3$) S-27

7 HPLC traces S-46

General information

Chemicals and solvents were either purchased (puriss p.A.) from commercial suppliers or purified by standard techniques. For thin-layer chromatography (TLC), silica gel plates Merck 60 F254 were used, and compounds were visualized by irradiation with UV light and/or by treatment with a solution of phosphomolybdenic acid (25 g), $Ce(SO_4)_2 \cdot H_2O$ (10 g), conc. H_2SO_4 (60 mL), and H_2O (940 mL) followed by heating. Column chromatography was performed using silica gel Merck 60 (particle size 0.040–0.063 mm). ^1H NMR, ^{13}C NMR, 2D NMR were recorded with a Bruker DPX400 NMR. Chemical shifts (δ) are reported in ppm relative to residual solvent signals ($CHCl_3$, 7.26 ppm for ^1H NMR; $CDCl_3$, 77.2 ppm for ^{13}C NMR). High-resolution mass spectra were recorded on an LCQ Fleet spectrometer using a Bruker Compact QTOF-MS controlled by the Compass 1.9 Control software to measure the ESI high-resolution mass spectrums. The monoisotopic mass values were calculated using Data analysis software v 4.4. The analysis was conducted in the positive ion mode at a scan range from m/z 50 to 1000, and nitrogen was used as nebulizer gas at a pressure of 4 psi and flow of 3 l/min for the dry gas. The capillary voltage and temperature were set at 4500 V and 220 °C, respectively. Optical rotations were performed on an AU-Tomatica polarimeter, Autopol III. IR DRIFT spectras were recorded with Nicolet AVATAR 370 FT-IR in cm^{-1}. The HPLC analysis were performed on a LC20AD Shimadzu liquid chromatograph with SPD-M20A diode array detector with columns Daicel Chiralpak.

1 Starting compounds preparation

Compound **1a-g** were prepared according published four steps procedure.[1] Azlactones **1a**, **1c** and **1d** are known compounds.[1,2]Newly prepared azlactones **1b**, **1e**, **1f** and **1g**:

2-(4-Bromophenyl)-4-(prop-2-yn-1-yl)oxazol-5(4*H*)-one (1b)

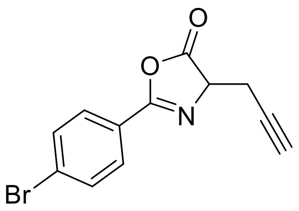

^{1}H NMR (400 MHz, $CDCl_3$) δ 7.89 (d, *J* = 8.6 Hz, 2H), 7.64 (d, *J* = 8.6 Hz, 2H), 4.53 (t, *J* = 5.3 Hz, 1H), 2.92 (qdd, J_1 = 16.9, J_2 = 5.3, J_3 = 2.6 Hz, 2H), 2.02 (t, *J* = 2.6 Hz, 1H). **^{13}C{^{1}H} NMR** (101 MHz, $CDCl_3$) δ 176.3, 162.3, 132.4 (2C), 129.7 (2C), 128.2, 124.6, 77.4, 72.0, 64.3, 21.7. **IR** (ATR): ν = 3275, 1817, 1651, 1485, 1319 cm^{-1}. **HRMS** (ESI-TOF) m/z: $[M+H]^+$ Calcd for $C_{12}H_9BrNO_2$ 277.9811; Found 277.9812.

4-(Prop-2-yn-1-yl)-2-(thiophen-2-yl)oxazol-5(4*H*)-one (1e)

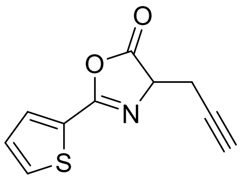

^{1}H NMR (400 MHz, $CDCl_3$) δ 7.75 (dd, J_1 = 3.8, J_2 = 1.3 Hz, 1H), 7.62 (dd, J_1 = 5.0, J_2 = 1.2 Hz, 1H), 7.16 (dd, J_1 = 5.0, J_2 = 3.8 Hz, 1H), 4.53 (t, *J* = 5.2 Hz, 1H), 2.98 – 2.83 (m, 2H), 2.03 (t, *J* = 2.6 Hz, 1H). **^{13}C {^{1}H} NMR** (101 MHz, $CDCl_3$) δ 176.1, 158.7, 132.6, 132.4 (2C), 128.3, 77.4, 72.0, 64.1, 21.7. **IR** (ATR): ν = 3271, 1809, 1645, 1537, 1311 cm^{-1}. **HRMS** (ESI-TOF) m/z: $[M+H]^+$ Calcd for $C_{10}H_8NO_2S$ 206.0607; Found 206.0611.

2-(tert-Butyl)-4-(prop-2-yn-1-yl)oxazol-5(4*H*)-one (1f)

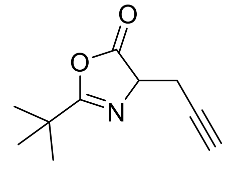

^{1}H NMR (400 MHz, $CDCl_3$) δ 4.29 (t, *J* = 4.8 Hz, 1H), 2.90 – 2.73 (m, 2H), 1.99 (t, *J* = 2.6 Hz, 1H), 1.29 (s, 9H). **^{13}C {^{1}H} NMR** (101 MHz, $CDCl_3$) δ 177.4, 173.6, 77.3, 71.6, 63.3, 34.4, 26.9 (3C), 21.4. **IR** (ATR): ν = 3219, 1824, 1658, 1479, 1281 cm^{-1}. **HRMS** (ESI-TOF) m/z: $[M+H]^+$ Calcd for $C_{10}H_{14}NO_2$ 180.1019; Found 180.1018.

4-(But-2-yn-1-yl)-2-phenyloxazol-5(4*H*)-one (1g)

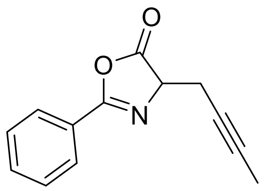

^{1}H NMR (400 MHz, $CDCl_3$) δ 8.06 – 8.00 (m, 2H), 7.62 – 7.55 (m, 1H), 7.49 (t, *J* = 7.7 Hz, 2H), 4.50 (t, *J* = 5.2 Hz, 1H), 2.96 – 2.74 (m, 2H), 1.69 (t, *J* = 2.5 Hz, 3H). **^{13}C {^{1}H} NMR** (101 MHz, $CDCl_3$) δ 177.2, 162.7, 133.0, 128.9 (2C), 128.2

[1] Žabka, M.; Kocian, A.; Bilka, S.; Andrejčák, S.; Šebesta, R. Transformation of Racemic Azlactones into Enantioenriched Dihydropyrroles and Lactones Enabled by Hydrogen-Bond Organocatalysis. *Eur. J. Org. Chem.* **2019**, 6077–6087.
[2] Oh, J.-S.; Kim, K. I.; Song C. E. Enantioselective synthesis of α-deuterium labelled chiral α-amino acids *via* dynamic kinetic resolution of racemic azlactones. *Org. Biomol. Chem.* **2011**, *9*, 7983–7985.

(2C), 125.9, 79.5, 72.2, 64.9, 22.2, 3.6. **IR** (ATR): ν = 2920, 1803, 1653, 1452, 1159 cm^{-1}. **HRMS** (ESI) m/z: $[M+H]^+$ Calcd for $C_{13}H_{12}NO_2$ 214.0863; Found 214.0862.

2 Optimization of reaction condition for asymmetric reaction

Table S1. Catalyst screening.

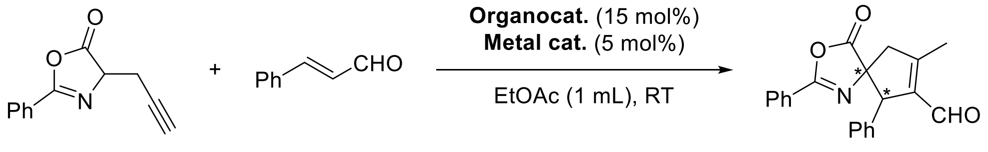

1a: 1.5 equiv. **2a**: 1.0 equiv. **3a**

Entry	Metal cat.	Organocat.	Time (d)	*Dr*[a]	Yield (%)	*Ee* (%)[b]
1	$Pd_2(dba)_3$	**I**	1	3:1	87	96/99
2	$Pd_2(dba)_3$	**II**	6	1.7:1	49	91/99
3	$Pd_2(dba)_3$	**V**	6	1.6:1	25	78/62
4	$Pd_2(dba)_3$	**IV**	6	-	n.r.	-
5	$Pd_2(dba)_3$	**VII**	6	1.8:1	traces	n.d.
6	$Pd_2(dba)_3$	**VI**	6	3.4:1	22	79/85
7	$Pd_2(dba)_3$	**III**	6	-	n.r.	-
8	$Pd(PPh_3)_4$	**I**	2	2.4:1	29	93/96
9	$PdCl_2$	**I**	6	2.8:1	20	85/86
10	$Pd(OAc)_2$	**I**	1	2.4:1	59	98/98
11	$AuNTf_2$	**I**	6	3:1	traces	n.d.
12	PPh_3AuNTf_2	**I**	6	1.1:1	52	87/94
13	PPh_3AuCl	**I**	6	-	n.r.	-

[a] determined by ^{1}H NMR; [b] determined by Chiral HPLC

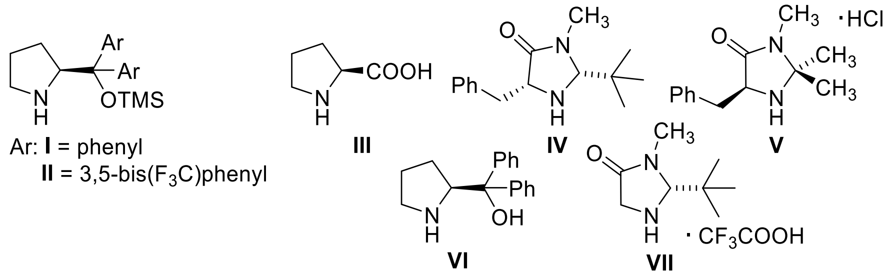

Figure S1. Used chiral secondary amines.

Table S2. Solvent screening.

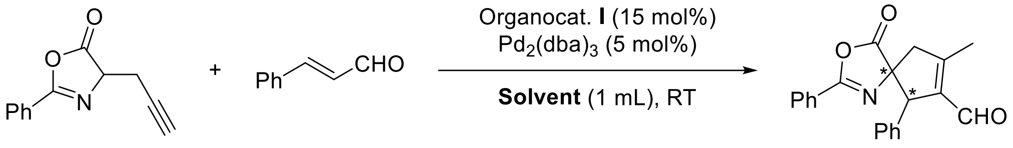

1a: 1.5 equiv. **2a**: 1.0 equiv. **3a**

Entry	Solvent	Time (d)	*Dr*[a]	Yield (%)	*Ee* (%)[b]
1	EtOAc	1	3:1	87	96/99
2	CH_3CN	6	3:1	30	86/96
3	Acetone	2	3:1	76	97/98
4	Toluene	1	2.5:1	93	95/98
5	CH_2Cl_2	2	2.6:1	81	96/98
6	$CHCl_3$	6	2.9:1	43	94/95
7	THF	4	2.5:1	68	93/94
8	TBME	4	2.8:1	69	97/99
9	MeOH	1	-	n.d.	-

[a] determined by ^1H NMR; [b] determined by Chiral HPLC; * decomposition of starting material

Table S3. Catalyst loading screening.

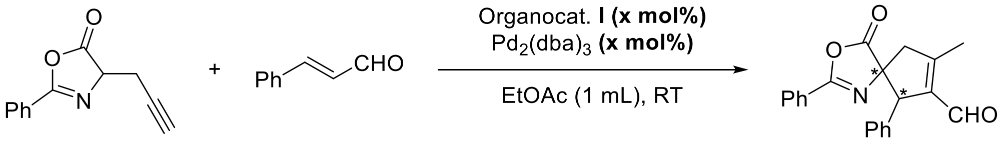

1a: 1.5 equiv. **2a**: 1.0 equiv. **3a**

Entry	Organocat. I (mol%)	$Pd_2(dba)_3$ (mol%)	Time (d)	*Dr*[a]	Yield (%)	*Ee* (%)[b]
1	15	5.0	1	3:1	87	96/99
2	10	5.0	6	3:1	70	96/97
3	5	5.0	6	3:1	33	95/93
4	15	2.0	1	3:1	87	96/99
5	15	1.0	1	3.2:1	80	98/99
6	10	2.0	4	2.8:1	60	98/99
7	10	1.0	4	3:1	61	98/99
8	5	1.0	6	3.1:1	43	96/99

[a] determined by ^1H NMR; [b] determined by Chiral HPLC; * decomposition of starting material

3 Asymmetric preparation of amino acid derivatives with chiral quaternary carbon center

General procedure of synthesis compounds 4

The corresponding *α*,*β*-unsaturated aldehyde **2** (0.12 mmol, 1 equiv), azlactone derivative **1** (0.18 mmol, 1.5 equiv.), $Pd_2(dba)_3$ (0.0012 mmol, 0.01 equiv.) were added to solution of organic catalyst 2-(diphenyl((trimethylsilyl)oxy)methyl)pyrrolidine (0.018 mmol, 0.15 equiv) in EOAc (0.5 mL). The reaction mixture was stirred at room temperature and checked by ^{1}H NMR. After full conversion crude mixture was evaporated. Crude product was dissolved in MeOH (1ml), and trimethylsilyl chloride (0.36 mol, 39 mg, 3 equiv) was added to this solution. The reaction mixture was stirred at 45 °C (oil bath) for 2 h. The solvent was evaporated and product **4** was isolated after silica separation in Hex/EtOAc.

Scale up synthesis of compound 4a

The corresponding *α*,*β*-unsaturated aldehyde **2a** (1.2 mmol, 1 equiv), azlactone derivative **1a** (1.8 mmol, 1.5 equiv.), $Pd_2(dba)_3$ (0.012 mmol, 0.01 equiv.) were added to solution of organic catalyst 2-(diphenyl((trimethylsilyl)oxy)methyl)pyrrolidine **I** (0.18 mmol, 0.15 equiv) in EtOAc (5 mL). The reaction mixture was stirred at room temperature and checked by ^{1}H NMR. After full conversion crude mixture was evaporated. Crude product was dissolved in MeOH (1ml), and trimethylsilyl chloride (3.6 mol, 3 equiv) was added to this solution. The reaction mixture was stirred at 45 °C (oil bath) for 2 h. The solvent was evaporated and product **4a** was isolated after silica separation in Hex/EtOAc (3:2). Compound **4a** was obtained as an yellowish foam in yield 47 % (205 mg) with *ee* 94 %.

Methyl (1*S*,2*R*)-1-benzamido-3-formyl-4-methyl-2-phenylcyclopent-3-ene-1-carboxylate (4a)

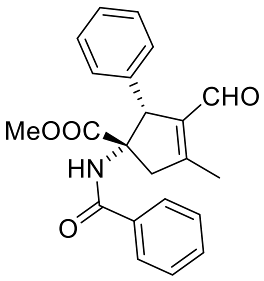

The title compound was synthesized according to the general procedure and purified by column chromatography (hexane/EtOAc - 3:2). Yellowish foam, yield 56 % (24 mg), *ee* 94 %. The ee was determined by HPLC analysis using Chiralpak IA column (80/20 heptane/i-PrOH, flow rate 1.0 ml/min; λ = 190 nm, 25 °C), t_{minor} = 14.3 min; t_{major} = 18.0 min. **^{1}H NMR** (400 MHz, $CDCl_3$) δ 9.99 (s, 1H), 7.49 – 7.37 (m, 5H), 7.31 – 7.20 (m, 5H), 6.06 (s, 1H), 4.52 (s, 1H), 3.97 (d, *J* = 19.2 Hz, 1H), 3.77 (s, 3H), 2.92 (dt, J_1 = 19.3, J_2 = 1.6 Hz, 1H), 2.36 (d, *J* = 1.3 Hz, 3H). **^{13}C {^{1}H} NMR** (101 MHz, $CDCl_3$) δ 186.7, 173.7, 166.6, 162.5, 136.2, 136.0, 133.2, 131.9, 129.6 (2C), 128.8 (2C), 128.6 (2C), 127.3, 126.8 (2C), 64.0, 57.9, 53.3, 49.9, 14.6. **IR** (ATR): ν = 2951, 1736, 1639, 1525, 1205 cm^{-1}; **$[\alpha]^{25}_{D}$** = +54.1 °, (0.85, $CHCl_3$); **HRMS** (ESI-TOF) m/z: $[M+Na]^+$ Calcd for $C_{22}H_{21}NO_4Na$ 386.1363; Found 386.1365.

Methyl (1*S*,2*R*)-1-benzamido-3-formyl-2-(4-methoxyphenyl)-4-methylcyclopent-3-ene-1-carboxylate (4b)

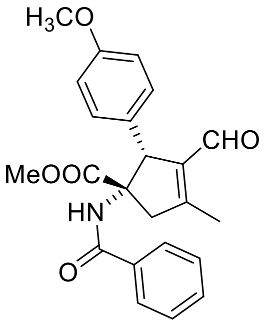

The title compound was synthesized according to the general procedure and purified by column chromatography (hexane/EtOAc - 3:2). Yellowish foam, yield 43 % (20 mg), *ee* 95 %. The ee was determined by HPLC analysis using Chiralpak IA column (70/30 heptane/i-PrOH, flow rate 1.0 ml/min; λ = 190 nm, 25 °C), t_{minor} = 9.7 min; t_{major} = 14.2 min. **^{1}H NMR** (400 MHz, $CDCl_3$) δ 9.97 (s, 1H), 7.47 – 7.27 (m, 5H), 7.13 (d, *J* = 8.1 Hz, 2H), 6.99 – 6.93 (m, 2H), 6.13 (s, 1H), 4.47 (s, 1H), 3.95 (d, *J* = 20.3 Hz, 1H), 3.83 (s, 3H), 3.76 (s, 3H), 2.90 (dt, J_1 = 19.3, J_2 = 1.6 Hz, 1H), 2.34 (q, *J* = 1.3 Hz, 3H). **^{13}C{^{1}H} NMR** (101 MHz, $CDCl_3$) δ 186.8, 173.8, 166.6, 162.3, 159.9, 136.4, 133.3, 131.9, 130.0 (2C), 128.7 (2C), 127.7, 126.8 (2C), 115.0 (2C), 64.0, 57.2, 55.5, 53.2, 49.8, 14.6. **IR** (ATR): ν = 2951, 1734, 1631, 1510, 1205 cm^{-1}; **$[\alpha]^{25}_{D}$** = +41.7 °, (0.60, $CHCl_3$); **HRMS** (ESI-TOF) m/z: $[M+Na]^+$ Calcd for $C_{23}H_{23}NO_5Na$ 416.1468; Found 416.1467.

Methyl (1*S*,2*R*)-1-benzamido-3-formyl-4-methyl-2-(p-tolyl)cyclopent-3-ene-1-carboxylate (4c)

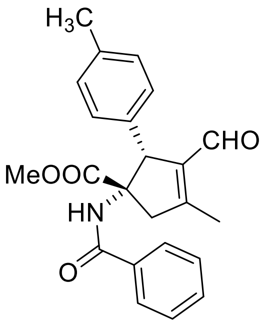

The title compound was synthesized according to the general procedure and purified by column chromatography (hexane/EtOAc - 3:2). Yellowish foam, yield 48 % (22 mg), *ee* 95 %. The ee was determined by HPLC analysis using Chiralpak IA column (80/20 heptane/i-PrOH, flow rate 1.0 ml/min; λ = 190 nm, 25 °C), t_{minor} = 12.0 min; t_{major} = 15.3 min. **^{1}H NMR** (400 MHz, $CDCl_3$) δ 9.98 (s, 1H), 7.46 – 7.39 (m, 1H), 7.33 – 7.22 (m, 6H), 7.09 (d, *J* = 7.5 Hz, 2H), 6.11 (s, 1H), 4.48 (t, *J* = 1.7 Hz, 1H), 3.95 (d, *J* = 19.2 Hz, 1H), 3.76 (s, 3H), 2.90 (dt, J_1 = 19.3, J_2 = 1.6 Hz, 1H), 2.38 (s, 3H), 2.35 (q, *J* = 1.3 Hz, 3H). **^{13}C{^{1}H} NMR** (101 MHz, $CDCl_3$) δ 186.8, 173.8, 166.6, 162.4, 138.6, 136.2, 133.3, 132.7, 131.9, 130.2 (2C), 128.7 (2C), 128.6 (2C), 126.8 (2C), 63.9, 57.6, 53.2, 49.9, 21.3, 14.6. **IR** (ATR): ν = 2951, 1736, 1639, 1514, 1205 cm^{-1}; **$[\alpha]^{25}_D$** = +37.2 °, (0.90, $CHCl_3$); **HRMS** (ESI) m/z: $[M+Na]^+$ Calcd for $C_{23}H_{23}NO_4Na$ 400.1519; Found 400.1515.

Methyl (1*S*,2*R*)-1-benzamido-3-formyl-4-methyl-2-(4-nitrophenyl)cyclopent-3-ene-1-carboxylate (4d)

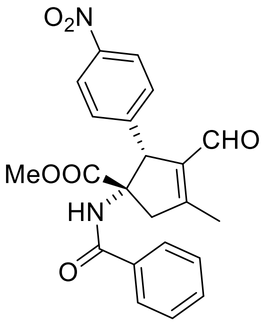

The title compound was synthesized according to the general procedure and purified by column chromatography (hexane/EtOAc - 3:2). Yellowish foam, yield 49 % (24 mg), *ee* 97 %. The ee was determined by HPLC analysis using Chiralpak IA column (70/30 heptane/i-PrOH, flow rate 1.0 ml/min; λ = 190 nm, 25 °C), t_{minor} = 11.7 min; t_{major} = 21.4 min. **^{1}H NMR** (400 MHz, $CDCl_3$) δ 9.97 (s, 1H), 8.20 – 8.12 (m, 2H), 7.48 – 7.25 (m, 7H), 6.23 – 6.11 (m, 1H), 4.82 (s, 1H), 3.81 – 3.79 (m, 3H), 3.76 – 3.65 (m, 1H), 3.14 (d, *J* = 21.2 Hz, 1H), 2.36 (q, *J* = 1.5 Hz, 3H). **^{13}C{^{1}H} NMR** (101 MHz, $CDCl_3$) δ 186.4, 173.3, 167.3, 161.6, 147.6, 144.4, 136.9, 133.1, 132.2, 130.0 (2C), 128.8 (2C), 126.6 (2C), 124.0 (2C), 65.0, 56.9, 53.5, 49.8, 14.6. **IR** (ATR): ν = 2951, 1738, 1655, 1514, 1344, 1205 cm^{-1}; **$[\alpha]^{25}_D$** = -56.3 °, (1.11, $CHCl_3$); **HRMS** (ESI-TOF) m/z: $[M+Na]^+$ Calcd for $C_{22}H_{20}N_2O_6Na$ 431.1214; Found 431.1213.

Methyl (1*S*,2*R*)-1-benzamido-3-formyl-2-(4-formylphenyl)-4-methylcyclopent-3-ene-1-carboxylate (4e)

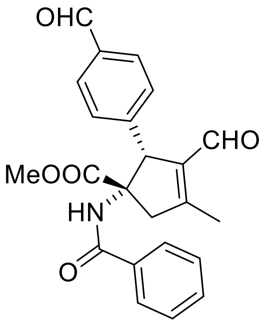

The title compound was synthesized according to the general procedure and purified by column chromatography (hexane/EtOAc - 1:1). Yellowish foam, yield 40 % (19 mg), *ee* n.d. The ee was not determined due to not finding suitable conditions using HPLC. **^{1}H NMR** (400 MHz, $CDCl_3$) δ 10.02 (s, 1H), 10.00 (s, 1H), 7.94 – 7.88 (m, 2H), 7.59 – 7.21 (m, 7H), 6.05 (s, 1H), 4.69 (s, 1H), 3.88 (d, *J* = 19.4 Hz, 1H), 3.81 (s, 3H), 3.06 (d, *J* = 19.4 Hz, 1H), 2.39 (s, 3H). **^{13}C{^{1}H} NMR** (101 MHz, $CDCl_3$) δ 191.6, 186.5, 173.4, 167.0, 162.3, 143.4, 136.5, 136.3, 133.2, 132.1, 130.5, 129.7, 128.8, 128.7 (2C), 127.5, 126.7 (2C), 64.6, 57.6, 53. 5, 50. 0, 14.6. **IR** (ATR): ν = 2951, 1736, 1655, 1525, 1435, 1209 cm^{-1}; **$[\alpha]^{25}_{D}$** = -28.6 °, (0.56, $CHCl_3$); **HRMS** (ESI-TOF) m/z: $[M+H]^+$ Calcd for $C_{23}H_{22}NO_5$ 392.1493; Found 392.1492.

Methyl (1*S*,2*R*)-1-benzamido-2-(4-bromophenyl)-3-formyl-4-methylcyclopent-3-ene-1-carboxylate (4g)

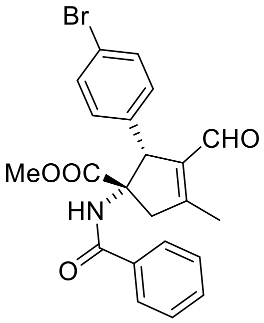

The title compound was synthesized according to the general procedure and purified by column chromatography (hexane/EtOAc - 3:2). Yellowish foam, yield 56 % (30 mg), *ee* 96 %. The ee was determined by HPLC analysis using Chiralpak IA column (70/30 heptane/i-PrOH, flow rate 1.0 ml/min; λ = 190 nm, 25 °C), t_{minor} = 9.2 min; t_{major} = 14.5 min. **^{1}H NMR** (400 MHz, $CDCl_3$) δ 9.96 (s, 1H), 7.53 (d, *J* = 8.6 Hz, 2H), 7.48 – 7.39 (m, 1H), 7.35 – 7.26 (m, 4H), 7.08 (d, *J* = 7.9 Hz, 2H), 6.04 (s, 1H), 4.52 (t, *J* = 1.7 Hz, 1H), 3.88 (d, *J* = 19.4 Hz, 1H), 3.77 (s, 3H), 2.96 (dt, J_1 = 19.4, J_2 = 1.5 Hz, 1H), 2.34 (q, *J* = 1.3 Hz, 3H). **^{13}C{^{1}H} NMR** (101 MHz, $CDCl_3$) δ 186.5, 173.5, 166.9, 162.4, 136.3, 135.2, 133.2, 132.5 (2C), 132.1, 130.5 (2C), 128.8 (2C), 126.7 (2C), 122.6, 64.1, 57.2, 53.3, 49.8, 14.6. **IR** (ATR): ν = 2951, 1736, 1637, 1527, 1487, 1203 cm^{-1}; **$[\alpha]^{25}_{D}$** = +6.1 °, (1.23, $CHCl_3$); **HRMS** (ESI-TOF) m/z: $[M+Na]^+$ Calcd for $C_{22}H_{20}BrNO_4Na$ 464.0468; Found 464.0471.

Methyl (1*S*,2*R*)-1-benzamido-2-(3-bromophenyl)-3-formyl-4-methylcyclopent-3-ene-1-carboxylate (4h)

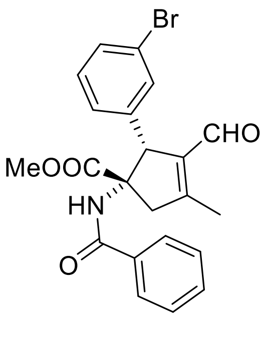

The title compound was synthesized according to the general procedure and purified by column chromatography (hexane/EtOAc - 3:2). Yellowish foam, yield 55 % (29 mg), *ee* 96 %. The ee was determined by HPLC analysis using Chiralpak IA column (70/30 heptane/i-PrOH, flow rate 1.0 ml/min; λ = 190 nm, 25 °C), t_{minor} = 9.0 min; t_{major} = 14.3 min. **^{1}H NMR** (400 MHz, $CDCl_3$) δ 9.97 (s, 1H), 7.53 – 7.46 (m, 1H), 7.47 – 7.40 (m, 1H), 7.36 – 7.27 (m, 6H), 7.15 (d, *J* = 7.7 Hz, 1H), 6.07 (s, 1H), 4.53 (t, *J* = 1.7 Hz, 1H), 3.87 (d, *J* = 19.4 Hz, 1H), 3.77 (s, 3H), 2.96 (dt, J_1 = 19.3, J_2 = 1.5 Hz, 1H), 2.35 (s, 3H). **^{13}C{^{1}H} NMR** (101 MHz, $CDCl_3$) δ 186.5, 173.4, 166.9, 162.6, 138.6, 136.1, 133.2, 132.1, 131.8, 131.7, 130.9, 128.7 (2C), 127.7, 126.8 (2C), 123.6, 64.2, 57.2, 53.4, 49.8, 14.6. **IR** (KBr): ν = 2951, 1738, 1635, 1525, 1433, 1203 cm^{-1}; **[α]$^{25}_D$** = +4.7 °, (1.28, $CHCl_3$); **HRMS** (ESI-TOF) m/z: $[M+Na]^+$ Calcd for $C_{22}H_{20}BrNO_4Na$ 464.0468; Found 464.0465.

Methyl (1*S*,2*R*)-1-benzamido-3-formyl-4-methyl-2-(naphthalen-2-yl)cyclopent-3-ene-1-carboxylate (4j)

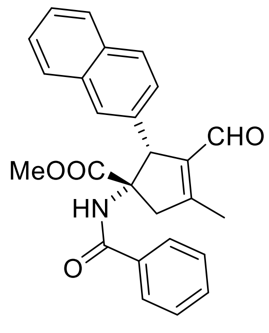

The title compound was synthesized according to the general procedure and purified by column chromatography (hexane/EtOAc - 3:2). Yellowish foam, yield 52 % (26 mg), *ee* 97 %. The ee was determined by HPLC analysis using Chiralpak IA column (80/20 heptane/i-PrOH, flow rate 1.0 ml/min; λ = 190 nm, 25 °C), t_{minor} = 18.8 min; t_{major} = 23.2 min. **^{1}H NMR** (400 MHz, $CDCl_3$) δ 10.04 (s, 1H), 7.96 – 7.80 (m, 3H), 7.73 (s, 1H), 7.58 – 7.51 (m, 2H), 7.38 – 7.28 (m, 2H), 7.19 – 7.11 (m, 4H), 6.16 (s, 1H), 4.73 (s, 1H), 4.03 (d, *J* = 19.3 Hz, 1H), 3.83 (s, 3H), 3.03 (dt, J_1 = 19.4, J_2 = 1.6 Hz, 1H), 2.43 (s, 3H). **^{13}C{^{1}H} NMR** (101 MHz, $CDCl_3$) δ 186.7, 173.7, 166.7, 162.6, 136.3, 133.6, 133.3, 133.3, 133.2, 131.8, 129.4, 128.7, 128.5 (2C), 128.1, 127.9, 127.3, 126.9, 126.7 (3C), 64.2, 58.0, 53.3, 50.0, 14.6. **IR** (KBr): ν = 2951, 1736, 1662, 1529, 1203 cm^{-1}; **[α]$^{25}_D$** = +3.0 °, (0.84, $CHCl_3$); **HRMS** (ESI-TOF) m/z: $[M+Na]^+$ Calcd for $C_{26}H_{23}NO_4Na$ 436.1519; Found 436.1520.

Methyl (1*S*,2*R*)-1-benzamido-2-ethyl-3-formyl-4-methylcyclopent-3-ene-1-carboxylate (4l)

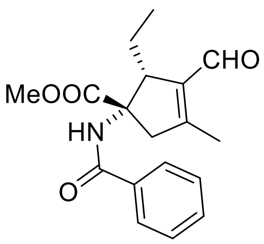

The title compound was synthesized according to the general procedure and purified by column chromatography (hexane/EtOAc - 3:2). Yellowish foam, yield 66 % (25 mg), *ee* 85 %. The ee was determined by HPLC analysis using Chiralpak IA column (80/20 heptane/i-PrOH, flow rate 1.0 ml/min; λ = 190 nm, 25 °C), t_{minor} = 8.7 min; t_{major} = 14.7 min. **^{1}H NMR** (400 MHz, $CDCl_3$) δ 9.97 (s, 1H), 7.80 – 7.74 (m, 2H), 7.55 – 7.49 (m, 1H), 7.48 – 7.41 (m, 2H), 6.99 (s, 1H), 3.70 (s, 3H), 3.53 (d, *J* = 19.6 Hz, 1H), 3.48 – 3.43 (m, 1H), 2.98 (dt, J_1 = 18.9, J_2 = 1.5 Hz, 1H), 2.19 (s, 3H), 1.80 (p, *J* = 7.0 Hz, 2H), 0.94 (t, *J* = 7.5 Hz, 3H). **^{13}C{^{1}H} NMR** (101 MHz, $CDCl_3$) δ 187.5, 174.3, 167.6, 160.6, 137.3, 133.9, 132.1, 128.8 (2C), 127.1 (2C), 65.0, 53.1, 51.8, 49.7, 21.5, 14.3, 11.7. **IR** (KBr): ν = 2952, 1738, 1639, 1525, 1317, 1207 cm^{-1}; **$[\alpha]^{25}_{D}$** = +71.4 °, (1.05, $CHCl_3$); **HRMS** (ESI-TOF) m/z: $[M+Na]^+$ Calcd for $C_{18}H_{21}NO_4Na$ 338.1363; Found 338.1364.

Methyl (1*S*,2*R*)-1-benzamido-3-formyl-4-methyl-2-propylcyclopent-3-ene-1-carboxylate (4m)

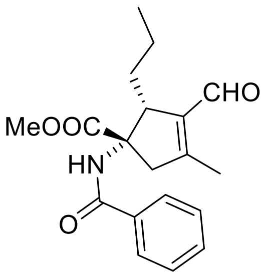

The title compound was synthesized according to the general procedure and purified by column chromatography (hexane/EtOAc - 3:2). Yellowish foam, yield 65 % (26 mg), *ee* 94 %. The ee was determined by HPLC analysis using Chiralpak IA column (80/20 heptane/i-PrOH, flow rate 1.0 ml/min; λ = 190 nm, 25 °C), t_{minor} = 8.6 min; t_{major} = 12.5 min. **^{1}H NMR** (400 MHz, $CDCl_3$) δ 9.97 (s, 1H), 7.79 – 7.73 (m, 2H), 7.56 – 7.50 (m, 1H), 7.49 – 7.41 (m, 2H), 6.94 (s, 1H), 3.72 (s, 3H), 3.54 (d, *J* = 18.8 Hz, 1H), 3.50 – 3.43 (m, 1H), 3.01 (dt, *J* = 18.9, 1.5 Hz, 1H), 2.19 (d, *J* = 1.3 Hz, 3H), 1.81 – 1.59 (m, 2H), 1.44 – 1.30 (m, 2H), 0.96 – 0.85 (m, 3H). **^{13}C{^{1}H} NMR** (101 MHz, $CDCl_3$) δ 187.5, 174.4, 167.5, 160.4, 137.7, 134.0, 132.1, 128.9 (2C), 127.0 (2C), 65.2, 53.2, 50.9, 49.6, 30.9, 20.9, 14.5, 14.3. **IR** (KBr): ν = 2954, 1738, 1635, 1529, 1321, 1205 cm^{-1}; **$[\alpha]^{25}_{D}$** = +69.1 °, (0.81, $CHCl_3$); **HRMS** (ESI-TOF) m/z: $[M+Na]^+$ Calcd for $C_{19}H_{23}NO_4Na$ 352.1519; Found 352.1520.

Methyl (1*S*,2*R*)-1-benzamido-2-butyl-3-formyl-4-methylcyclopent-3-ene-1-carboxylate (4n)

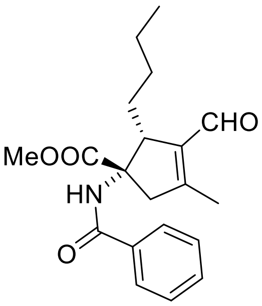

The title compound was synthesized according to the general procedure and purified by column chromatography (hexane/EtOAc - 3:2). Yellowish foam, yield 70 % (29 mg), *ee* 92 %. The ee was determined by HPLC analysis using Chiralpak IA column (80/20 heptane/i-PrOH, flow rate 1.0 ml/min; λ = 190 nm, 25 °C), t_{minoor} = 8.6 min; t_{major} = 12.4 min. **^{1}H NMR** (400 MHz, $CDCl_3$) δ 9.96 (s, 1H), 7.78 – 7.73 (m, 2H), 7.55 – 7.49 (m, 1H), 7.48 – 7.39 (m, 2H), 6.98 (s, 1H), 3.71 (s, 3H), 3.53 (d, *J* = 19.0 Hz, 1H), 3.00 (dt, J_1 = 18.9, J_2 = 1.5 Hz, 1H), 2.18 (q, *J* = 1.4 Hz, 3H), 1.79 – 1.67 (m, 2H), 1.35 – 1.26 (m, 2H), 0.89 – 0.84 (m, 3H). **^{13}C{^{1}H} NMR** (101 MHz, $CDCl_3$) δ 187.5, 174.4, 167.5, 160.2, 137.7, 134.0, 132.0, 128.8 (2C), 127.0 (2C), 65.1, 53.1, 50.9, 49.6, 29.6, 28.3, 23.1, 14.3, 14.0. **IR** (KBr): ν = 2952, 1739, 1635, 1527, 1315, 1203 cm^{-1}; **$[\alpha]^{25}_D$** = +70.9 °, (1.19, $CHCl_3$); **HRMS** (ESI-TOF) m/z: $[M+Na]^+$ Calcd for $C_{20}H_{25}NO_4Na$ 366.1676; Found 366.1677.

Methyl (1*S*,2*R*)-1-(4-bromobenzamido)-3-formyl-4-methyl-2-phenylcyclopent-3-ene-1-carboxylate (4o)

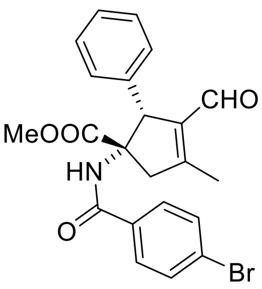

The title compound was synthesized according to the general procedure and purified by column chromatography (hexane/EtOAc - 3:2). Yellowish foam, yield 46 % (26 mg), *ee* 93 %. The ee was determined by HPLC analysis using Chiralpak IB column (70/30 heptane/i-PrOH, flow rate 1.0 ml/min; λ = 190 nm, 25 °C), t_{minor} = 9.2 min; t_{major} = 12.7 min. **^{1}H NMR** (400 MHz, $CDCl_3$) δ 9.98 (s, 1H), 7.48 – 7.35 (m, 5H), 7.22 – 7.17 (m, 2H), 7.12 – 7.04 (m, 2H), 5.98 (s, 1H), 4.52 (s, 1H), 3.93 (d, *J* = 19.3 Hz, 1H), 3.77 (s, 3H), 2.91 (dt, *J* = 19.3, 1.6 Hz, 1H), 2.36 (d, *J* = 1.3 Hz, 3H). **^{13}C{^{1}H} NMR** (101 MHz, $CDCl_3$) δ 186.6, 173.5, 165.7, 162.3, 136.2, 135.9, 132.1, 132.0, 131.9 (2C), 129.6 (2C), 128.9, 128.8, 128.3 (2C), 126.7, 63.9, 57.9, 53.3, 49.9, 14.6. **IR** (KBr): ν = 2951, 1738, 1635, 1481, 1340, 1205 cm^{-1}; **$[\alpha]^{25}_D$** = +53.6 °, (0.69, $CHCl_3$); **HRMS** (ESI-TOF) m/z: $[M+Na]^+$ Calcd for $C_{19}H_{20}NO_4Na$ 464.0468, found 464.0473.

Methyl (1*S*,2*R*)-3-formyl-4-methyl-2-phenyl-1-pivalamidocyclopent-3-ene-1-carboxylate (4s)

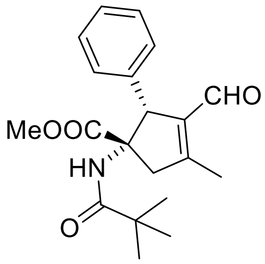

The title compound was synthesized according to the general procedure and purified by column chromatography (hexane/EtOAc - 3:2). Yellowish foam, yield 37 % (15 mg), *ee* 87 %. The ee was determined by HPLC analysis using Chiralpak IA column (80/20 heptane/i-PrOH, flow rate 1.0 ml/min; λ = 190 nm, 25 °C), t_{minor} = 6.2 min; t_{major} = 8.0 min. **^{1}H NMR** (400 MHz, $CDCl_3$) δ 9.98 (s, 1H), 7.45 – 7.31 (m, 3H), 7.15 (d, *J* = 7.2 Hz, 2H), 5.55 (s, 1H), 4.44 (s, 1H), 3.85 (d, *J* = 19.4 Hz, 1H), 3.76 (s, 3H), 2.77 (dt, J_1 = 19.4, J_2 = 1.6 Hz, 1H), 2.34 (s, 3H), 0.86 (s, 9H). **^{13}C{^{1}H} NMR** (101 MHz, $CDCl_3$) δ 186.7, 178.2, 173.9, 162.4, 136.3, 136.1, 129.4 (3C), 128.6 (2C), 63.4, 57.7, 53.1, 50.3, 27.5, 26.9 (3C), 14.5. **IR** (KBr): ν = 2952, 1720, 1653, 1508, 1435, 1201 cm^{-1}; **$[\alpha]^{25}_D$** = -27.3 °, (0.55, $CHCl_3$); **HRMS** (ESI-TOF) m/z: $[M+Na]^+$ Calcd for $C_{20}H_{25}NO_4Na$ 366.1676; Found 366.1675.

4 Further transformations

4.1 Wittig reaction of compound 4a

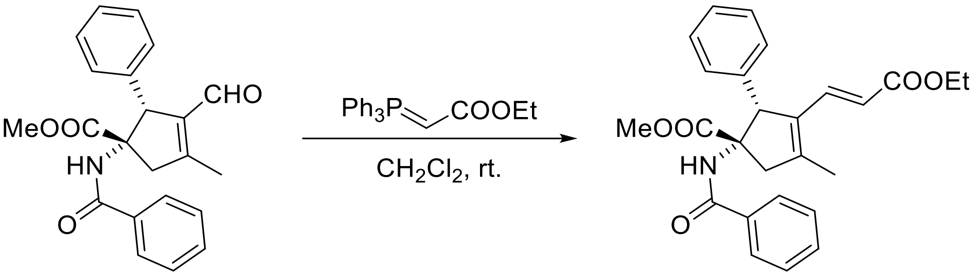

4a **5a**
yield 56 %
ee 96 %

Scheme S1. Preparation of compound **4a**.

The ylide reagent (0.4127 mmol, 144 mg, 5 equiv.) was added to the compound **4a** (0.0826 mmol, 30 mg, 1 equiv.) dissolved in CH_2Cl_2 (2 mL). Reaction mixture was stirred at room temperature until full conversion was reached (TLC monitoring). Product **5a** was isolated after silica separation in Hex/EtOAc (1:1) as white solid foam (20 mg, 56 %).

Methyl (1*S*,2*R*)-1-benzamido-3-((*E*)-3-ethoxy-3-oxoprop-1-en-1-yl)-4-methyl-2-phenylcyclopent-3-ene-1-carboxylate (5a)

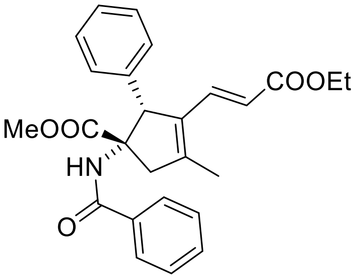

White foam, yield 56 % (20 mg), *ee* 96 %. The ee was determined by HPLC analysis using Chiralpak IA column (70/30 heptane/i-PrOH, flow rate 1.0 ml/min; λ = 190 nm, 25 °C), t_{minor} = 5.8 min; t_{major} = 8.8 min. **^{1}H NMR** (400 MHz, $CDCl_3$) δ 7.57 (d, *J* = 15.8 Hz, 1H), 7.46 – 7.36 (m, 4H), 7.30 – 7.19 (m, 6H), 6.01 (s, 1H), 5.38 (d, *J* = 15.8 Hz, 1H), 4.44 (s, 1H), 4.19 – 4.05 (m, 2H), 3.77 (s, 3H), 3.75 (d, *J* = 18.7 Hz, 1H), 2.78 (dd, J_1 = 18.7, J_2 = 1.7 Hz, 1H), 2.11 (d, *J* = 1.5 Hz, 3H), 1.22 (t, *J* = 7.1 Hz, 3H). **^{13}C{^{1}H} NMR** (101 MHz, $CDCl_3$) δ 174.0, 167.4, 166.6, 149.4, 136.3 (2C), 133.5, 131.8, 131.5, 129.7 (2C), 129.1 (2C), 128.7, 128.6 (2C), 126.8 (2C), 119.1, 64.8, 60.4, 59.3, 53.2, 48.8, 14.8, 14.4. **IR** (KBr): ν = 2947, 1712, 1657, 1522, 1304, 1153 cm^{-1}; **$[\alpha]^{25}_D$** = +69.5 °, (0.77, $CHCl_3$); **HRMS** (ESI-TOF) m/z: $[M+Na]^+$ Calcd for $C_{26}H_{27}NO_5Na$ 456.1781; Found 456.1782.

4.2 Preparation of alcohol 6a

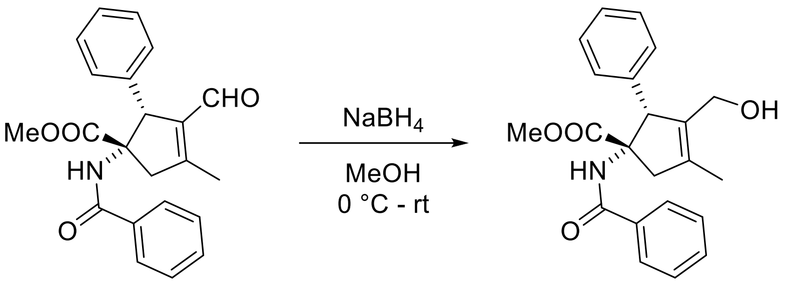

4a

6a
yield 70 %
ee 88 %

Scheme S2. Preparation of compound **6a**.

$NaBH_4$ (0.1652 mmol, 6.3 mg, 2 equiv.) was added to solution of compound **4a** (0.0826 mmol, 30 mg, 1 equiv.) in methanol (1.5 ml) at 0 °C. Reaction mixture was stirred at room temperature until full conversion was reached (TLC monitoring). The reaction mixture was then evaporated, and crude product was purified on silica gel in Hex/EtOAc (1:1). The corresponding alcohol derivative **6a** was obtained as white solid foam(21 mg, 70 %).

Methyl (1*S*,2*R*)-1-benzamido-3-(hydroxymethyl)-4-methyl-2-phenylcyclopent-3-ene-1-carboxylate (6a)

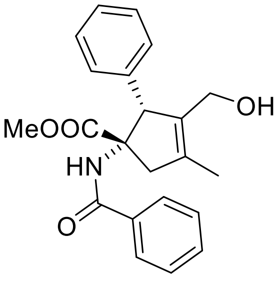

White foam, yield 70 % (21 mg), *ee* 92 %. The ee was determined by HPLC analysis using Chiralpak IA column (70/30 heptane/i-PrOH, flow rate 1.0 ml/min; λ = 250 nm, 25 °C), t_{minor} = 7.4 min; t_{major} = 9.7 min. **^{1}H NMR** (400 MHz, $CDCl_3$) δ 7.46 – 7.33 (m, 4H), 7.30 – 7.21 (m, 6H), 6.03 (s, 1H), 4.49 (s, 1H), 4.33 (d, *J* = 12.5 Hz, 1H), 3.86 (d, *J* = 13.1 Hz, 1H), 3.77 (s, 3H), 3.59 (d, *J* = 17.2 Hz, 1H), 2.71 (d, *J* = 17.4 Hz, 1H), 1.89 (s, 3H). **^{13}C{^{1}H} NMR** (101 MHz, $CDCl_3$) δ 174.5, 166.7, 137.6, 136.8, 133.7, 133.5, 131.7, 129.7 (2C), 129.4 (2C), 128.5 (3C), 126.8 (2C), 64.7, 60.3, 57.8, 53.1, 48.5, 14.0. **IR** (KBr): ν = 2924, 1732, 1637, 1525, 1248 cm^{-1}; **[α]$^{25}_D$** = -6.0 °, (0.67, $CHCl_3$); **HRMS** (ESI-TOF) m/z: $[M+Na]^+$ Calcd for $C_{22}H_{23}NO_4Na$ 388.1519, found 388.1523.

5 Crystalographic data for 4g

X-ray single crystal data for **4g** were collected on Bruker D8 VENTURE Kappa Duo PHOTONIII by IµS micro-focus sealed tube MoKα (λ= 0.71073 Å). The structure was solved by direct methods (XT)[3] and refined by full matrix least squares based on F^2 (SHELXL2019)[4]. The hydrogen atoms on carbon were fixed into idealized positions (riding model) and assigned temperature factors either $H_{iso}(H) = 1.2$ U_{eq}(pivot atom) or $H_{iso}(H) = 1.5$ U_{eq} (pivot atom) for methyl moiety. The absolute structure[5] determination was based on anomalous dispersion of Br atom.

Crystal data for **4g,** $C_{22}H_{20}BrNO_4$, $M_r = 442.30$; Orthorhombic, $P2_12_12_1$ (No 19), $a = 8.6353$ (2) Å, b = 13.5179 (3) Å, $c = 16.8326$ (4) Å, $V = 1964.89$ (8) Å^3, Z = 4, $D_x = 1.495$ Mg m^{-3}, temperature of sample 120(2) K, colorless prism of dimensions 0.17 × 0.09 × 0.09 mm, multi-scan absorption correction (μ = 2.12 mm^{-1}) $T_{min} = 0.70$, $T_{max} = 0.84$; a total of 27927 measured reflections (θ_{max}= 30˚), from which 5727 were unique ($R_{int} = 0.029$) and 5262 observed according to the $I > 2\sigma(I)$ criterion. The refinement converged (Δ/σ_{max}= 0.001) to $R = 0.027$ for observed reflections and $wR(F^2) = 0.068$, $GOF = 1.04$ for 255 parameters and all 5727 reflections. The final difference map displayed no peaks of chemical significance ($\Delta\rho_{max} = 0.61$, $\Delta\rho_{min}$ -0.55 e.Å^{-3}).

Absolute structure parameter: -0.003 (2).

X-ray crystallographic data have been deposited with the Cambridge Crystallographic Data Centre under deposition number CCDC 2356173 for **4g** and can be obtained free of charge from the Centre via its website (https://www.ccdc.cam.ac.uk/structures/).

[3] SHELXT: Sheldrick, G.M. (2015). *Acta Cryst*. **A71**, 3-8.
[4] SHELXL: Sheldrick, G.M. (2015). *Acta Cryst.* **C71**, 3-8.
[5] Parsons,S., Flack, H.D. and Wagner, T. (2013) *Acta Cryst*. **B69**, 249-259.

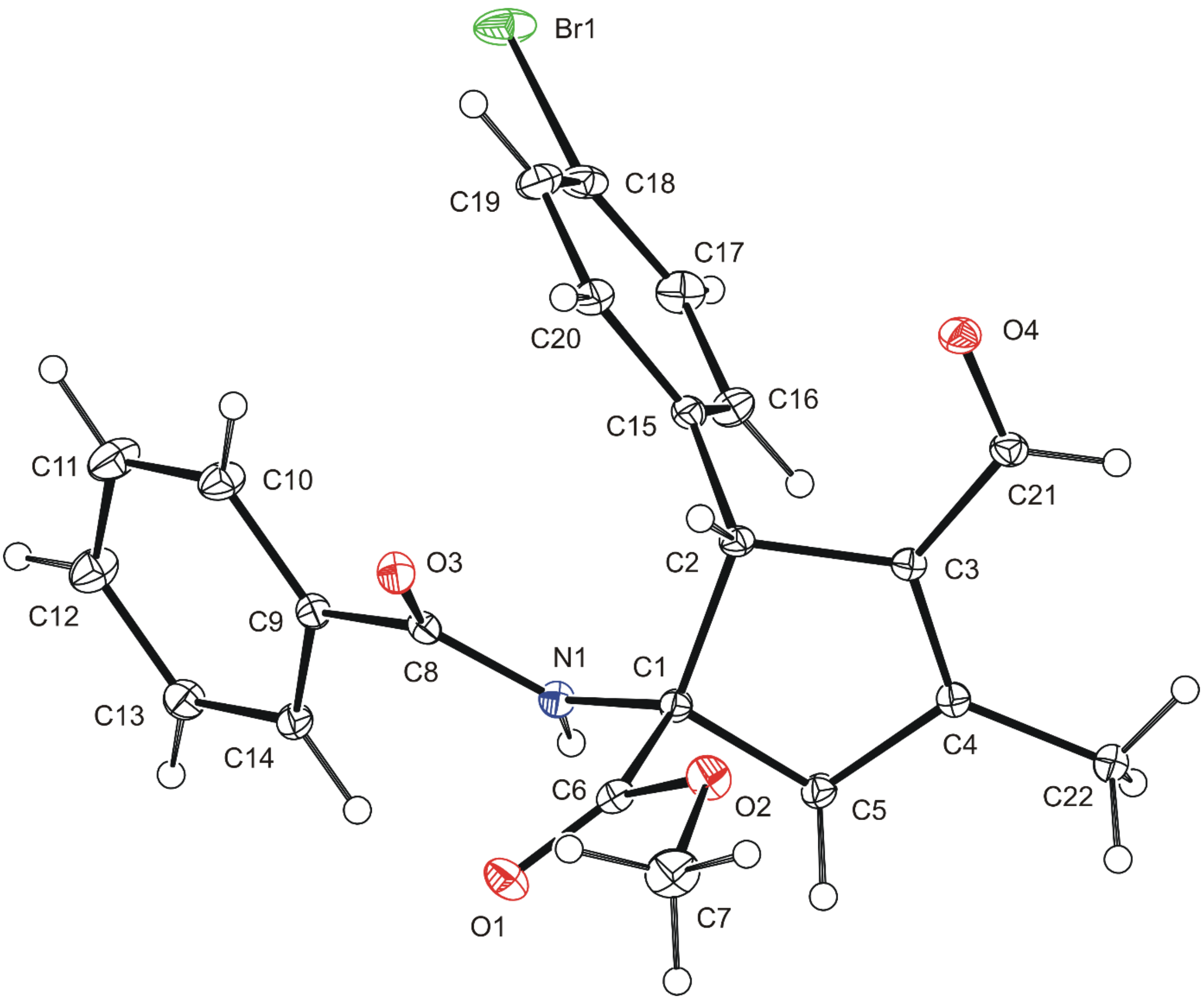

Figure S2. View on molecule of **4g** with atom numbering schema, the displacement ellipsoids are drawn on 30% probability level. The chirality descriptors for C1 and C2 are *S* and *R*, respectively.

Table S4. Crystal data.

Crystal data	
Chemical formula	$C_{22}H_{20}BrNO_4$
M_r	442.30
Crystal system, space group	Orthorhombic, $P2_12_12_1$
Temperature (K)	120
a, *b*, *c* (Å)	8.6353 (2), 13.5179 (3), 16.8326 (4)
V (Å^3)	1964.89 (8)
Z	4
Radiation type	Mo *K*a
m (mm^{-1})	2.12
Crystal size (mm)	0.17 × 0.09 × 0.09

Data collection	
Diffractometer	Bruker D8 VENTURE Kappa Duo PHOTONIII CMOS
Absorption correction	Multi-scan<br>*SADABS2016*/2 - Bruker AXS area detector scaling and absorption correction
T_{min}, T_{max}	0.70, 0.84
No. of measured, independent and observed [I > 2s(I)] reflections	27927, 5727, 5262
R_{int}	0.029
(sin q/l)$_{max}$ (Å^{-1})	0.703
Refinement	
$R[F^2 > 2s(F^2)]$, $wR(F^2)$, S	0.027, 0.068, 1.04
No. of reflections	5727
No. of parameters	255
H-atom treatment	H-atom parameters constrained
Dρ$_{max}$, Dρ$_{min}$ (e Å^{-3})	0.61, -0.55
Absolute structure	Flack x determined using 2120 quotients [(I+)-(I-)]/[(I+)+(I-)] (Parsons, Flack and Wagner, Acta Cryst. B69 (2013) 249-259).
Absolute structure parameter	-0.003 (2)

Computer programs: Bruker Instrument Service vV6.2.14, *SAINT* V8.40A (Bruker Nano, Inc., 2019), SHELXT 2014/5 (Sheldrick, 2014), *SHELXL2019*/2 (Sheldrick, 2019).

Table S5. Hydrogen-bond geometry (Å, º) for (**4g**).

D—H···*A*	*D*—H	H···*A*	*D*···*A*	*D*—H···*A*
N1—H1···O4^{i}	0.88	2.14	3.007 (2)	168

Symmetry code: (i) -*x*+1, *y*-1/2, -*z*+3/2.

References

NOT FOUND

Document origin: *publCIF* [Westrip, S. P. (2010). *J. Apply. Cryst.*, **43**, 920-925].

Computing details

Data collection: Bruker Instrument Service vV6.2.14; cell refinement: *SAINT* V8.40A (Bruker Nano, Inc., 2019); data reduction: *SAINT* V8.40A (Bruker Nano, Inc., 2019); program(s) used to solve structure: SHELXT 2014/5 (Sheldrick, 2014); program(s) used to refine structure: *SHELXL2019*/2 (Sheldrick, 2019).

(4g)

Special details

Geometry. All esds (except the esd in the dihedral angle between two l.s. planes) are estimated using the full covariance matrix. The cell esds are taken into account individually in the estimation of esds in distances, angles and torsion angles; correlations between esds in cell parameters are only used when they are defined by crystal symmetry. An approximate (isotropic) treatment of cell esds is used for estimating esds involving l.s. planes.

Table S6. Fractional atomic coordinates and isotropic or equivalent isotropic displacement parameters (Å^2) for (**4g**).

	x	*y*	*z*	U_{iso}*/U_{eq}
Br1	0.26927 (4)	0.68087 (2)	0.42504 (2)	0.03263 (9)
O1	0.1531 (2)	0.58184 (14)	0.93276 (11)	0.0255 (4)
O2	0.3609 (2)	0.68058 (14)	0.94520 (10)	0.0231 (3)
O3	0.07992 (19)	0.66362 (12)	0.77369 (11)	0.0195 (3)
O4	0.6638 (2)	0.82369 (13)	0.76146 (10)	0.0224 (3)
N1	0.2364 (2)	0.53027 (12)	0.78048 (11)	0.0158 (3)

H1	0.251166	0.467618	0.768492	0.019*
C1	0.3492 (2)	0.58293 (16)	0.82846 (13)	0.0146 (4)
C2	0.4253 (2)	0.67490 (18)	0.78405 (13)	0.0146 (4)
H2	0.385314	0.737436	0.808286	0.018*
C3	0.5953 (2)	0.66378 (15)	0.80432 (13)	0.0145 (4)
C4	0.6291 (3)	0.57710 (16)	0.84016 (13)	0.0148 (4)
C5	0.4873 (3)	0.51447 (16)	0.85001 (14)	0.0173 (4)
H5A	0.478239	0.490671	0.905434	0.021*
H5B	0.491014	0.456579	0.814038	0.021*
C6	0.2716 (3)	0.61453 (16)	0.90678 (12)	0.0177 (4)
C7	0.3074 (4)	0.7060 (2)	1.02417 (16)	0.0312 (6)
H7A	0.379522	0.753171	1.048369	0.047*
H7B	0.302335	0.646059	1.056834	0.047*
H7C	0.204279	0.735893	1.020726	0.047*
C8	0.1093 (3)	0.57757 (16)	0.75448 (14)	0.0154 (4)
C9	0.0062 (3)	0.52249 (16)	0.69859 (14)	0.0160 (4)
C10	-0.0722 (3)	0.57794 (18)	0.64147 (16)	0.0240 (5)
H10	-0.056562	0.647415	0.638689	0.029*
C11	-0.1721 (3)	0.5327 (2)	0.58904 (17)	0.0297 (6)
H11	-0.223499	0.570797	0.549605	0.036*
C12	-0.1980 (3)	0.43126 (19)	0.59377 (15)	0.0271 (6)
H12	-0.268718	0.400345	0.558391	0.033*
C13	-0.1206 (3)	0.37557 (17)	0.65002 (15)	0.0213 (5)
H13	-0.137678	0.306236	0.652839	0.026*
C14	-0.0179 (3)	0.42037 (17)	0.70252 (14)	0.0183 (4)
H14	0.035639	0.381758	0.740862	0.022*
C15	0.3961 (2)	0.67732 (18)	0.69554 (13)	0.0162 (4)
C16	0.4490 (3)	0.60282 (18)	0.64497 (14)	0.0207 (5)
H16	0.510214	0.550667	0.666034	0.025*
C17	0.4137 (3)	0.60338 (18)	0.56407 (15)	0.0245 (5)

H17	0.449888	0.552214	0.530139	0.029*
C18	0.3250 (3)	0.6799 (2)	0.53442 (13)	0.0230 (5)
C19	0.2724 (3)	0.75613 (17)	0.58248 (15)	0.0245 (5)
H19	0.212490	0.808612	0.560938	0.029*
C20	0.3091 (3)	0.75413 (17)	0.66271 (14)	0.0203 (5)
H20	0.274211	0.806247	0.696065	0.024*
C21	0.7047 (3)	0.74251 (16)	0.78702 (13)	0.0178 (4)
H21	0.811855	0.730895	0.795760	0.021*
C22	0.7813 (3)	0.54026 (17)	0.86946 (14)	0.0187 (4)
H22A	0.775311	0.528648	0.926851	0.028*
H22B	0.861539	0.589653	0.858426	0.028*
H22C	0.807200	0.478244	0.842395	0.028*

Table S7. Atomic displacement parameters (Å^2) for (**4g**).

	U^{11}	U^{22}	U^{33}	U^{12}	U^{13}	U^{23}
Br1	0.05424 (18)	0.02583 (12)	0.01783 (11)	-0.01489 (12)	-0.01132 (12)	0.00432 (10)
O1	0.0237 (8)	0.0293 (9)	0.0236 (9)	-0.0042 (7)	0.0069 (8)	0.0013 (7)
O2	0.0257 (8)	0.0278 (8)	0.0158 (8)	-0.0027 (8)	0.0013 (6)	-0.0056 (7)
O3	0.0183 (8)	0.0133 (8)	0.0271 (9)	0.0002 (6)	0.0001 (6)	-0.0019 (6)
O4	0.0241 (8)	0.0174 (7)	0.0257 (9)	-0.0045 (7)	-0.0033 (6)	0.0050 (7)
N1	0.0169 (9)	0.0112 (7)	0.0192 (8)	-0.0010 (7)	-0.0008 (7)	-0.0017 (6)
C1	0.0143 (9)	0.0139 (9)	0.0157 (10)	-0.0002 (8)	-0.0007 (8)	0.0005 (7)
C2	0.0171 (9)	0.0116 (8)	0.0153 (10)	-0.0006 (8)	-0.0008 (7)	0.0012 (8)
C3	0.0165 (9)	0.0139 (10)	0.0131 (9)	-0.0009 (7)	-0.0001 (7)	-0.0005 (7)
C4	0.0151 (9)	0.0159 (9)	0.0133 (10)	0.0002 (8)	0.0008 (8)	-0.0020 (8)
C5	0.0167 (10)	0.0143 (9)	0.0209 (11)	0.0002 (8)	-0.0020 (8)	0.0024 (8)
C6	0.0194 (11)	0.0174 (9)	0.0162 (10)	0.0022 (8)	-0.0017 (8)	0.0021 (7)
C7	0.0398 (16)	0.0371 (14)	0.0169 (12)	0.0020 (11)	0.0001 (11)	-0.0062 (10)

C8	0.0148 (10)	0.0146 (9)	0.0169 (10)	-0.0019 (8)	0.0026 (8)	0.0008 (8)
C9	0.0154 (10)	0.0143 (9)	0.0183 (11)	0.0001 (8)	0.0008 (8)	-0.0003 (8)
C10	0.0306 (13)	0.0162 (10)	0.0253 (13)	-0.0004 (9)	-0.0049 (10)	0.0026 (9)
C11	0.0387 (15)	0.0241 (12)	0.0264 (14)	-0.0007 (11)	-0.0122 (11)	0.0034 (10)
C12	0.0339 (14)	0.0223 (11)	0.0252 (13)	-0.0009 (10)	-0.0094 (10)	-0.0015 (9)
C13	0.0233 (11)	0.0162 (10)	0.0245 (12)	-0.0028 (9)	-0.0012 (9)	-0.0005 (9)
C14	0.0173 (10)	0.0154 (10)	0.0222 (11)	0.0009 (8)	-0.0009 (9)	0.0022 (8)
C15	0.0161 (9)	0.0155 (9)	0.0171 (10)	-0.0033 (9)	-0.0011 (7)	0.0016 (9)
C16	0.0254 (12)	0.0191 (11)	0.0177 (11)	0.0027 (9)	-0.0026 (9)	-0.0008 (9)
C17	0.0324 (13)	0.0225 (11)	0.0187 (12)	-0.0024 (10)	-0.0012 (10)	-0.0036 (9)
C18	0.0321 (12)	0.0230 (10)	0.0139 (10)	-0.0100 (11)	-0.0040 (8)	0.0028 (9)
C19	0.0302 (12)	0.0211 (10)	0.0222 (11)	-0.0022 (9)	-0.0068 (11)	0.0062 (9)
C20	0.0236 (11)	0.0174 (9)	0.0199 (11)	0.0004 (9)	-0.0013 (9)	0.0020 (8)
C21	0.0196 (11)	0.0191 (10)	0.0146 (10)	-0.0027 (8)	-0.0006 (8)	0.0009 (8)
C22	0.0162 (10)	0.0201 (10)	0.0197 (10)	0.0014 (9)	-0.0005 (9)	0.0011 (8)

Table S8. Geometric parameters (Å, º) for (**4g**).

Br1—C18	1.903 (2)	C9—C10	1.394 (3)
O1—C6	1.197 (3)	C9—C14	1.398 (3)
O2—C6	1.345 (3)	C10—C11	1.378 (4)
O2—C7	1.448 (3)	C10—H10	0.9500
O3—C8	1.234 (3)	C11—C12	1.391 (4)
O4—C21	1.231 (3)	C11—H11	0.9500
N1—C8	1.343 (3)	C12—C13	1.382 (4)
N1—C1	1.452 (3)	C12—H12	0.9500
N1—H1	0.8800	C13—C14	1.391 (3)
C1—C6	1.540 (3)	C13—H13	0.9500
C1—C5	1.552 (3)	C14—H14	0.9500
C1—C2	1.592 (3)	C15—C16	1.395 (3)
C2—C15	1.511 (3)	C15—C20	1.396 (3)
C2—C3	1.515 (3)	C16—C17	1.395 (3)
C2—H2	1.0000	C16—H16	0.9500
C3—C4	1.350 (3)	C17—C18	1.380 (4)
C3—C21	1.453 (3)	C17—H17	0.9500
C4—C22	1.490 (3)	C18—C19	1.387 (4)
C4—C5	1.497 (3)	C19—C20	1.387 (3)
C5—H5A	0.9900	C19—H19	0.9500
C5—H5B	0.9900	C20—H20	0.9500
C7—H7A	0.9800	C21—H21	0.9500
C7—H7B	0.9800	C22—H22A	0.9800
C7—H7C	0.9800	C22—H22B	0.9800
C8—C9	1.494 (3)	C22—H22C	0.9800
C6—O2—C7	114.6 (2)	C11—C10—C9	120.5 (2)
C8—N1—C1	119.74 (18)	C11—C10—H10	119.8
C8—N1—H1	120.1	C9—C10—H10	119.8

C1—N1—H1	120.1	C10—C11—C12	120.1 (2)
N1—C1—C6	108.66 (18)	C10—C11—H11	120.0
N1—C1—C5	110.69 (18)	C12—C11—H11	120.0
C6—C1—C5	107.45 (18)	C13—C12—C11	119.9 (2)
N1—C1—C2	113.48 (17)	C13—C12—H12	120.0
C6—C1—C2	111.41 (17)	C11—C12—H12	120.0
C5—C1—C2	104.98 (17)	C12—C13—C14	120.4 (2)
C15—C2—C3	112.68 (18)	C12—C13—H13	119.8
C15—C2—C1	114.27 (18)	C14—C13—H13	119.8
C3—C2—C1	102.50 (17)	C13—C14—C9	119.7 (2)
C15—C2—H2	109.0	C13—C14—H14	120.2
C3—C2—H2	109.0	C9—C14—H14	120.2
C1—C2—H2	109.0	C16—C15—C20	118.1 (2)
C4—C3—C21	125.8 (2)	C16—C15—C2	122.1 (2)
C4—C3—C2	113.34 (19)	C20—C15—C2	119.7 (2)
C21—C3—C2	120.84 (19)	C17—C16—C15	121.3 (2)
C3—C4—C22	129.0 (2)	C17—C16—H16	119.3
C3—C4—C5	111.32 (19)	C15—C16—H16	119.3
C22—C4—C5	119.71 (19)	C18—C17—C16	118.6 (2)
C4—C5—C1	105.37 (17)	C18—C17—H17	120.7
C4—C5—H5A	110.7	C16—C17—H17	120.7
C1—C5—H5A	110.7	C17—C18—C19	121.8 (2)
C4—C5—H5B	110.7	C17—C18—Br1	119.7 (2)
C1—C5—H5B	110.7	C19—C18—Br1	118.47 (19)
H5A—C5—H5B	108.8	C18—C19—C20	118.6 (2)
O1—C6—O2	124.0 (2)	C18—C19—H19	120.7
O1—C6—C1	125.6 (2)	C20—C19—H19	120.7
O2—C6—C1	110.24 (19)	C19—C20—C15	121.5 (2)
O2—C7—H7A	109.5	C19—C20—H20	119.2
O2—C7—H7B	109.5	C15—C20—H20	119.2

H7A—C7—H7B	109.5	O4—C21—C3	122.4 (2)
O2—C7—H7C	109.5	O4—C21—H21	118.8
H7A—C7—H7C	109.5	C3—C21—H21	118.8
H7B—C7—H7C	109.5	C4—C22—H22A	109.5
O3—C8—N1	122.1 (2)	C4—C22—H22B	109.5
O3—C8—C9	120.8 (2)	H22A—C22—H22B	109.5
N1—C8—C9	117.05 (19)	C4—C22—H22C	109.5
C10—C9—C14	119.4 (2)	H22A—C22—H22C	109.5
C10—C9—C8	117.1 (2)	H22B—C22—H22C	109.5
C14—C9—C8	123.4 (2)		

Symmetry code: (i) -*x*+1, *y*-1/2, -*z*+3/2.

Document origin: *publCIF* [Westrip, S. P. (2010). *J. Apply. Cryst.*, **43**, 920-925].

6 NMR spectra

^{1}H NMR (400 MHz, $CDCl_3$)

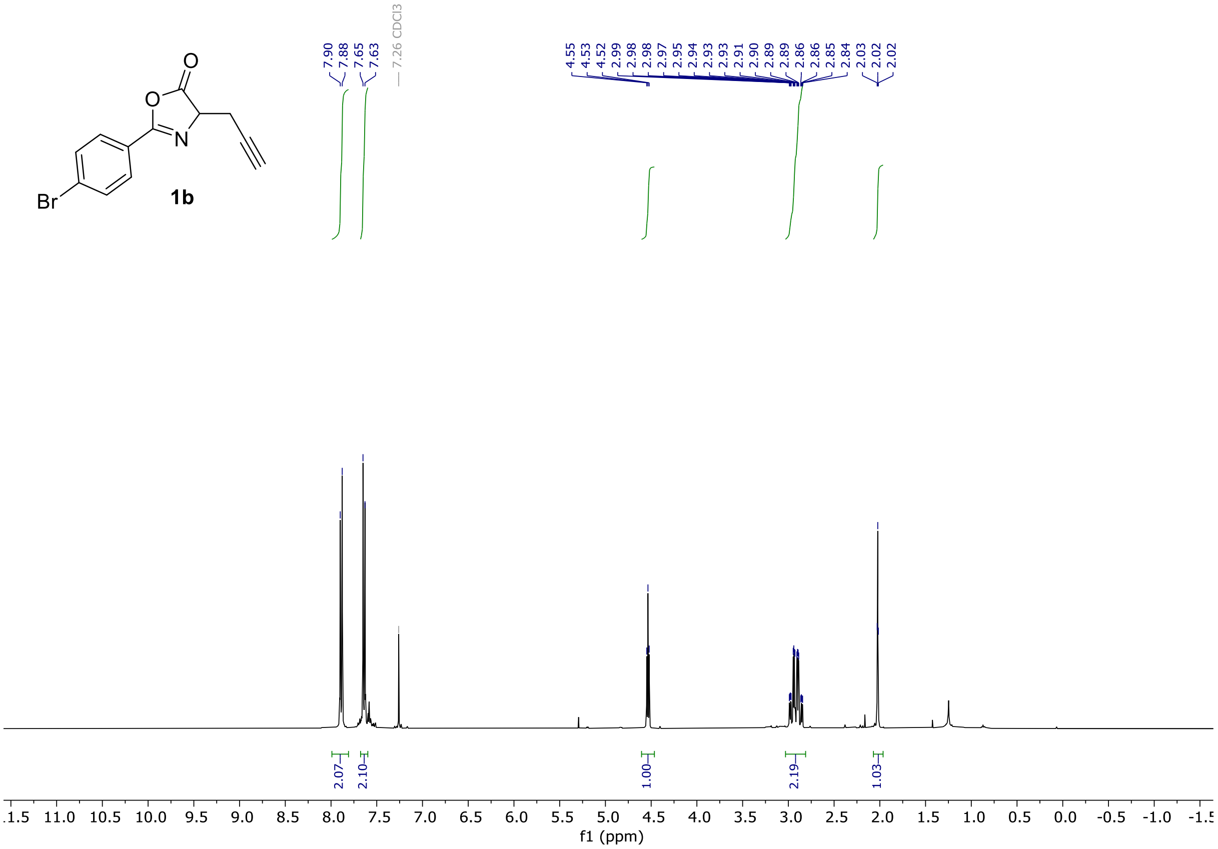

^{13}C{^{1}H} NMR (101 MHz, $CDCl_3$)

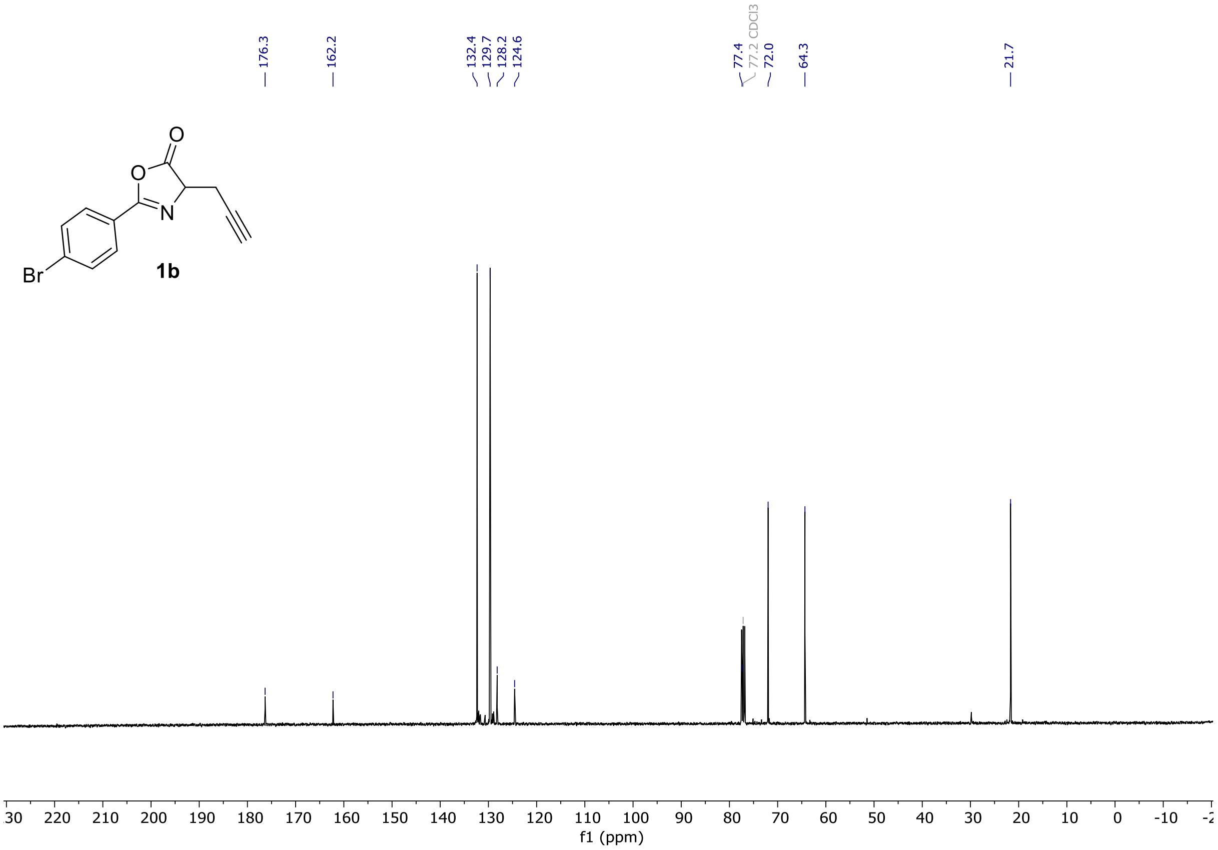

^{1}**H NMR** (400 MHz, $CDCl_3$)

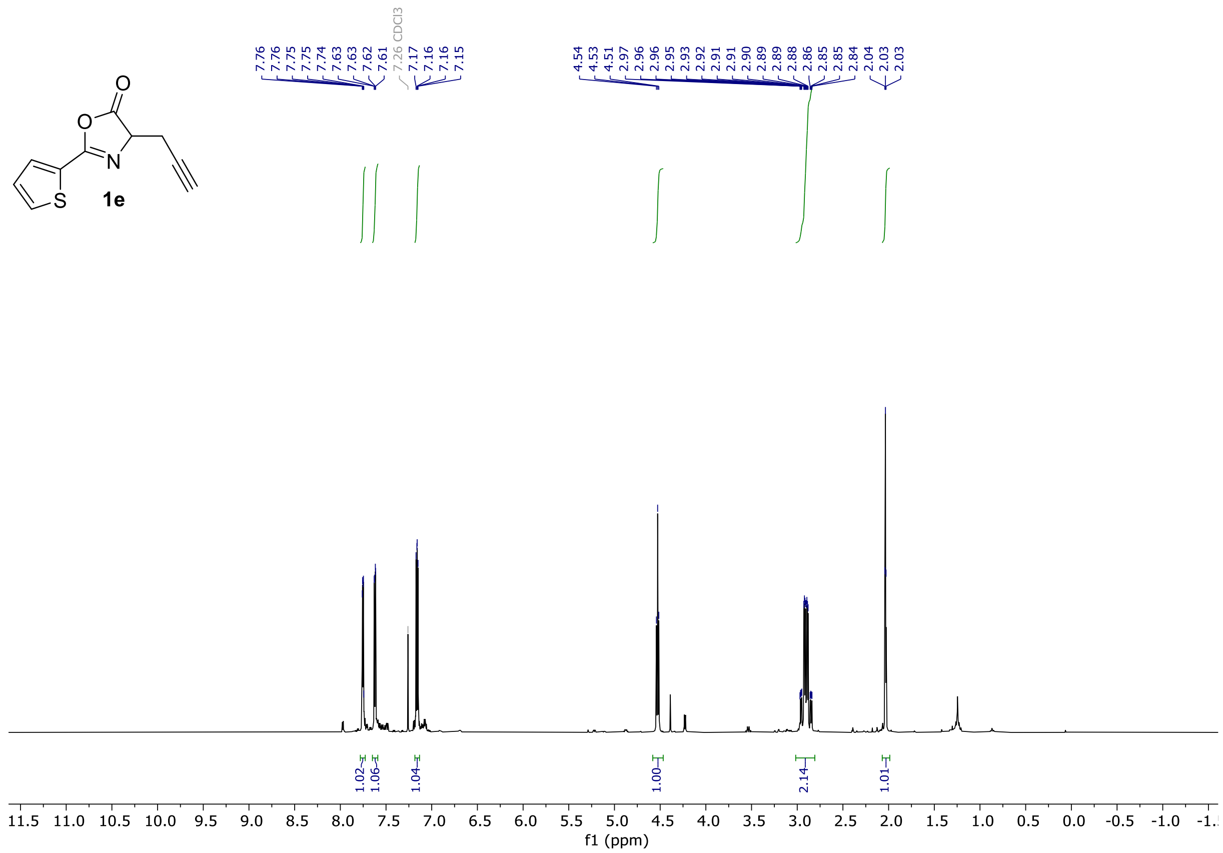

^{13}**C{^{1}H} NMR** (101 MHz, $CDCl_3$)

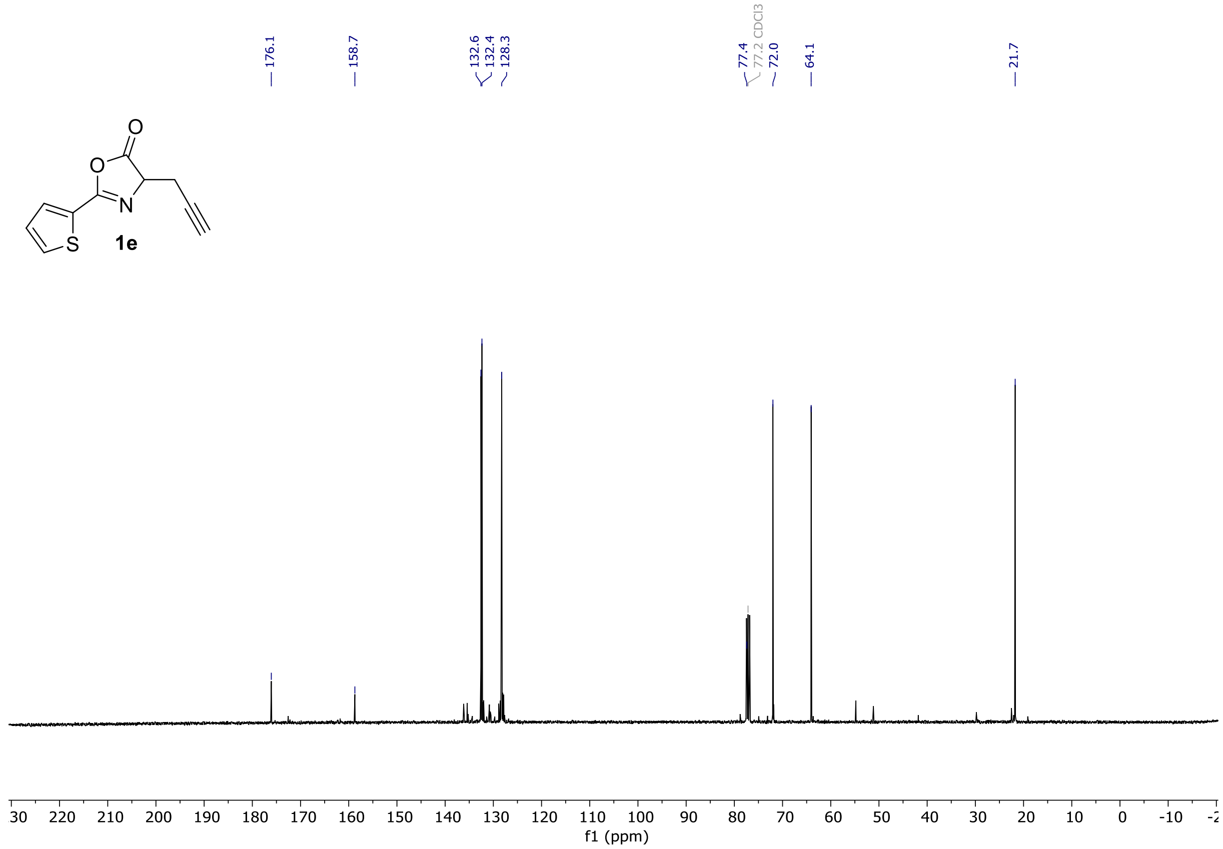

^{1}H NMR (400 MHz, $CDCl_3$)

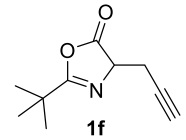

1f

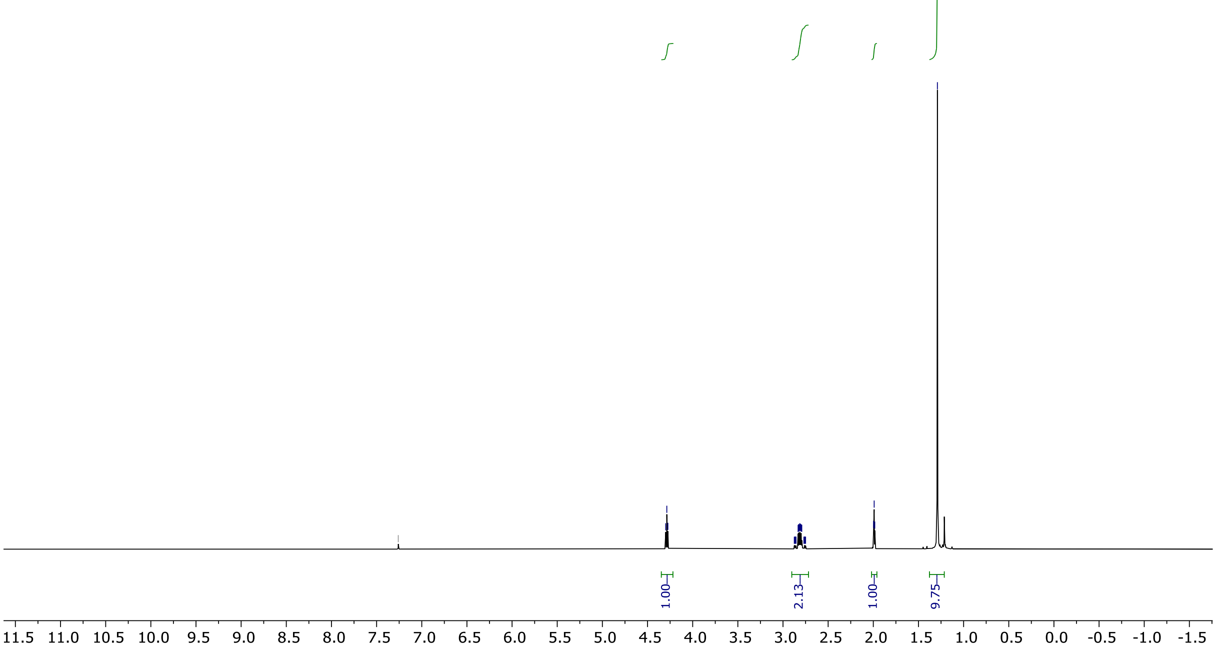

^{13}C{^{1}H} NMR (101 MHz, $CDCl_3$)

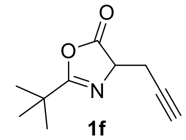

1f

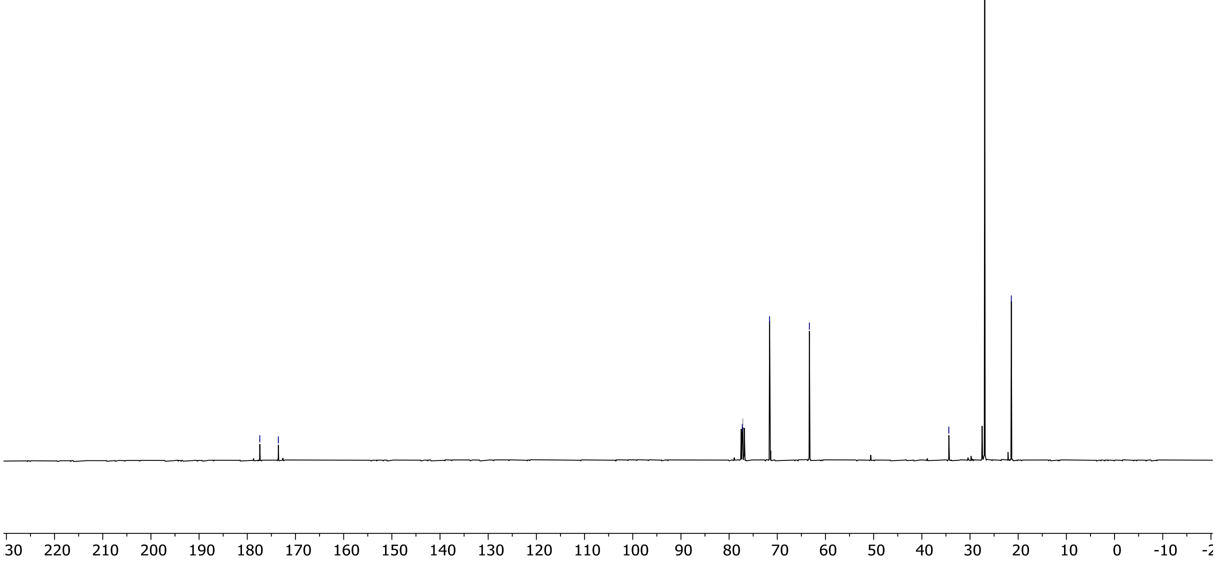

^{1}**H NMR** (400 MHz, $CDCl_3$)

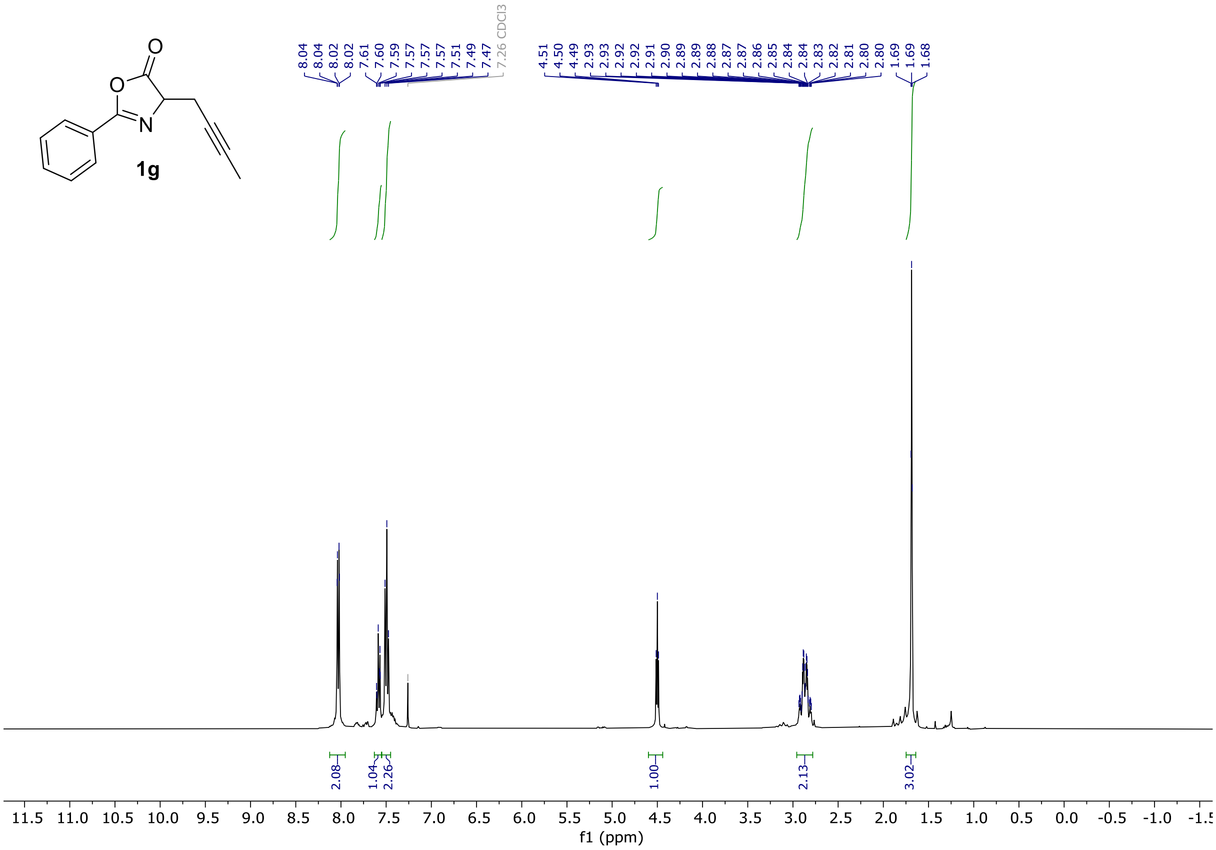

^{13}**C{^{1}H} NMR** (101 MHz, $CDCl_3$)

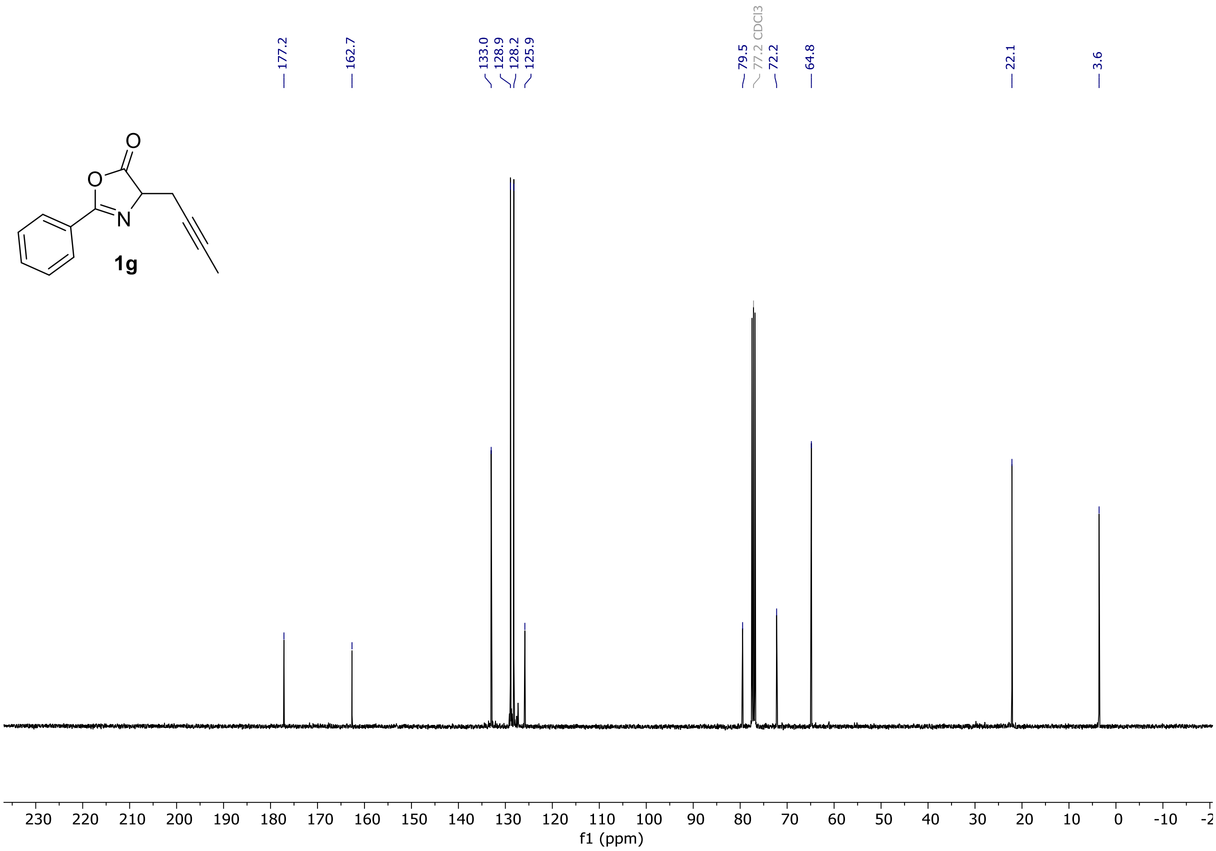

^{1}H NMR (400 MHz, $CDCl_3$)

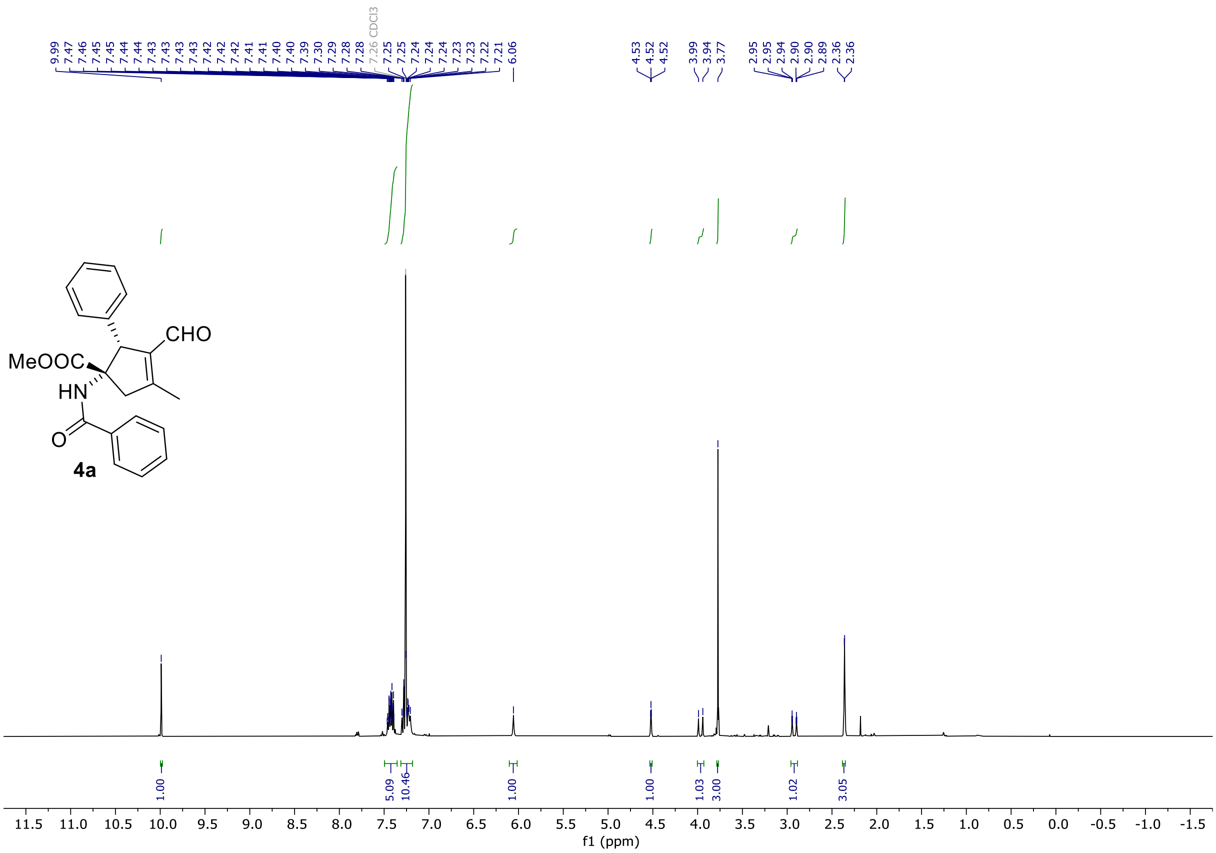

^{13}C{^{1}H} NMR (101 MHz, $CDCl_3$)

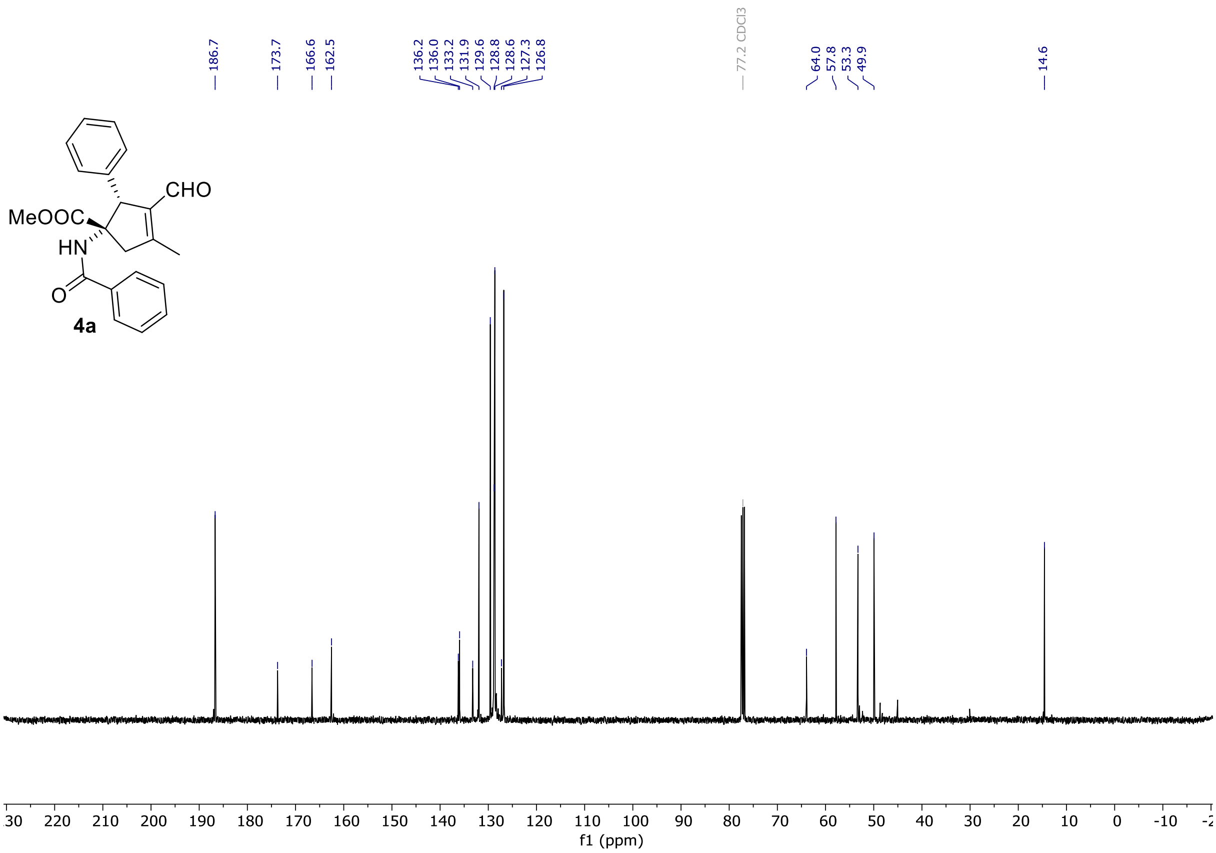

^{1}H NMR (400 MHz, $CDCl_3$)

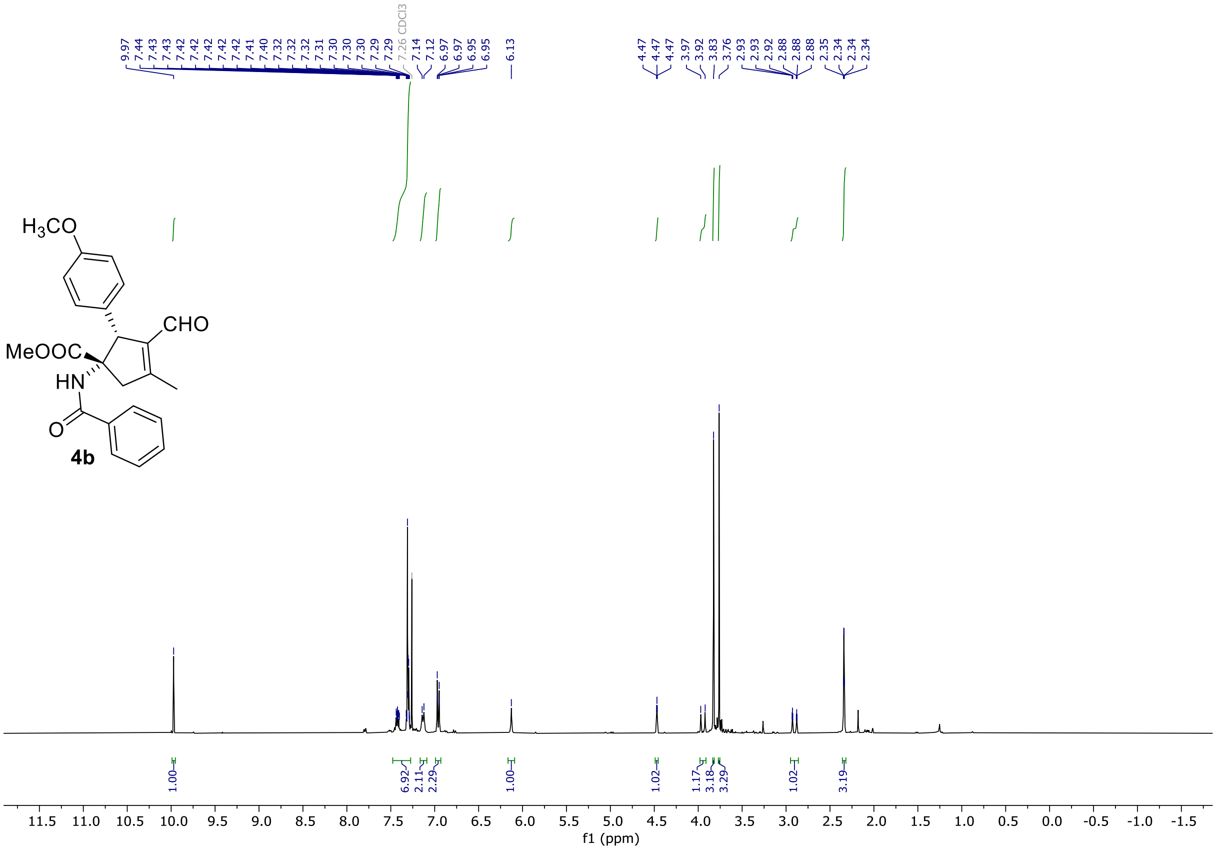

^{13}C{^{1}H} NMR (101 MHz, $CDCl_3$)

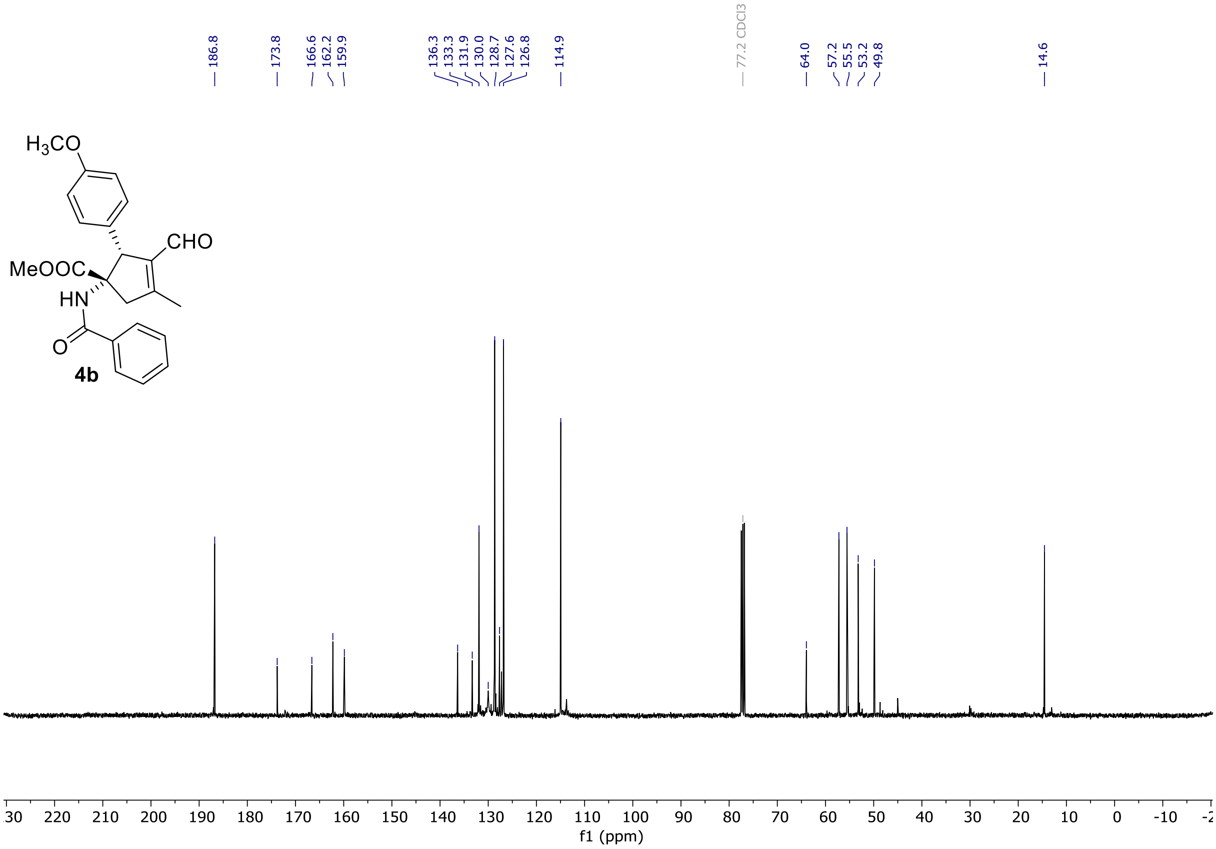

^{1}H NMR (400 MHz, $CDCl_3$)

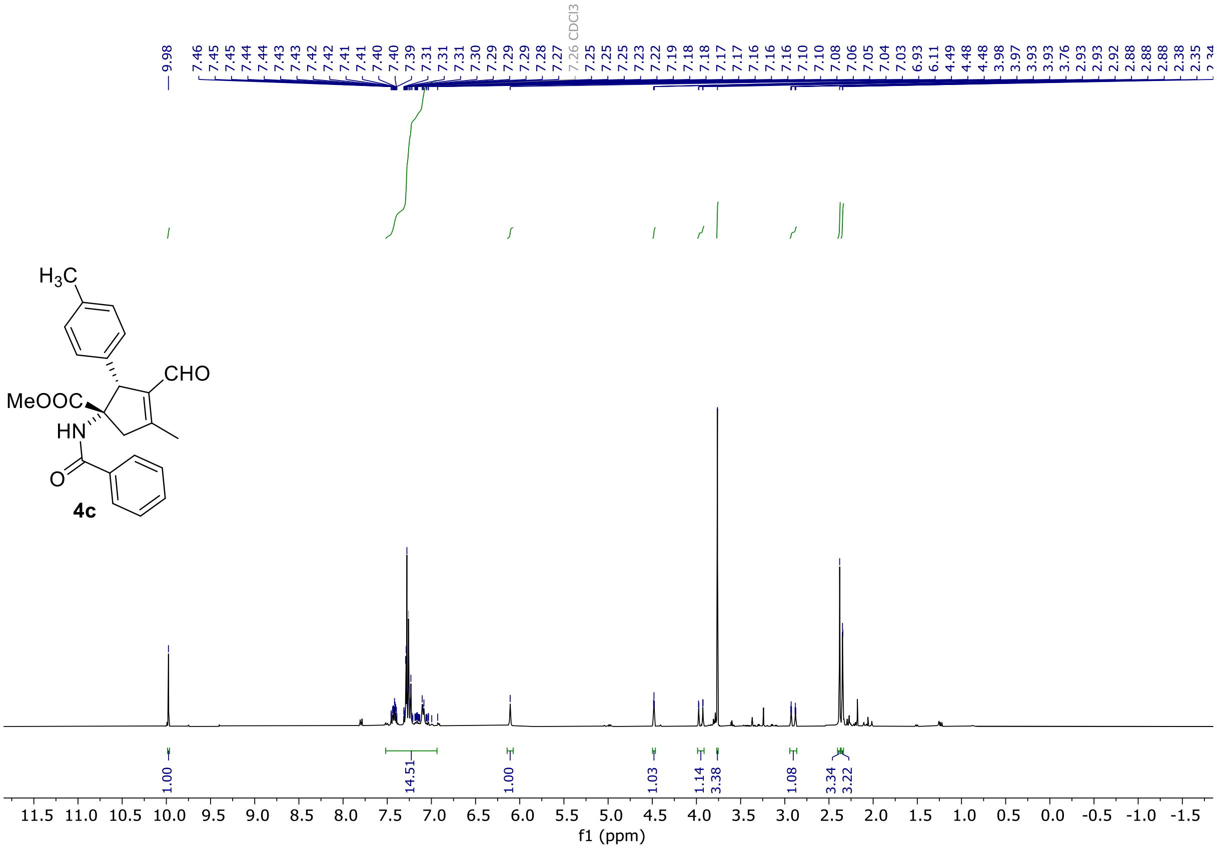

^{13}C{^{1}H} NMR (101 MHz, $CDCl_3$)

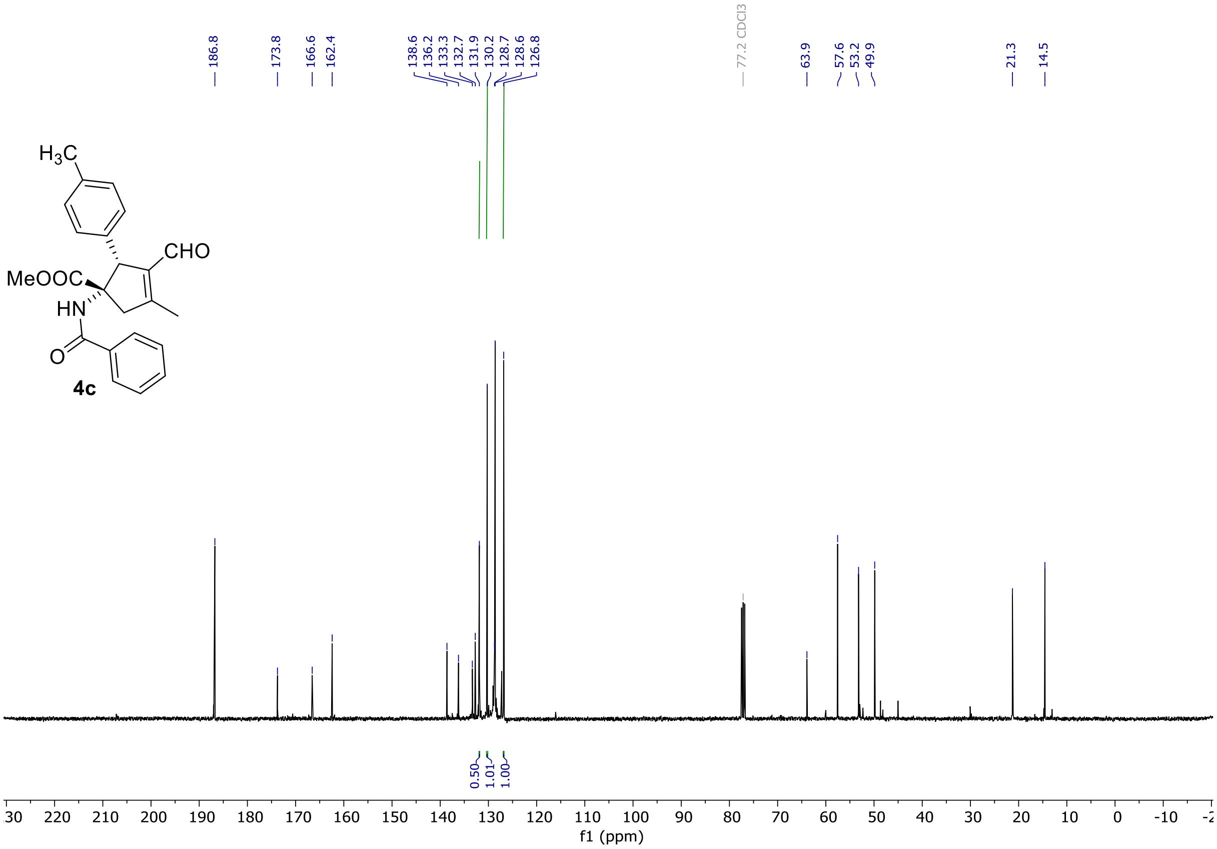

^{1}H NMR (400 MHz, $CDCl_3$)

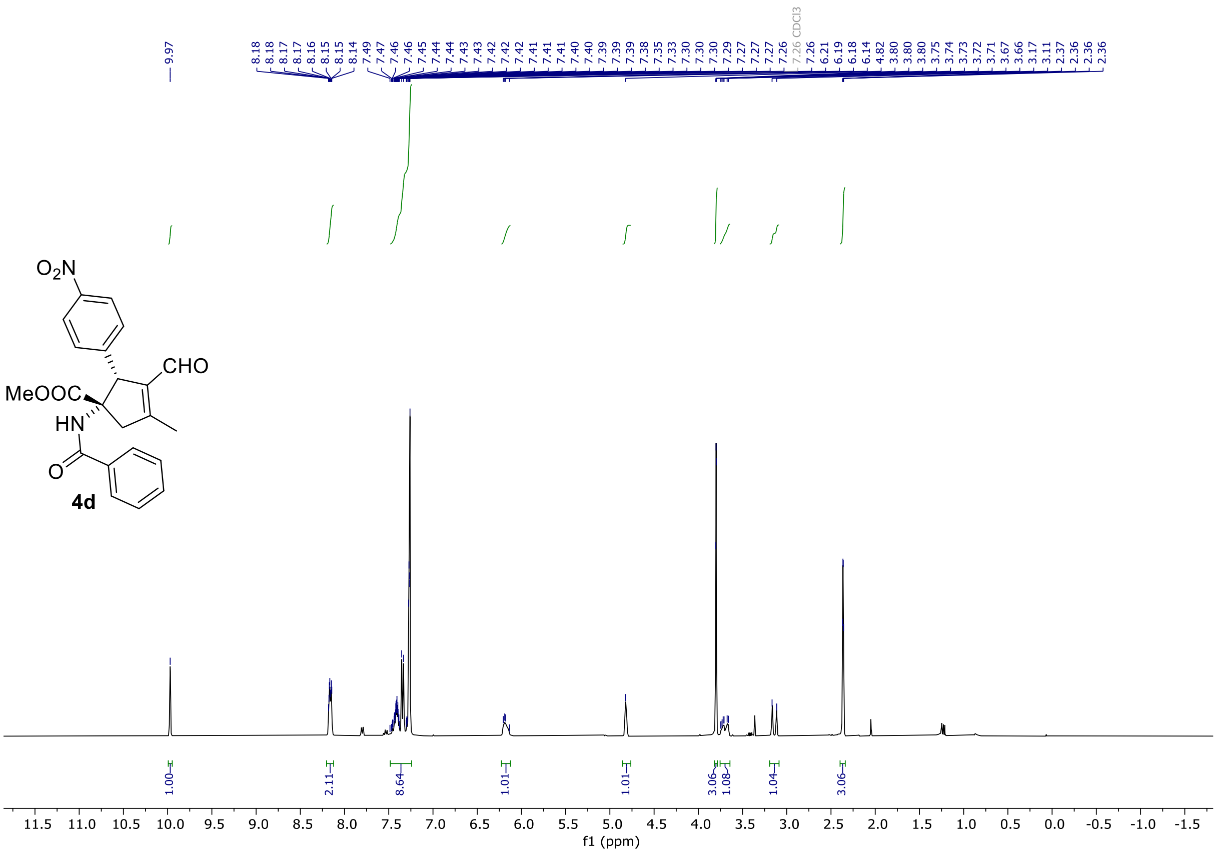

$^{13}C\{^1H\}$ NMR (101 MHz, $CDCl_3$)

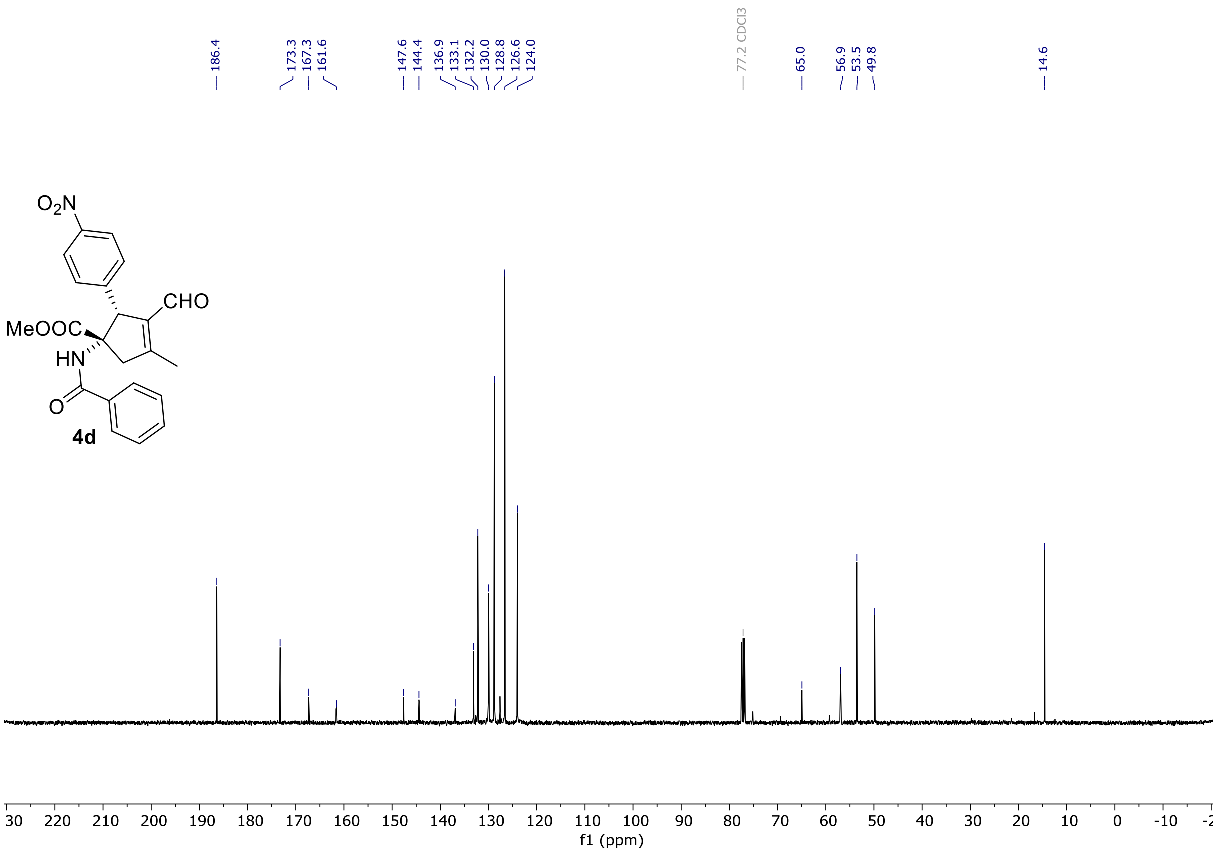

^{1}H NMR (400 MHz, $CDCl_3$)

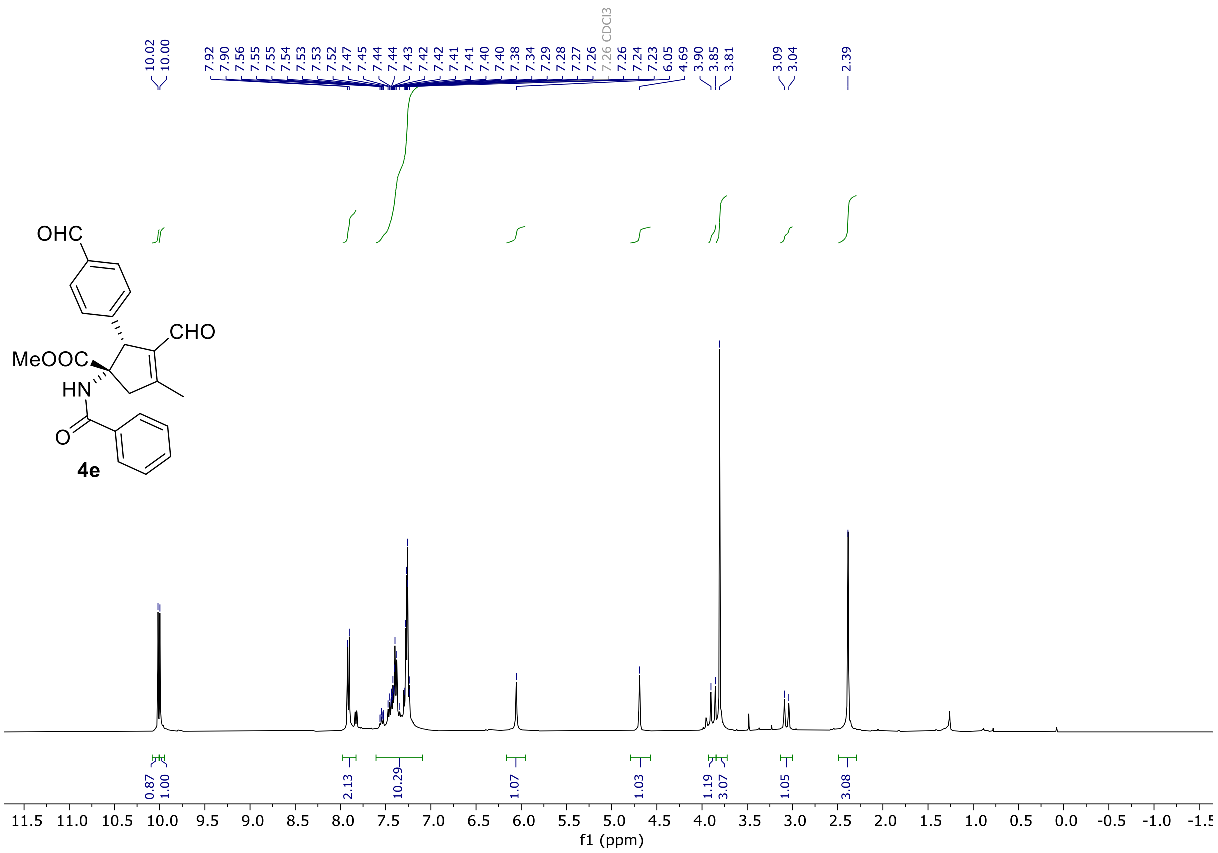

^{13}C{^{1}H} NMR (101 MHz, $CDCl_3$)

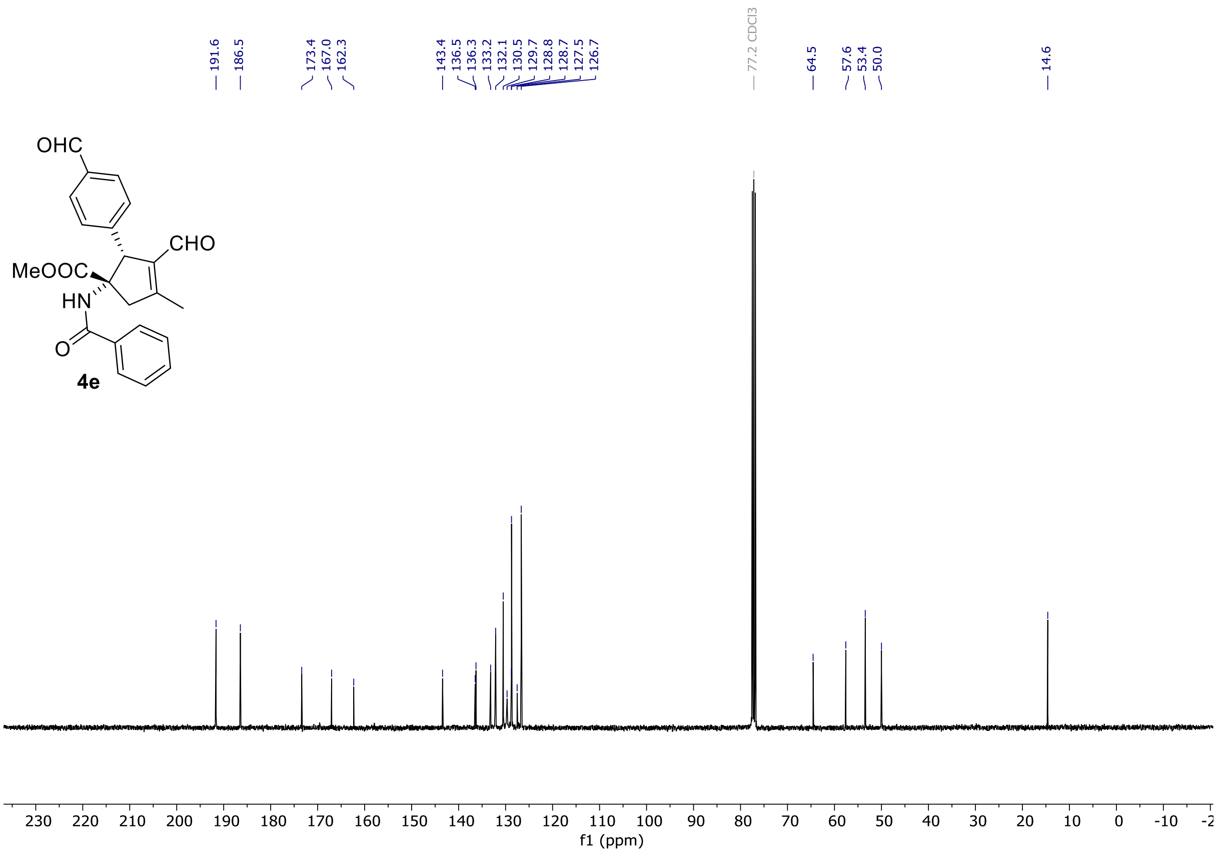

^{1}H NMR (400 MHz, $CDCl_3$)

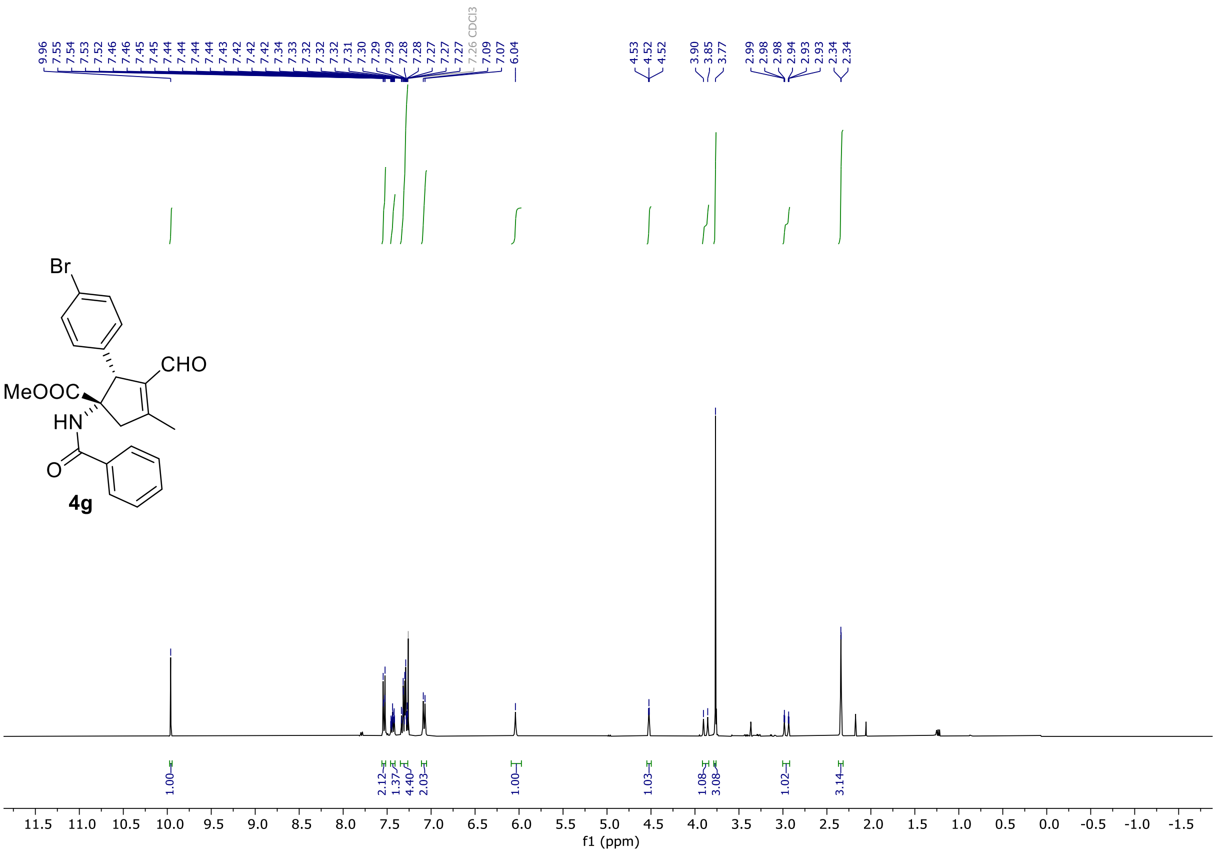

^{13}C{^{1}H} NMR (101 MHz, $CDCl_3$)

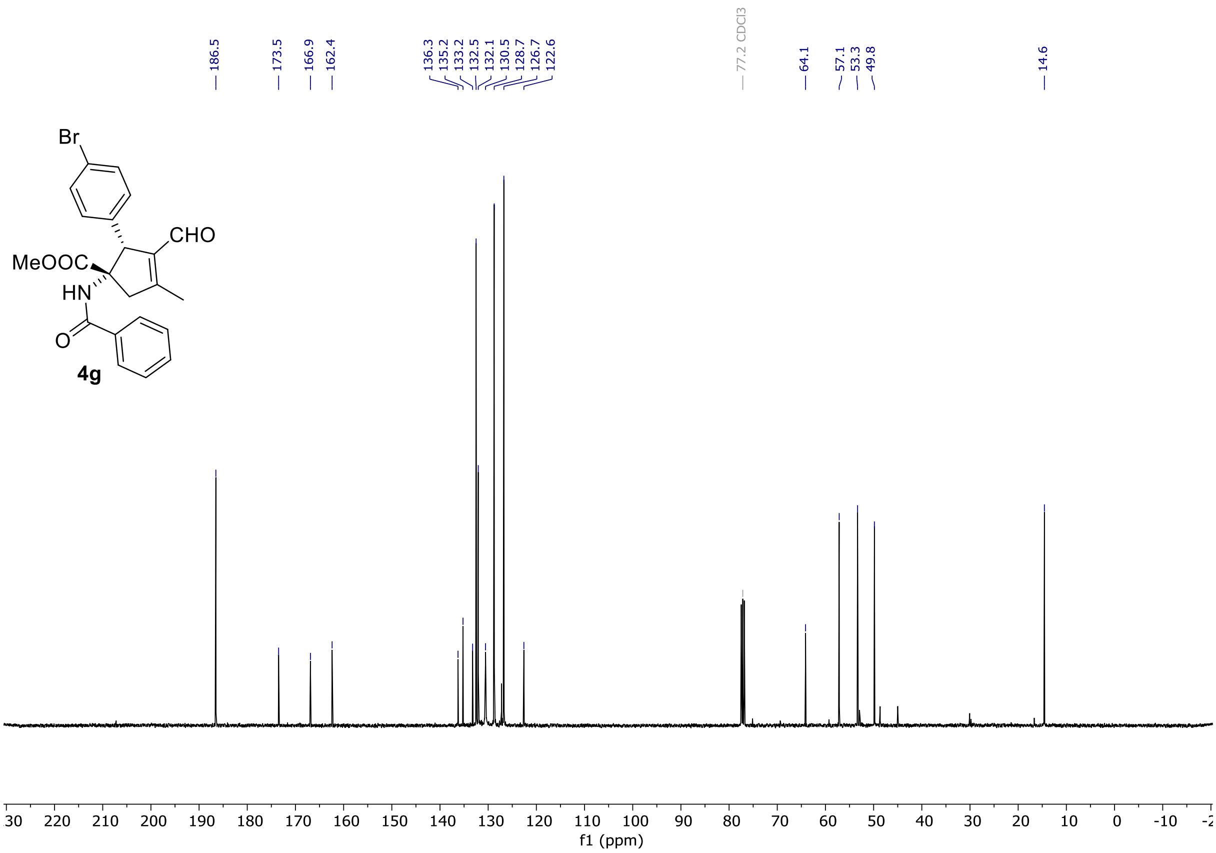

^{1}H NMR (400 MHz, $CDCl_3$)

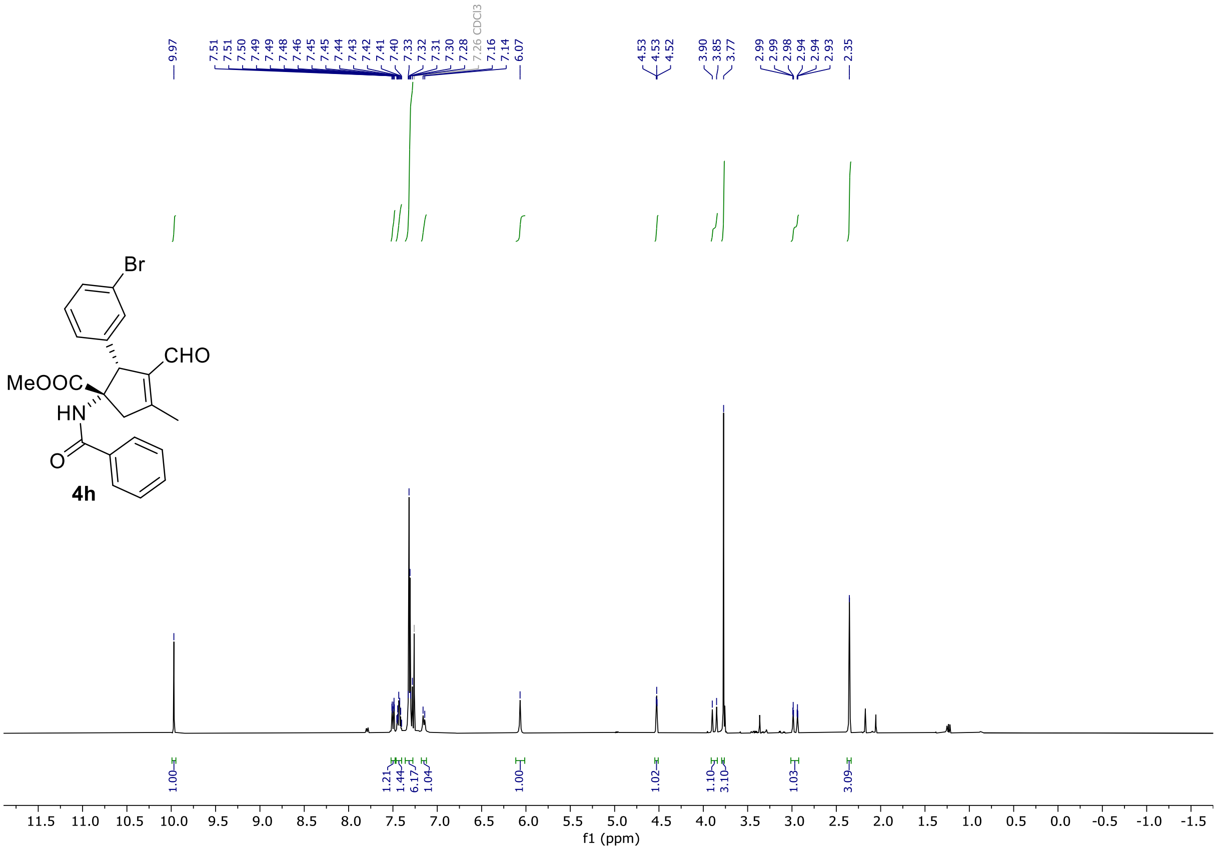

^{13}C{^{1}H} NMR (101 MHz, $CDCl_3$)

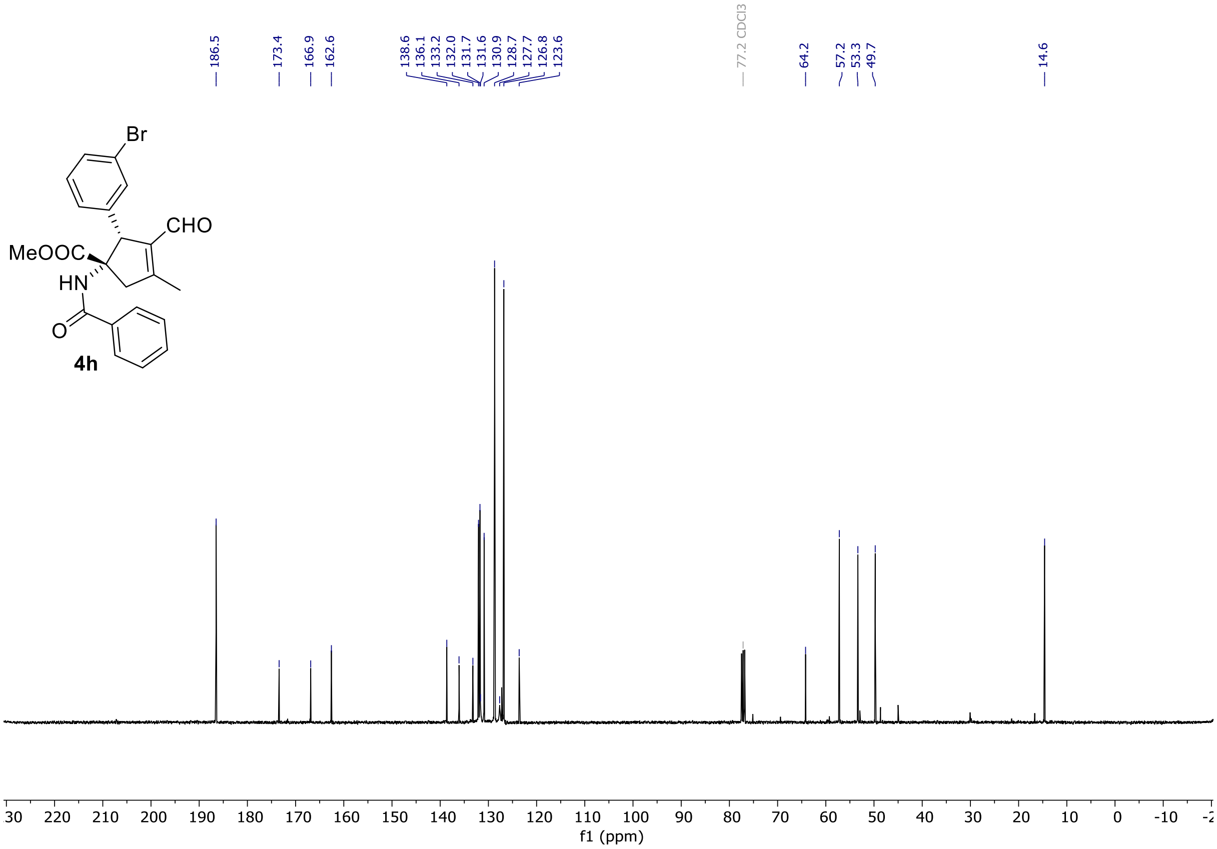

^{1}H NMR (400 MHz, $CDCl_3$)

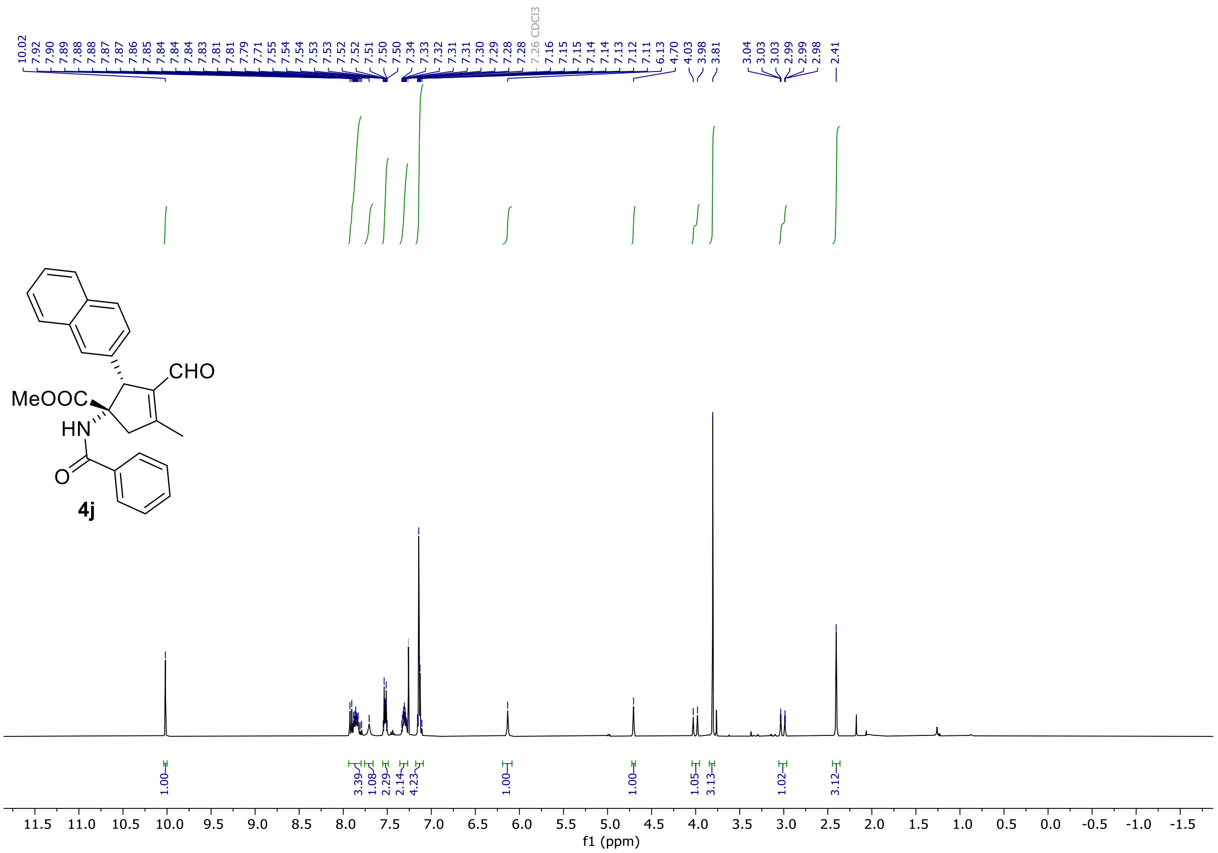

^{13}C{^{1}H} NMR (101 MHz, $CDCl_3$)

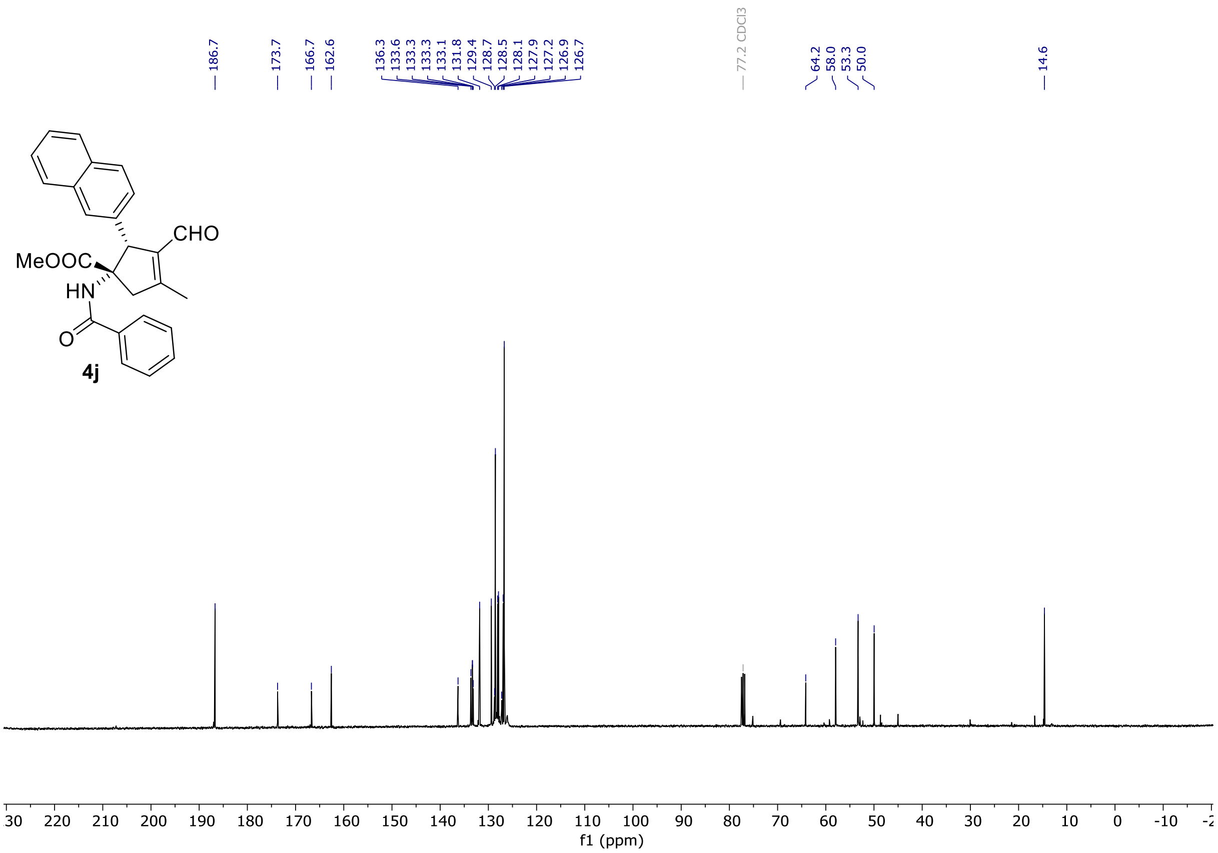

^{1}H NMR (400 MHz, $CDCl_3$)

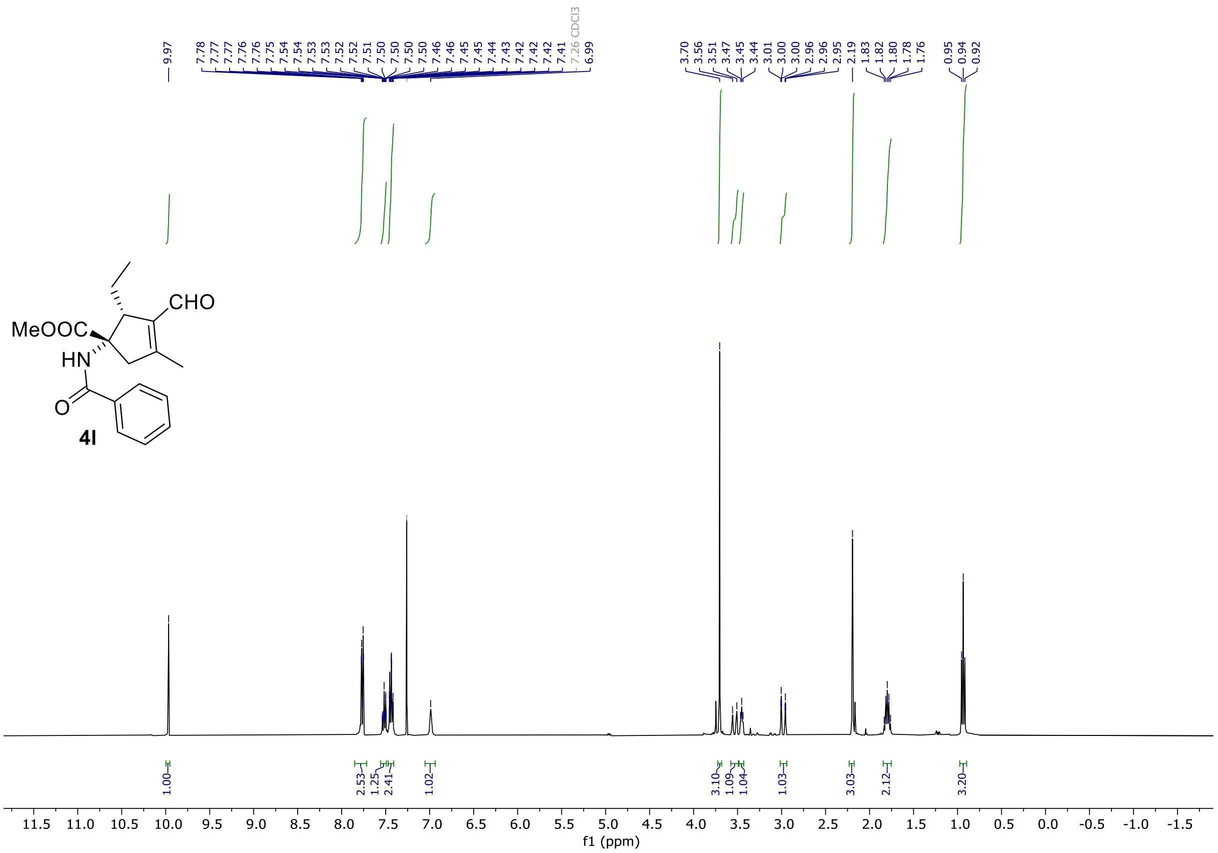

^{13}C{^{1}H} NMR (101 MHz, $CDCl_3$)

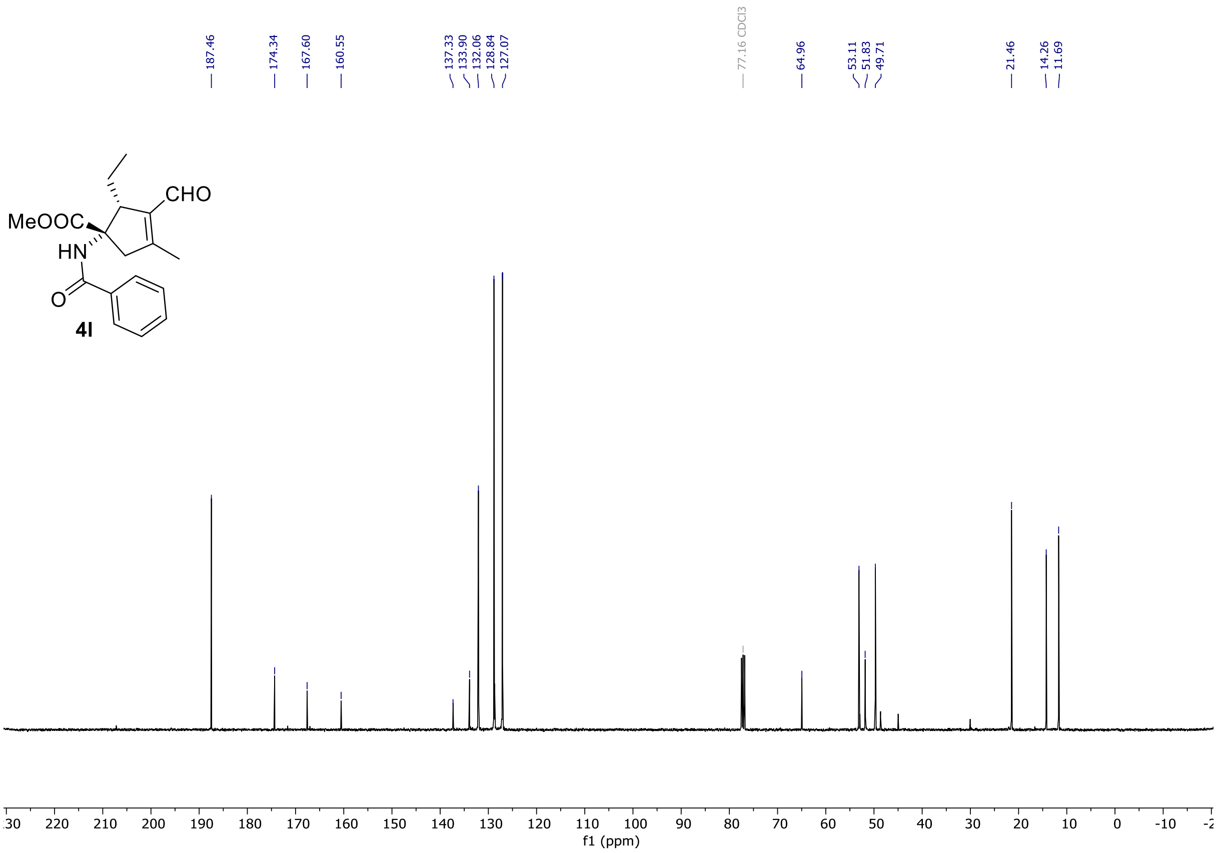

^{1}H NMR (400 MHz, $CDCl_3$)

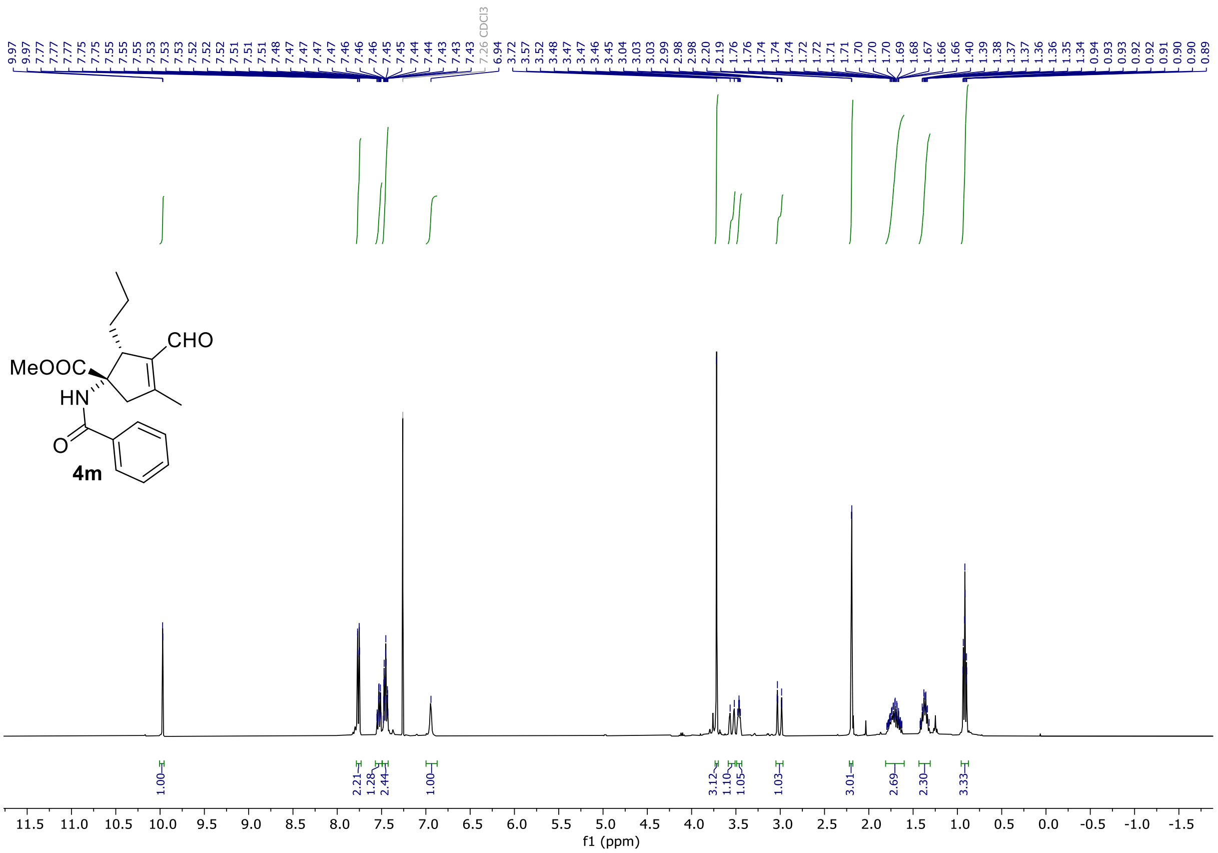

^{13}C{^{1}H} NMR (101 MHz, $CDCl_3$)

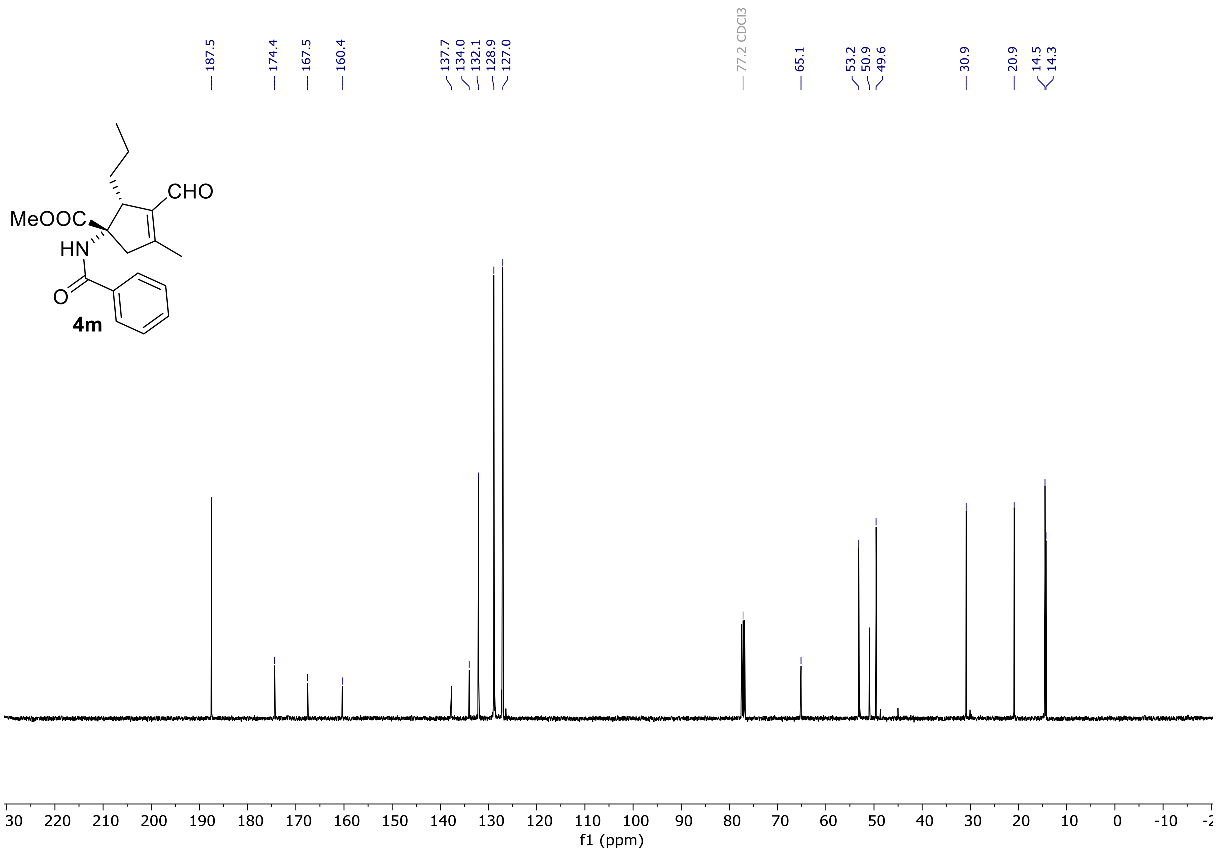

^{1}H NMR (400 MHz, $CDCl_3$)

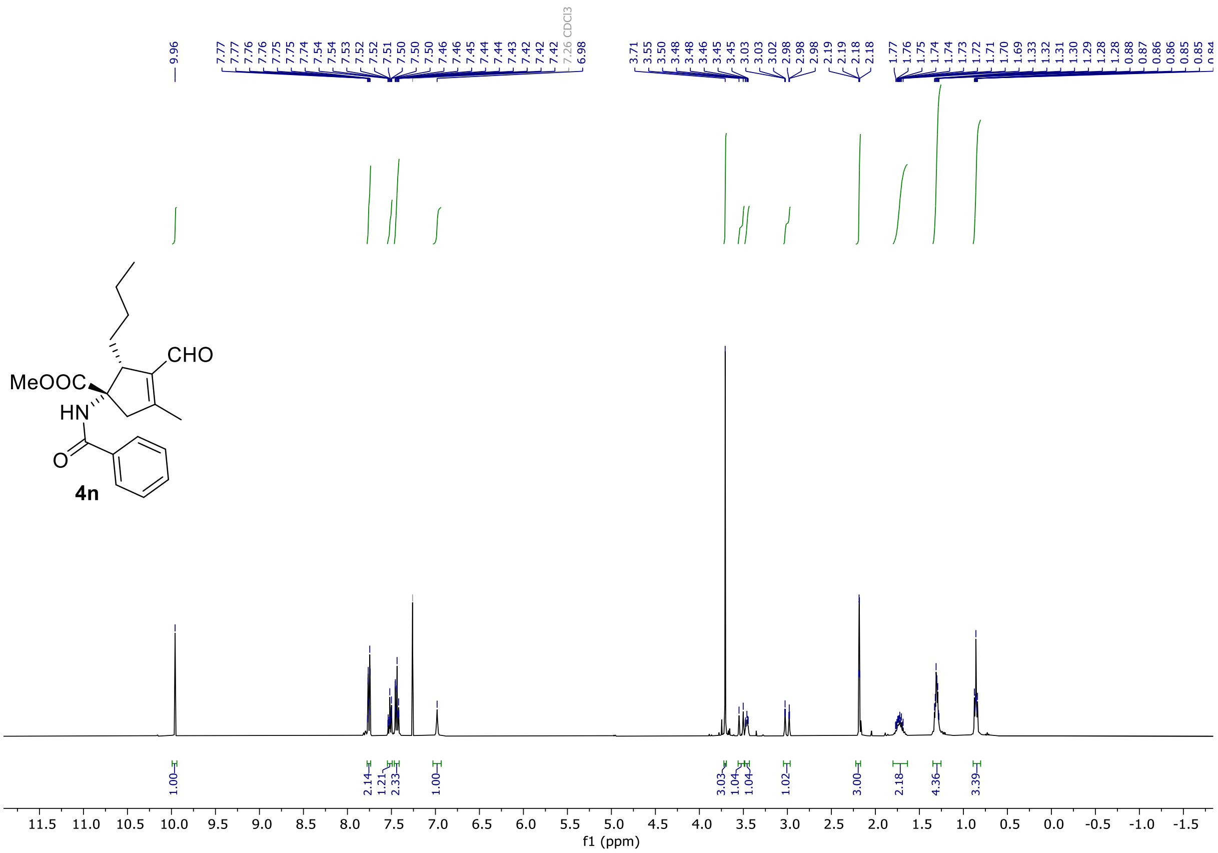

^{13}C{^{1}H} NMR (101 MHz, $CDCl_3$)

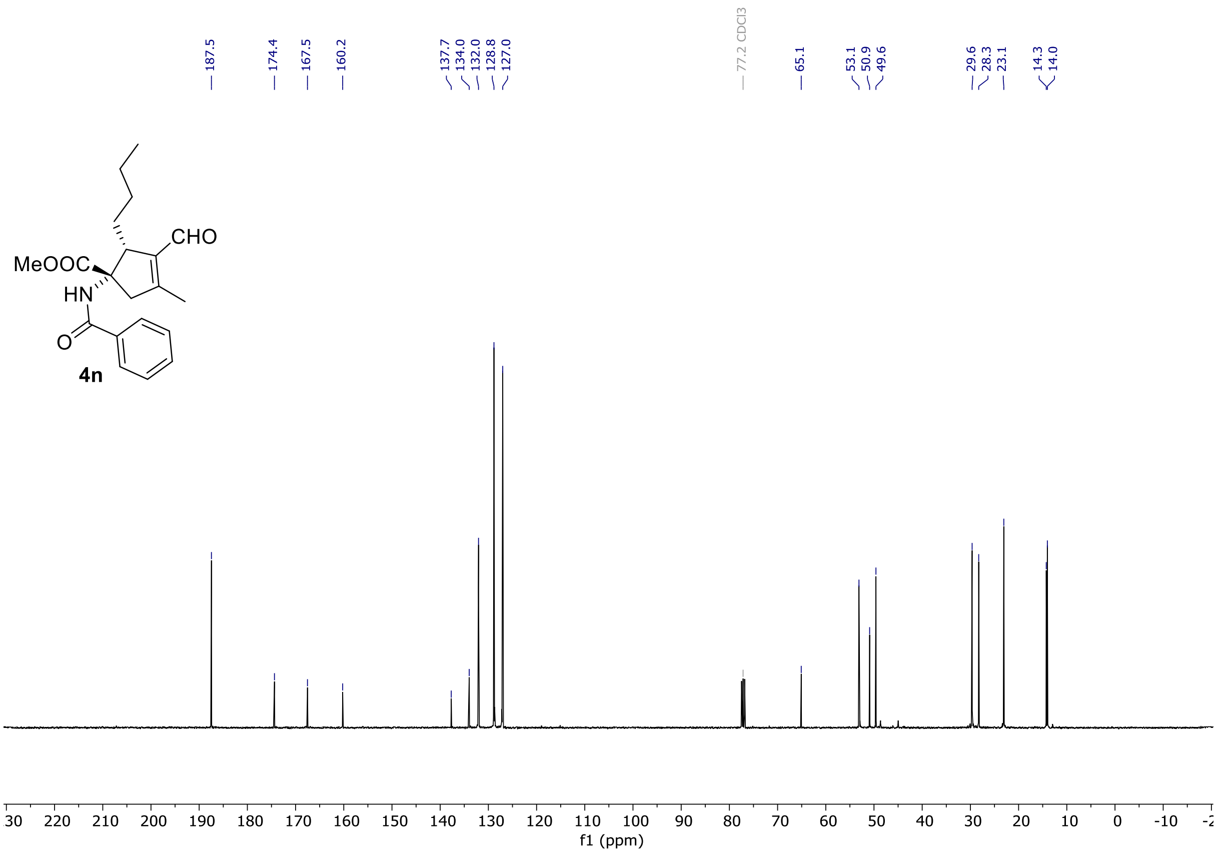

^{1}**H NMR** (400 MHz, $CDCl_3$)

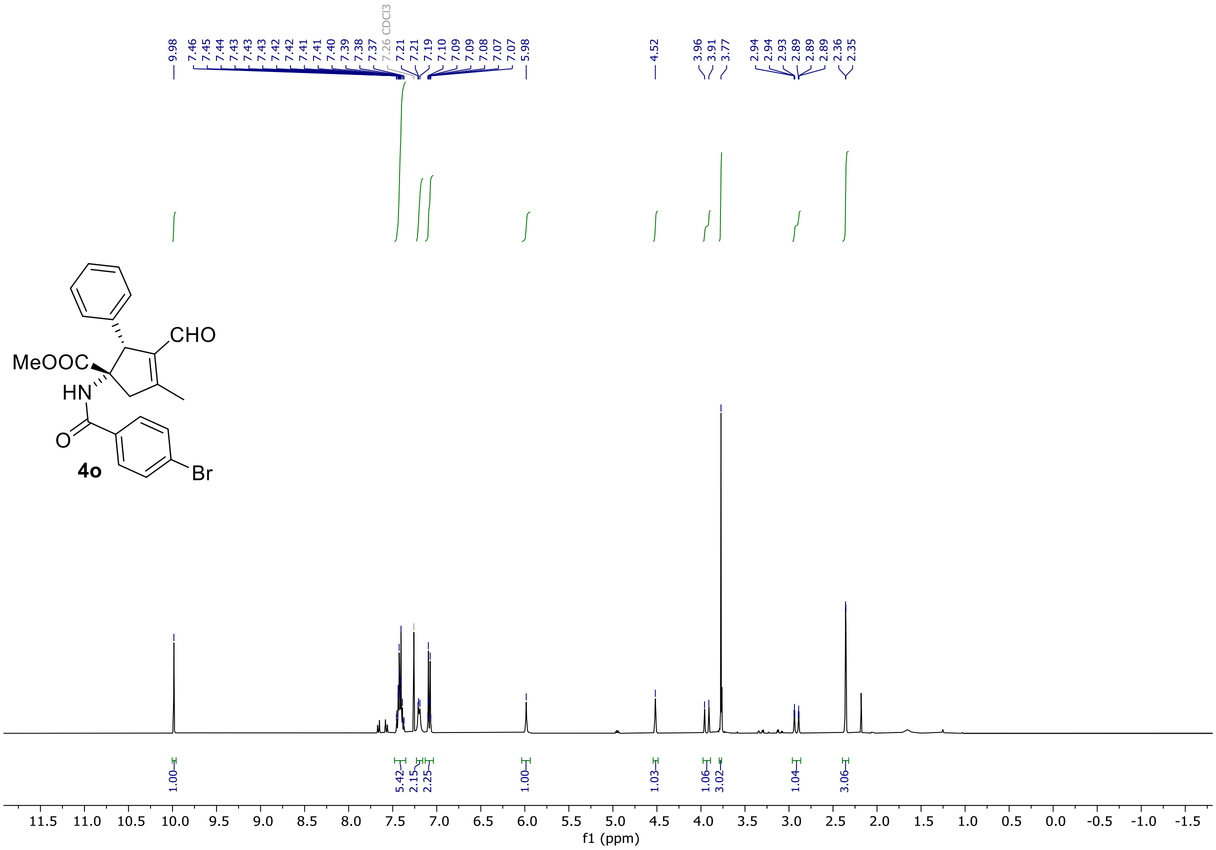

^{13}**C{^{1}H} NMR** (101 MHz, $CDCl_3$)

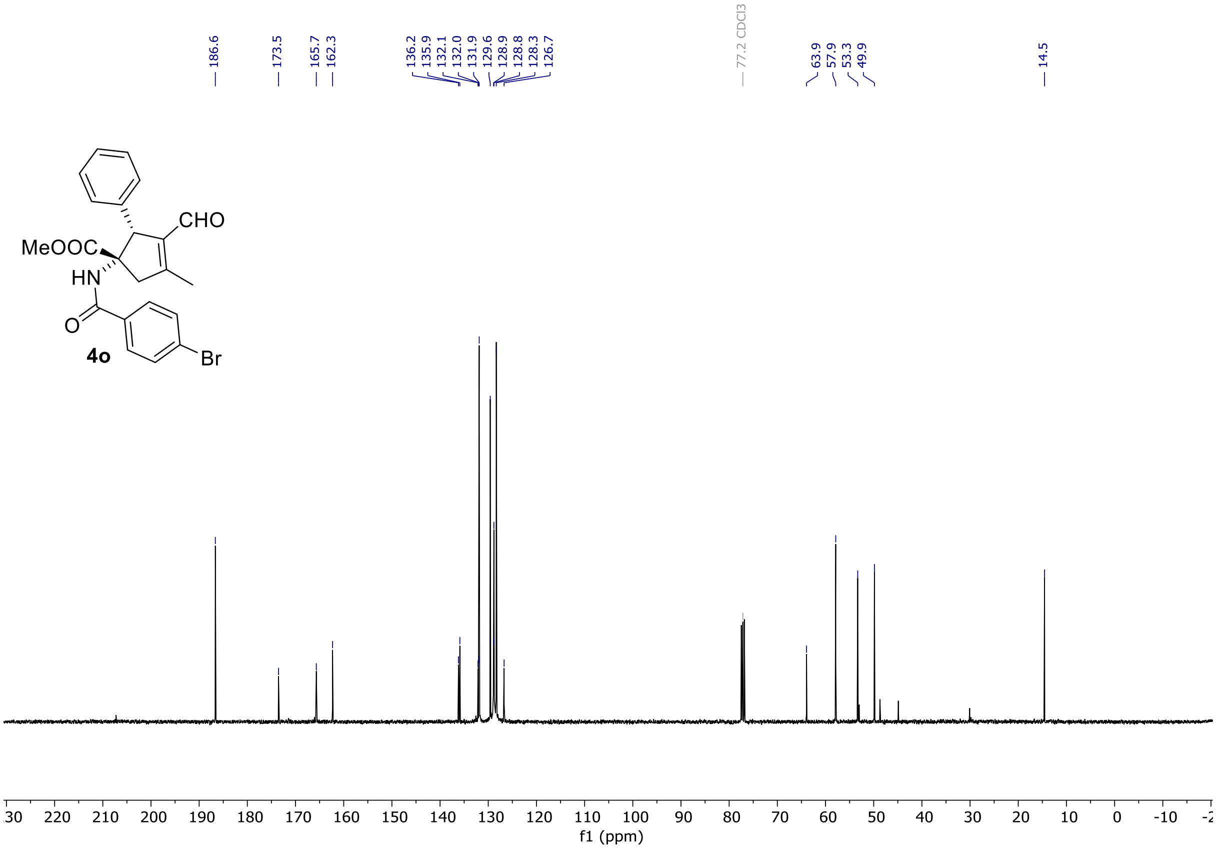

^{1}H NMR (400 MHz, $CDCl_3$)

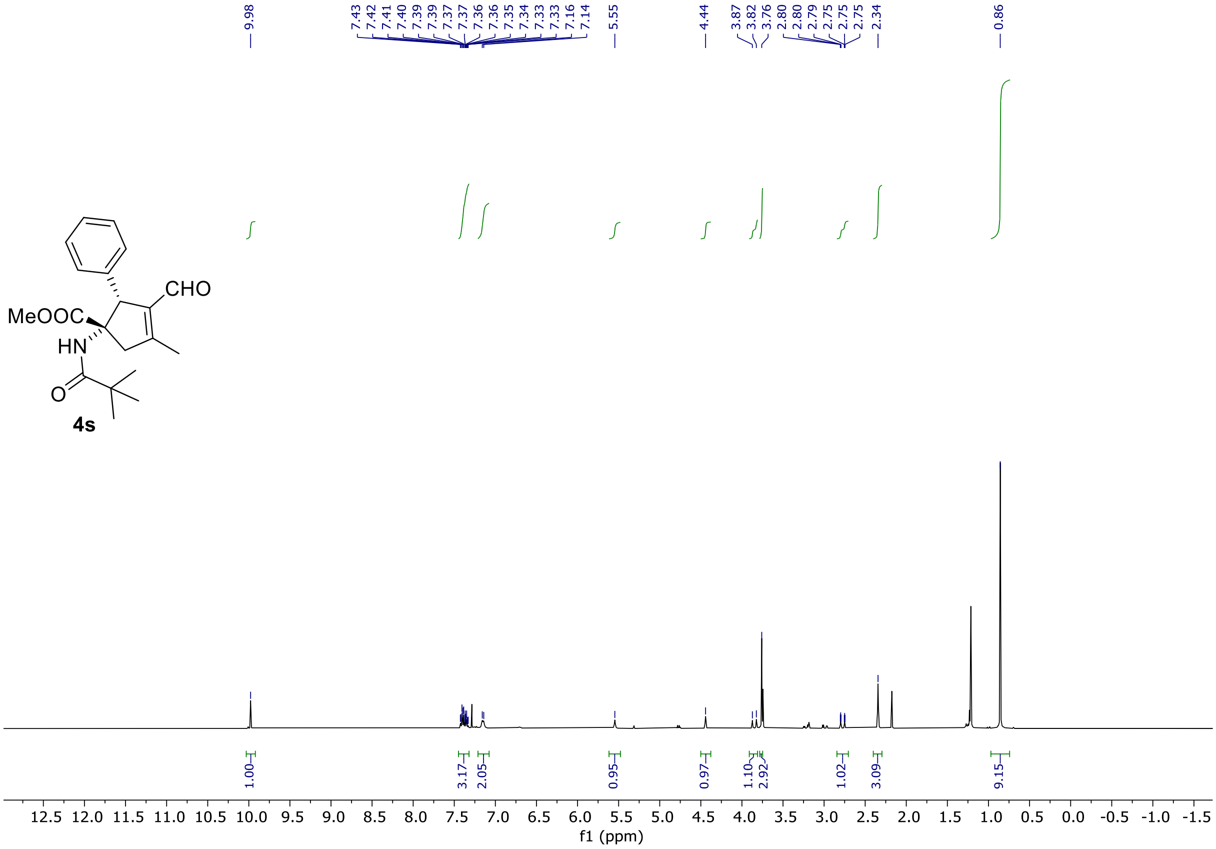

^{13}C{^{1}H} NMR (101 MHz, $CDCl_3$)

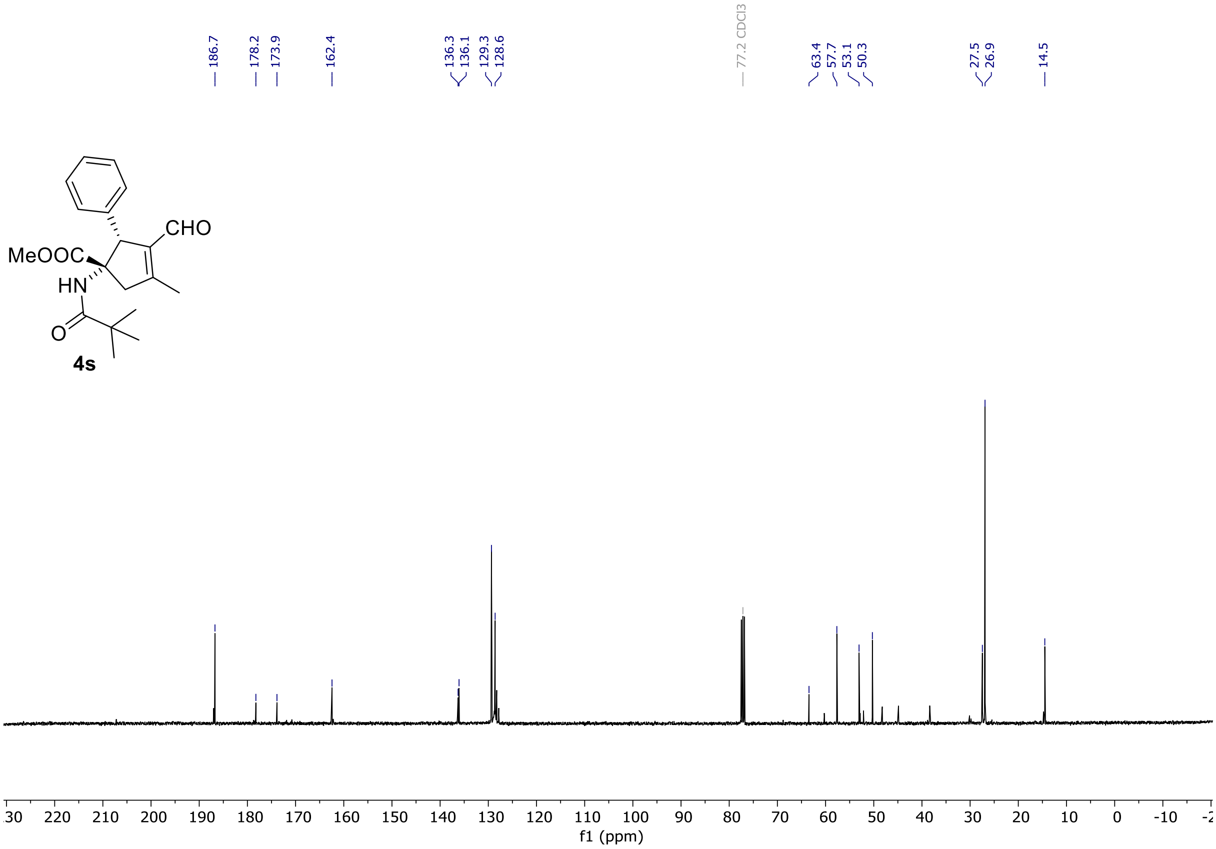

^{1}H NMR (400 MHz, CDCl$_3$)

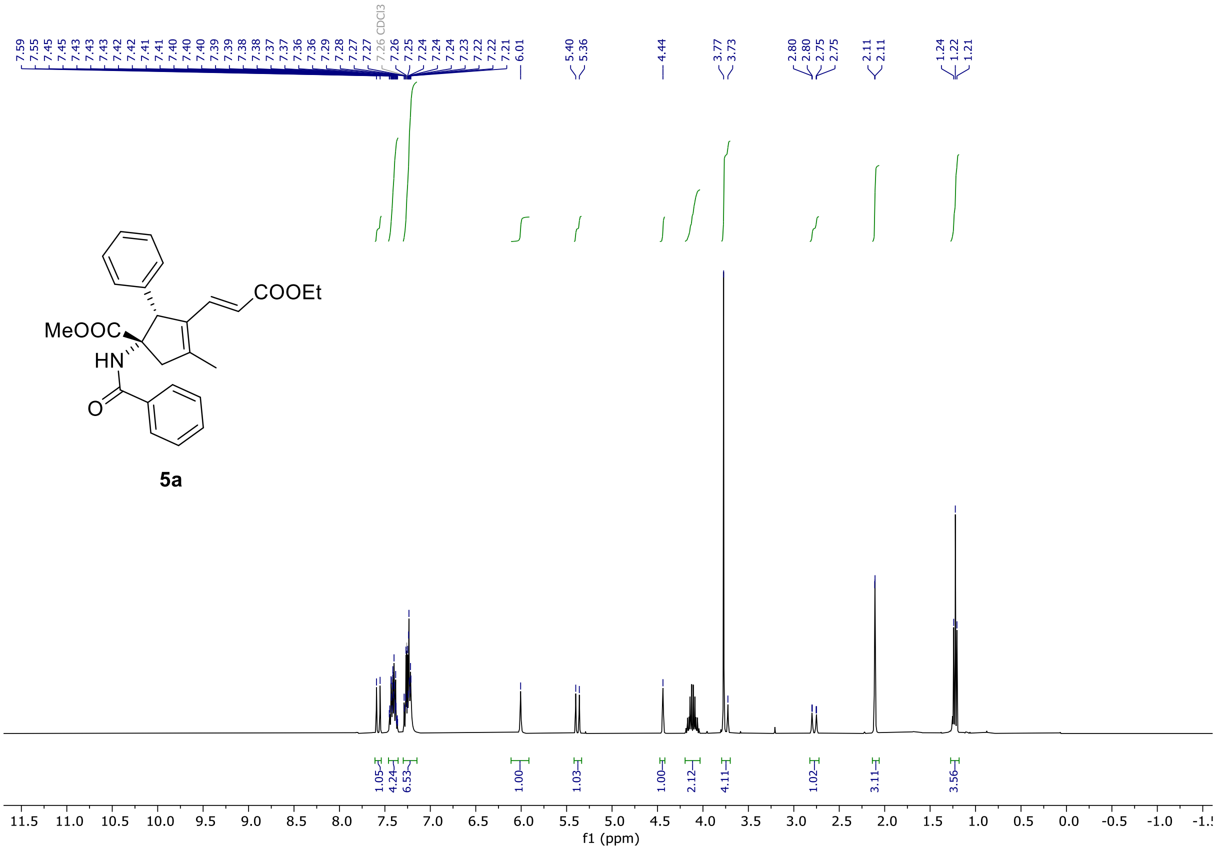

5a

^{13}C{^{1}H} NMR (101 MHz, CDCl$_3$)

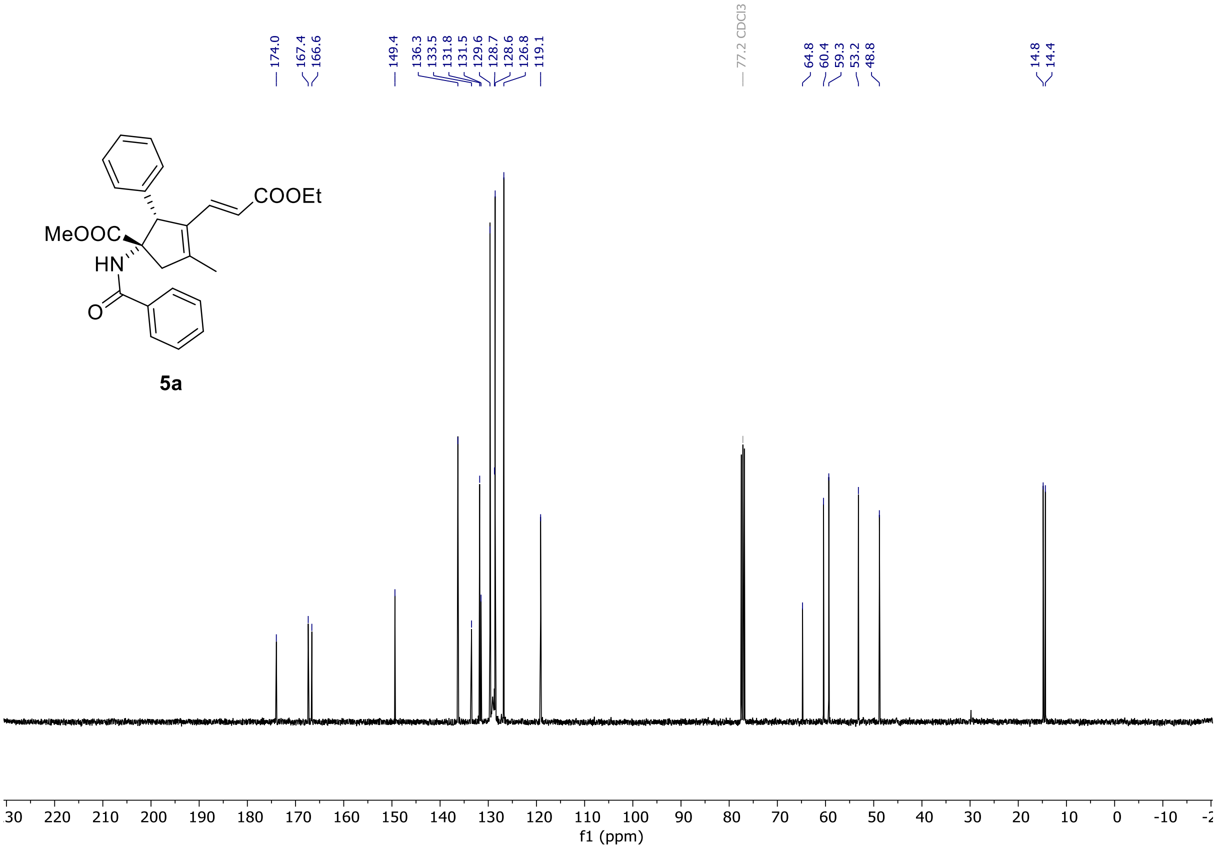

5a

^{1}H NMR (400 MHz, $CDCl_3$)

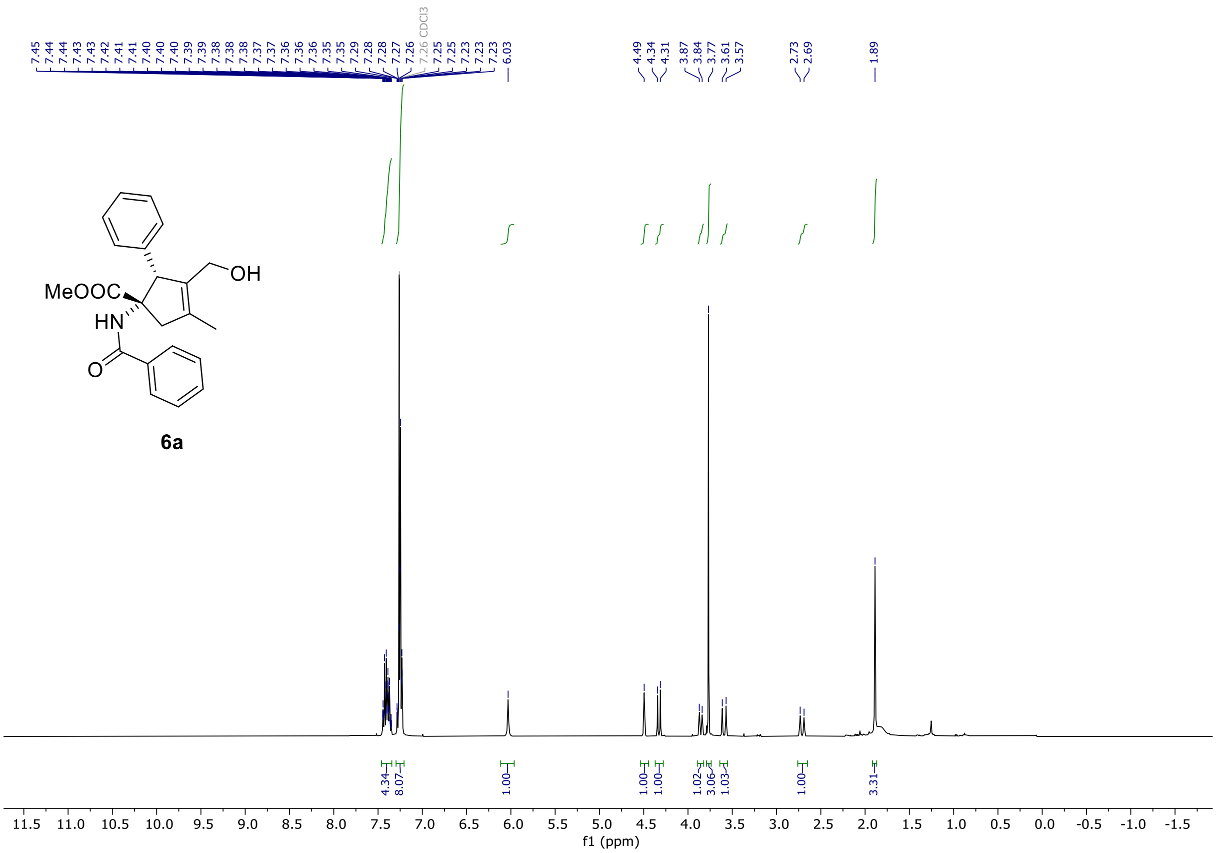

^{13}C{^{1}H} NMR (101 MHz, $CDCl_3$)

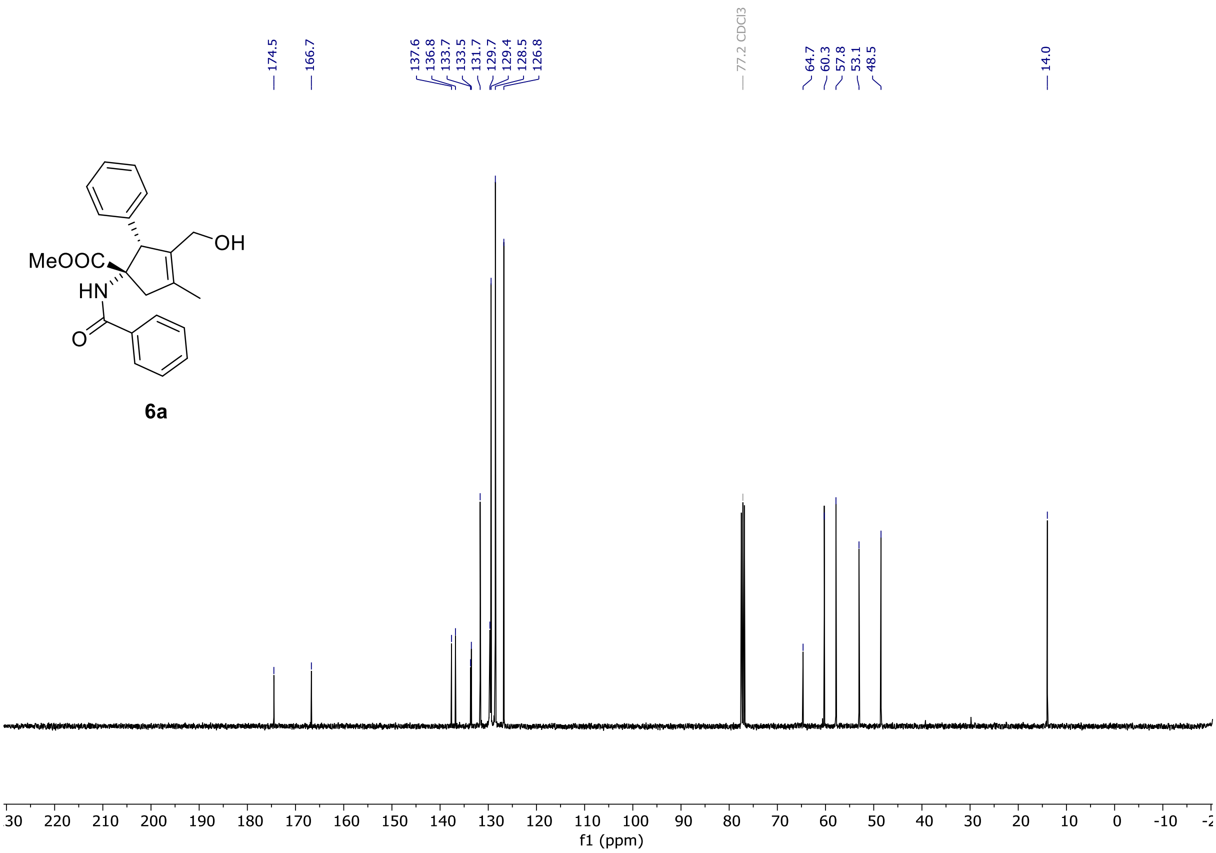

7 HPLC traces

4a

IA 80:20

Racemate

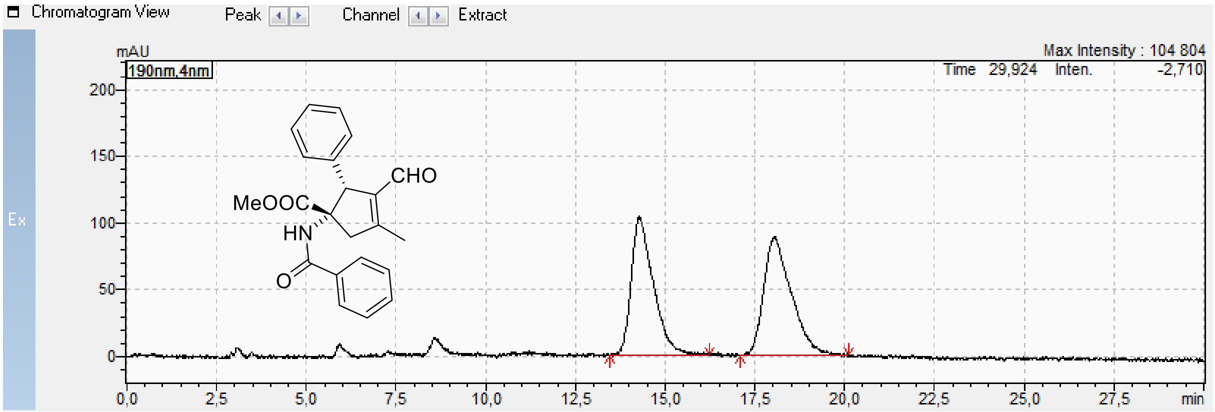

Results View - Peak Table

Peak Table | Compound | Group | Calibration Curve

Peak#	Ret. Time	Conc.	Area	Height	Similarity Index	Mark	Peak Start	Peak End	Area%
1	14,257	48,099	4444949	103685	0,000000	M	13,451	16,224	48,099
2	18,054	51,901	4796297	88859	0,000000	M	17,088	20,117	51,901

Chiral

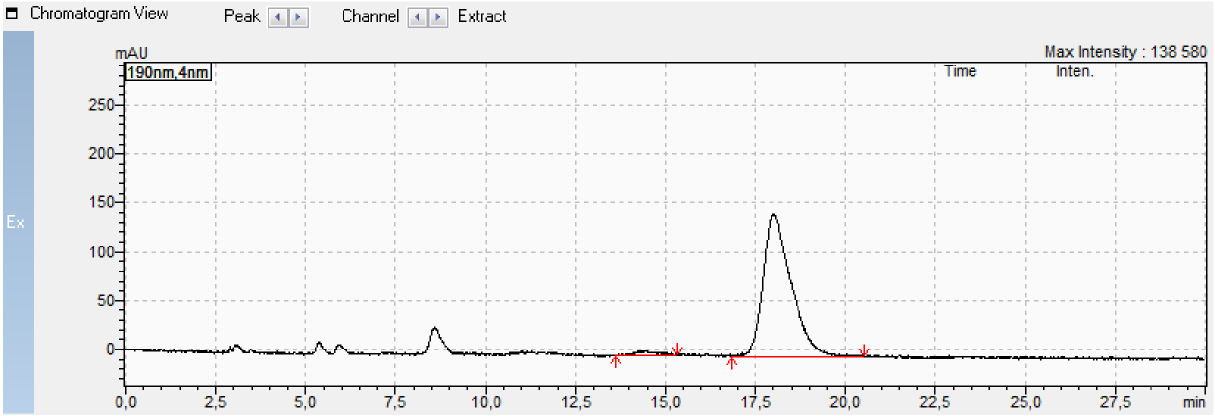

Results View - Peak Table

Peak Table | Compound | Group | Calibration Curve

Peak#	Ret. Time	Conc.	Area	Height	Similarity Index	Mark	Peak Start	Peak End	Area%
1	14,306	3,006	242851	5140	0,000000	M	13,632	15,317	3,006
2	17,995	96,994	7836797	145776	0,000000	M	16,832	20,501	96,994

4b

IA 70:30

Racemate

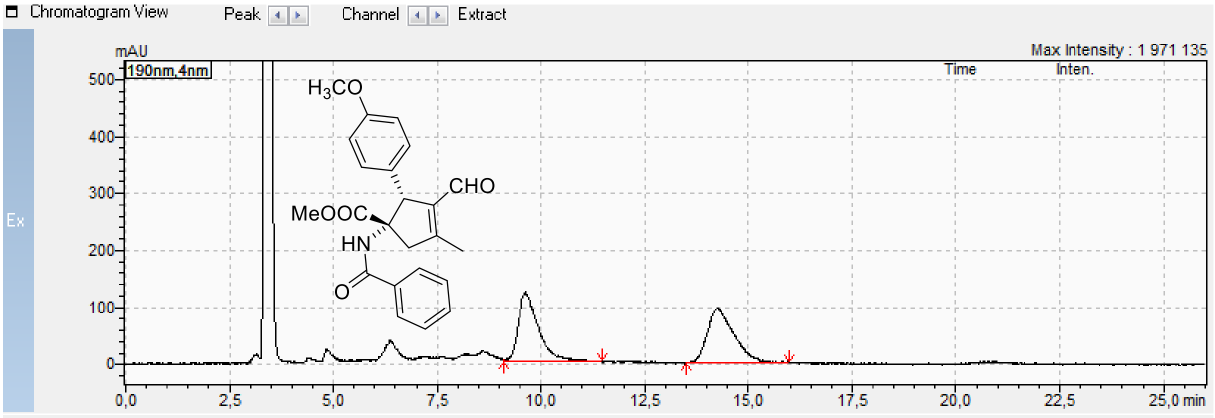

Results View - Peak Table

Peak Table | Compound | Group | Calibration Curve

Peak#	Ret. Time	Conc.	Area	Height	Similarity Index	Mark	Peak Start	Peak End	Area%
1	9,620	49,571	4088085	120336	0,000000	M	9,120	11,488	49,571
2	14,238	50,429	4158883	95008	0,000000	M	13,493	15,968	50,429

Chiral

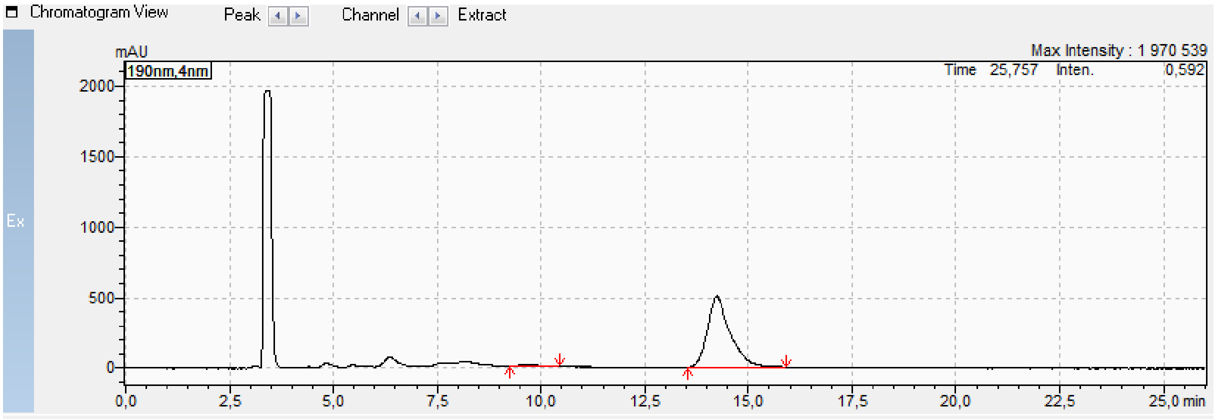

Results View - Peak Table

Peak Table | Compound | Group | Calibration Curve

Peak#	Ret. Time	Conc.	Area	Height	Similarity Index	Mark	Peak Start	Peak End	Area%
1	9,698	2,369	461968	15270	0,000000	M	9,248	10,443	2,369
2	14,249	97,631	19034861	508571	0,000000	M	13,525	15,904	97,631

4c

IA 80:20

Racemate

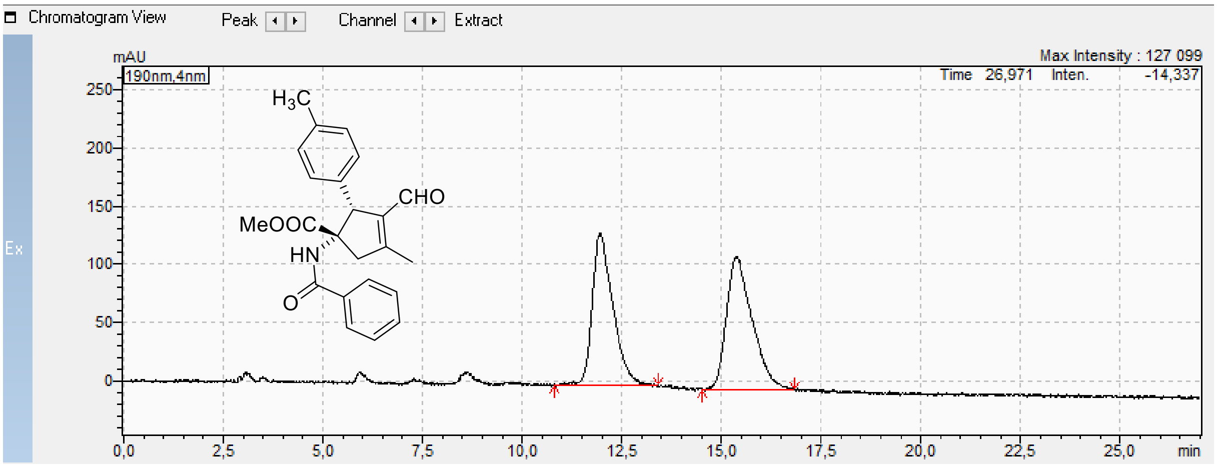

Results View - Peak Table

Peak Table | Compound | Group | Calibration Curve

Peak#	Ret. Time	Area	Height	Peak Start	Peak End	Mark	Conc.	Unit	Area%
1	11,949	4772106	130890	10,816	13,397	M	48,467		48,467
2	15,374	5073908	113461	14,528	16,832	M	51,533		51,533

Chiral

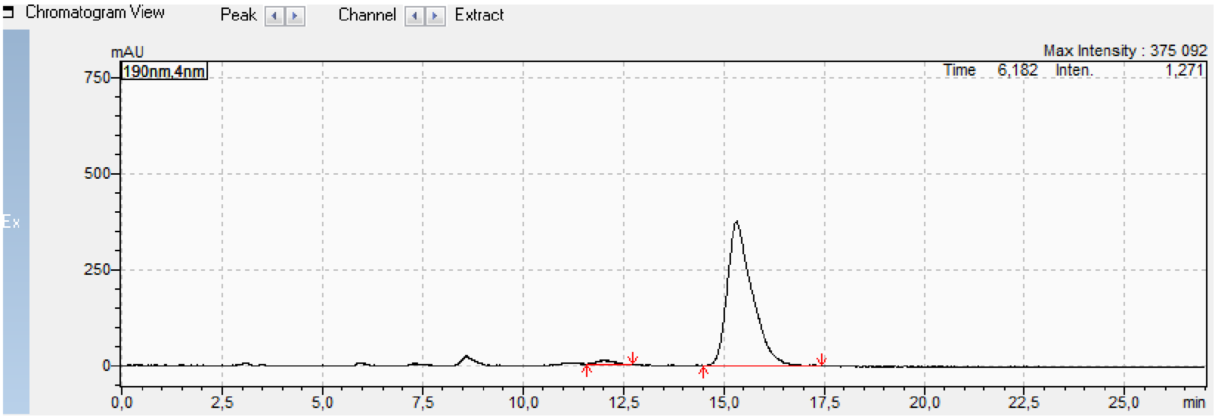

Results View - Peak Table

Peak Table | Compound | Group | Calibration Curve

Peak#	Ret. Time	Conc.	Area	Height	Similarity Index	Mark	Peak Start	Peak End	Area%
1	11,981	2,417	400783	12255	0,000000	M	11,584	12,736	2,417
2	15,331	97,583	16179257	375698	0,000000	M	14,496	17,429	97,583

4d

IA 70:30

Racemate

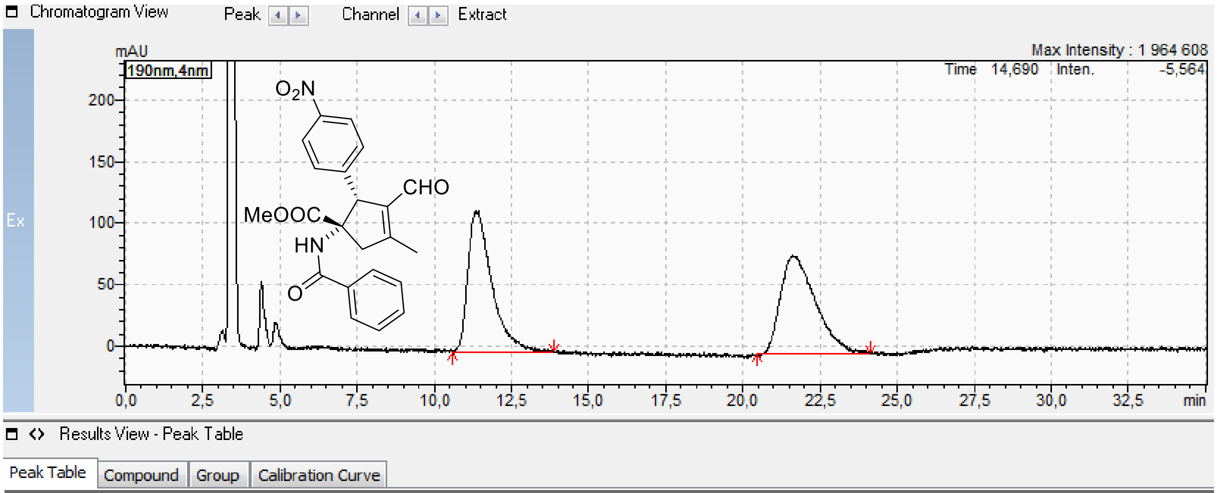

Peak#	Ret. Time	Conc.	Area	Height	Similarity Index	Mark	Peak Start	Peak End	Area%
1	11,427	49,261	6160999	113960	0,000000	M	10,592	13,877	49,261
2	21,614	50,739	6345973	80267	0,000000	M	20,469	24,149	50,739

Chiral

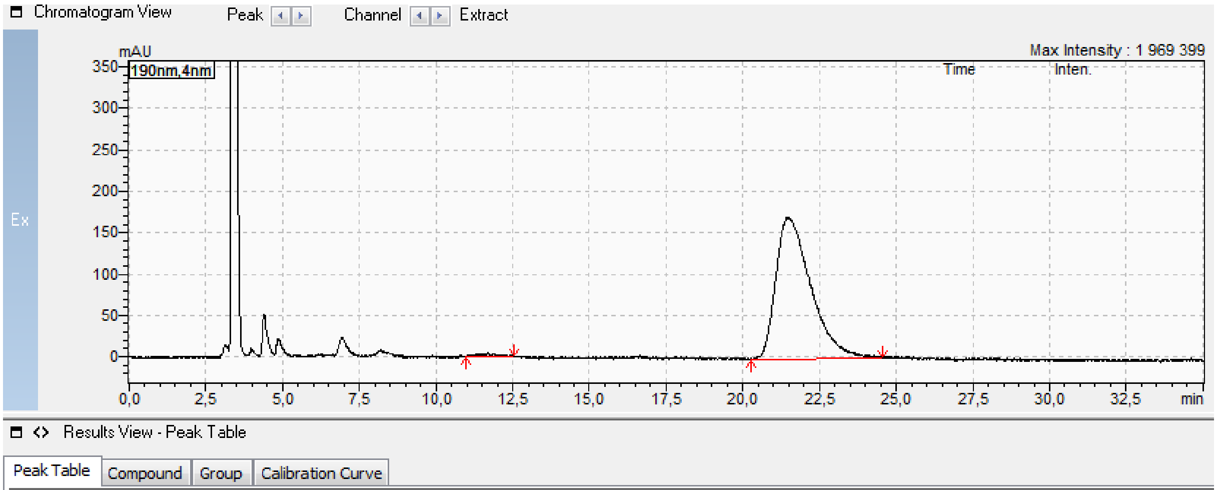

Peak#	Ret. Time	Conc.	Area	Height	Similarity Index	Mark	Peak Start	Peak End	Area%
1	11,682	1,534	210417	4935	0,000000	M	10,965	12,544	1,534
2	21,438	98,466	13505652	171215	0,000000	M	20,267	24,555	98,466

4g

IA 70:30

Racemate

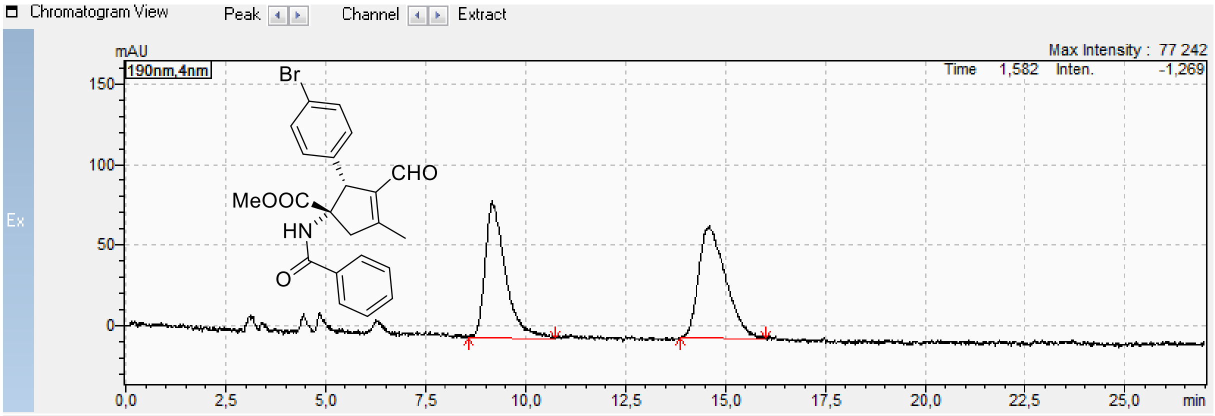

Results View - Peak Table

Peak Table | Compound | Group | Calibration Curve

Peak#	Ret. Time	Conc.	Area	Height	Similarity Index	Mark	Peak Start	Peak End	Area%
1	9,152	48,330	3042070	85142	0,000000	M	8,587	10,752	48,330
2	14,595	51,670	3252344	69533	0,000000	M	13,888	16,011	51,670

Chiral

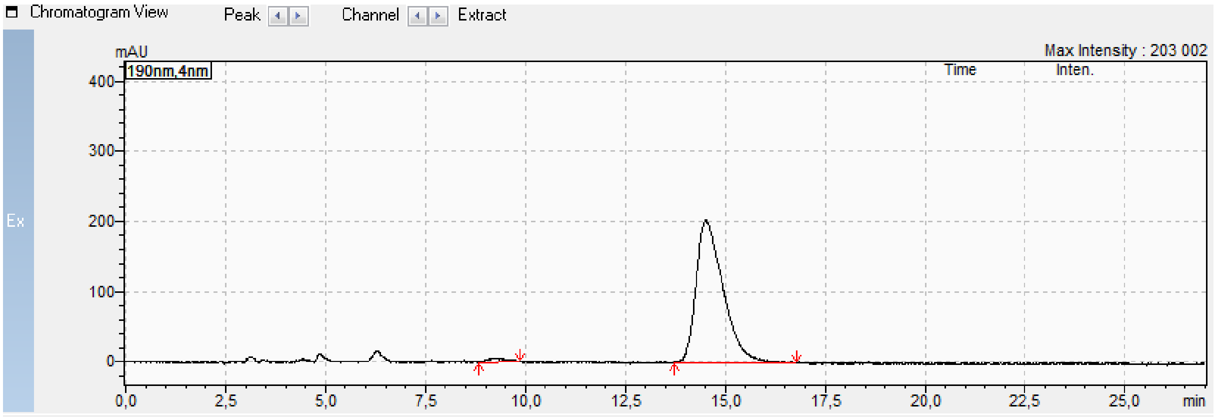

Results View - Peak Table

Peak Table | Compound | Group | Calibration Curve

Peak#	Ret. Time	Conc.	Area	Height	Similarity Index	Mark	Peak Start	Peak End	Area%
1	9,210	1,920	186968	6108	0,000000	M	8,843	9,867	1,920
2	14,508	98,080	9548604	204318	0,000000	M	13,717	16,789	98,080

4h

IA 70:30

Racemate

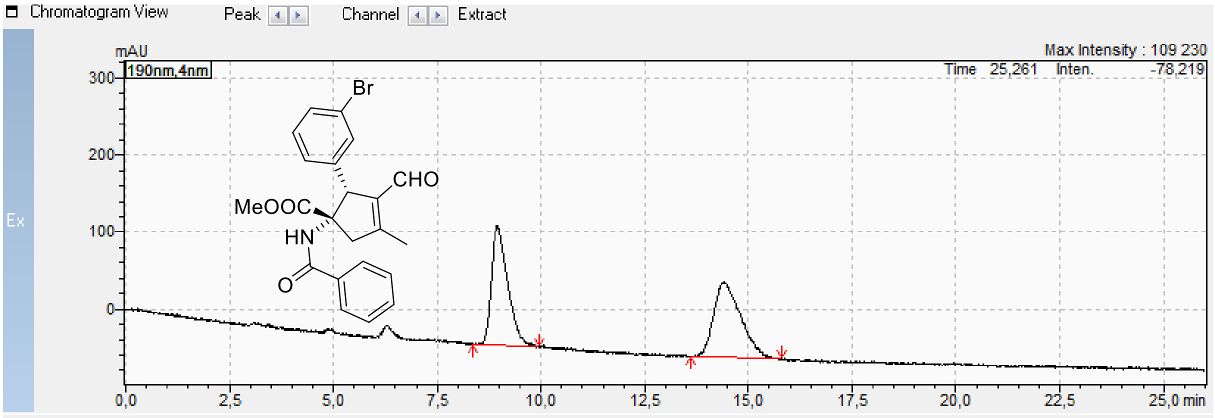

Results View - Peak Table

Peak Table | Compound | Group | Calibration Curve

Peak#	Ret. Time	Conc.	Area	Height	Similarity Index	Mark	Peak Start	Peak End	Area%
1	8,960	48,657	4197668	156372	0,000000	M	8,373	9,973	48,657
2	14,414	51,343	4429412	98278	0,000000	M	13,600	15,819	51,343

Chiral

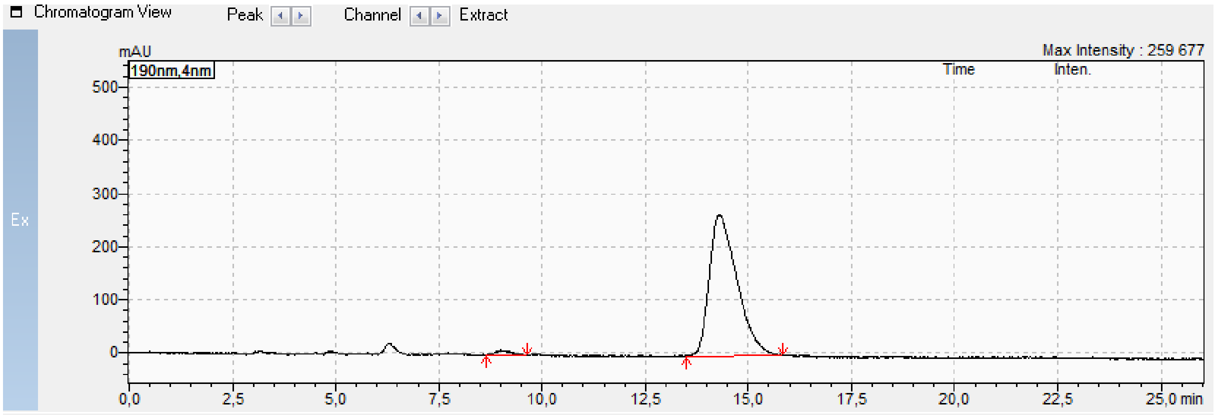

Results View - Peak Table

Peak Table | Compound | Group | Calibration Curve

Peak#	Ret. Time	Conc.	Area	Height	Similarity Index	Mark	Peak Start	Peak End	Area%
1	9,005	2,093	265422	10189	0,000000	M	8,640	9,621	2,093
2	14,302	97,907	12418498	265456	0,000000	M	13,493	15,851	97,907

4j

IA 80:20

Racemate

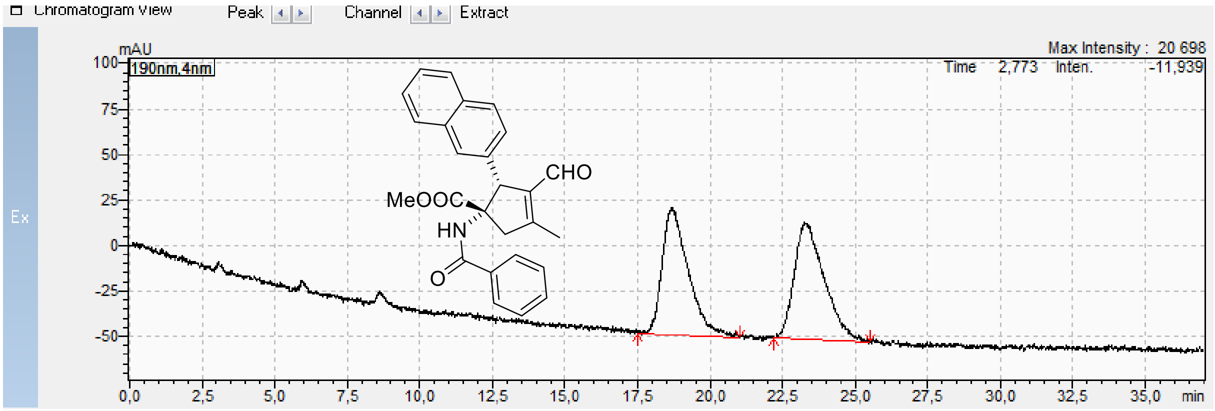

Results View - Peak Table

Peak Table | Compound | Group | Calibration Curve

Peak#	Ret. Time	Conc.	Area	Height	Similarity Index	Mark	Peak Start	Peak End	Area%
1	18,681	48,637	4352505	69621	0,000000	M	17,515	21,035	48,637
2	23,252	51,363	4596491	64465	0,000000	M	22,176	25,515	51,363

Chiral

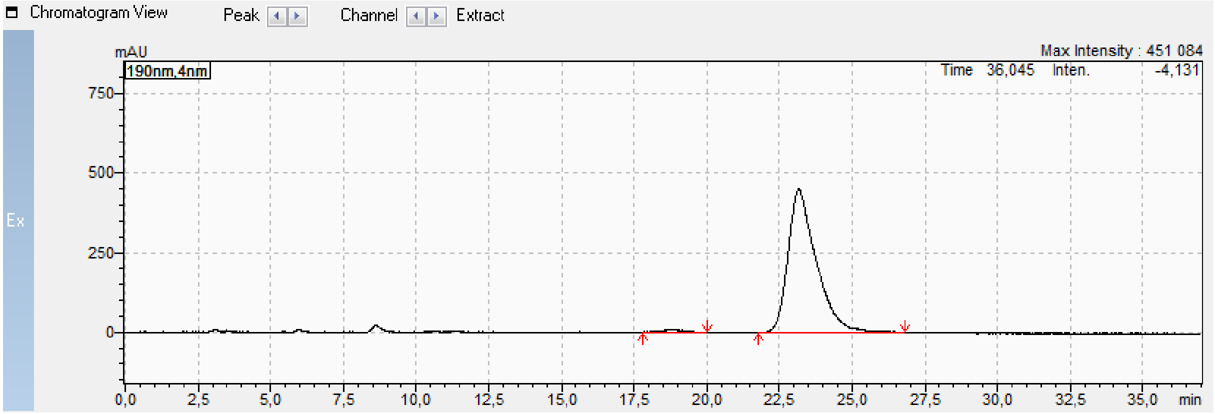

Results View - Peak Table

Peak Table | Compound | Group | Calibration Curve

Peak#	Ret. Time	Conc.	Area	Height	Similarity Index	Mark	Peak Start	Peak End	Area%
1	18,754	1,490	461085	9159	0,000000	M	17,813	19,989	1,490
2	23,174	98,510	30478142	452205	0,000000	M	21,781	26,795	98,510

4l

IA 80:20

Racemate

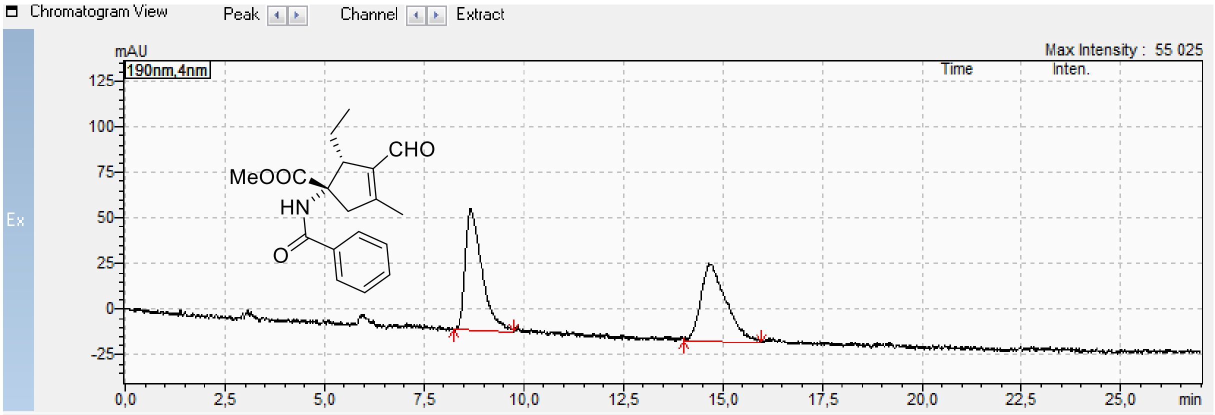

Results View - Peak Table

Peak Table | Compound | Group | Calibration Curve

Peak#	Ret. Time	Conc.	Area	Height	Similarity Index	Mark	Peak Start	Peak End	Area%
1	8,666	50,643	1921305	66431	0,000000	M	8,235	9,760	50,643
2	14,659	49,357	1872520	42550	0,000000	M	14,005	15,979	49,357

Chiral

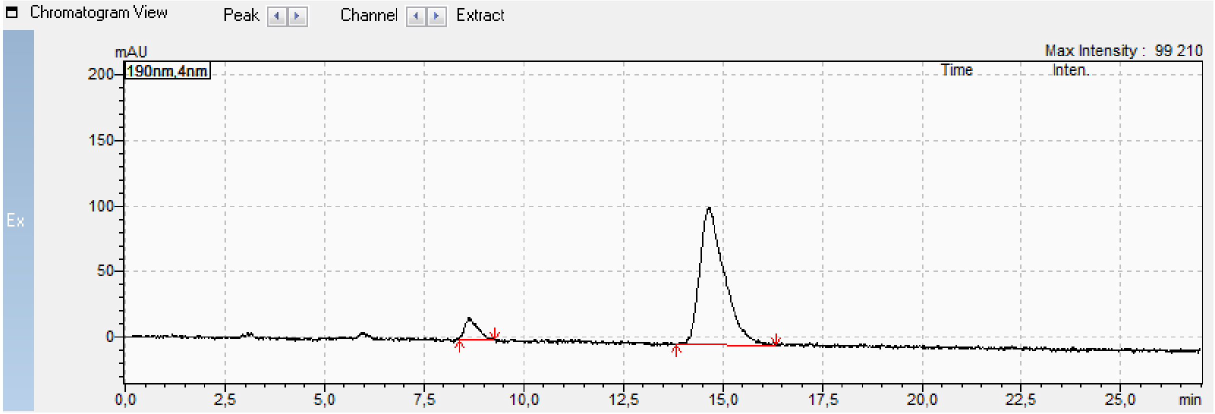

Results View - Peak Table

Peak Table | Compound | Group | Calibration Curve

Peak#	Ret. Time	Conc.	Area	Height	Similarity Index	Mark	Peak Start	Peak End	Area%
1	8,662	7,559	369990	16736	0,000000	M	8,373	9,280	7,559
2	14,655	92,441	4524711	105251	0,000000	M	13,835	16,320	92,441

4m

IA 80:20

Racemate

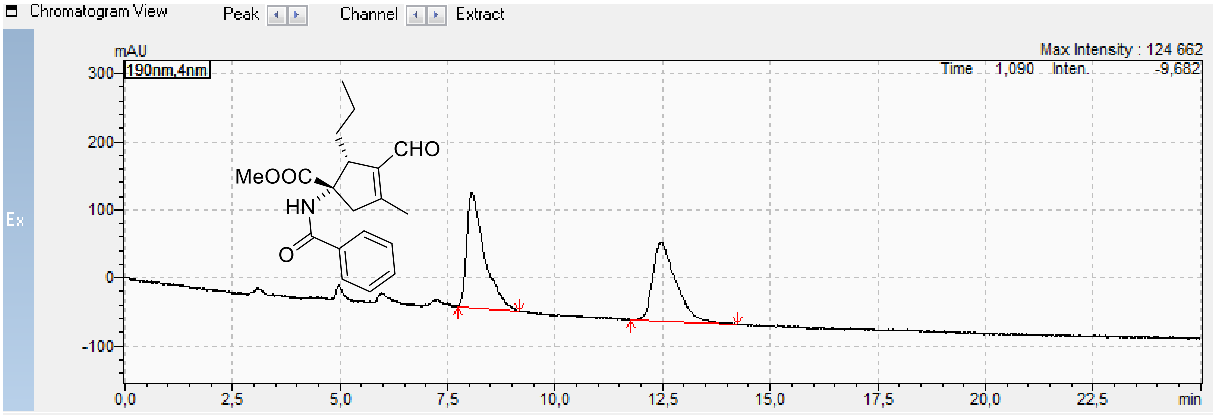

Results View - Peak Table

Peak Table | Compound | Group | Calibration Curve

Peak#	Ret. Time	Conc.	Area	Height	Similarity Index	Mark	Peak Start	Peak End	Area%
1	8,059	51,911	4782989	167961	0,000000	M	7,723	9,163	51,911
2	12,466	48,089	4430874	116847	0,000000	M	11,755	14,240	48,089

Chiral

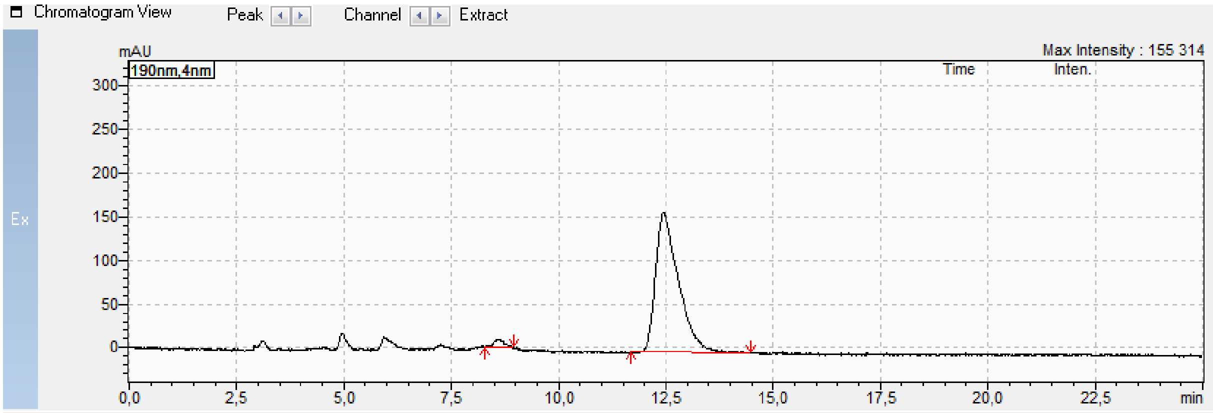

Results View - Peak Table

Peak Table | Compound | Group | Calibration Curve

Peak#	Ret. Time	Conc.	Area	Height	Similarity Index	Mark	Peak Start	Peak End	Area%
1	8,597	2,461	148225	8946	0,000000	M	8,277	8,971	2,461
2	12,454	97,539	5873800	160390	0,000000	M	11,680	14,464	97,539

4n

IA 80:20

Racemate

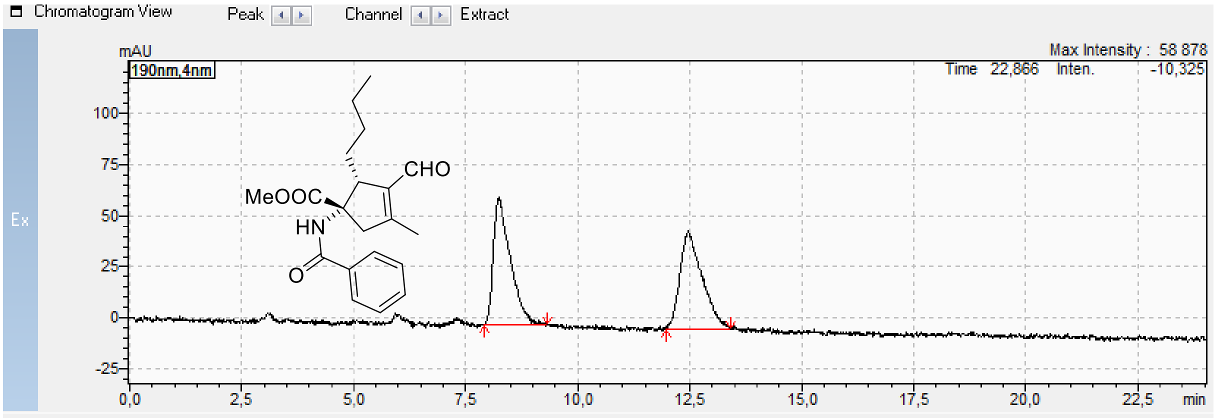

Results View - Peak Table

Peak Table | Compound | Group | Calibration Curve

Peak#	Ret. Time	Conc.	Area	Height	Similarity Index	Mark	Peak Start	Peak End	Area%
1	8,237	50,237	1650633	62395	0,000000	M	7,915	9,312	50,237
2	12,458	49,763	1635063	47682	0,000000	M	11,957	13,408	49,763

Chiral

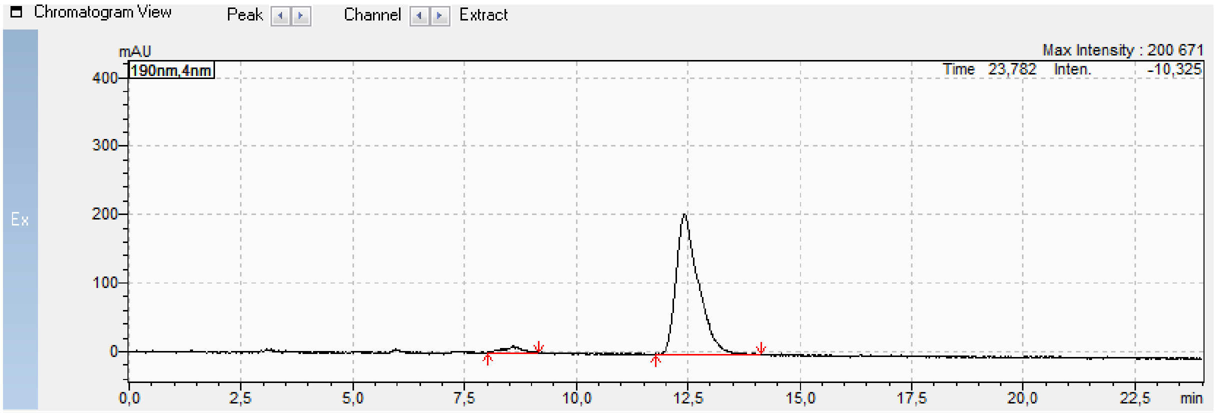

Results View - Peak Table

Peak Table | Compound | Group | Calibration Curve

Peak#	Ret. Time	Conc.	Area	Height	Similarity Index	Mark	Peak Start	Peak End	Area%
1	8,585	3,811	284377	9890	0,000000	M	8,011	9,152	3,811
2	12,418	96,189	7178261	205346	0,000000	M	11,765	14,133	96,189

4o

IB 70:30

Racemate

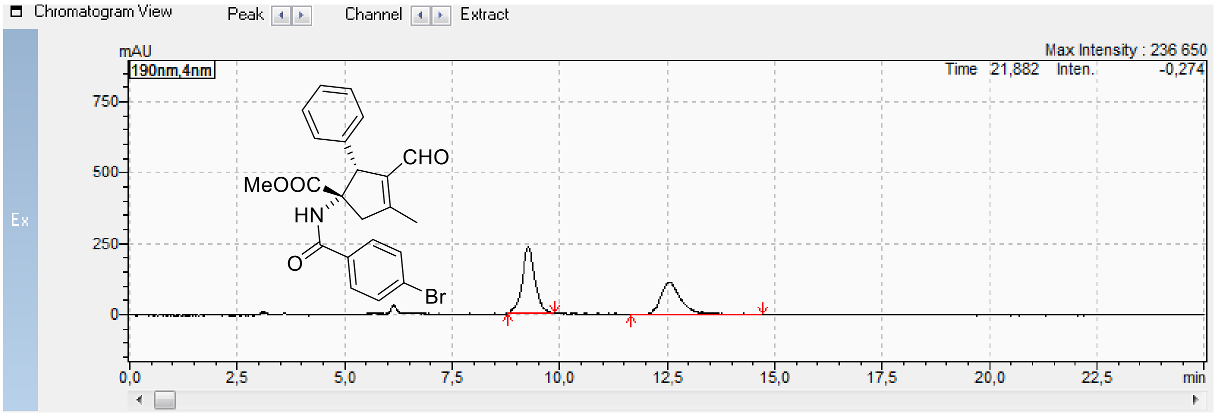

Results View - Peak Table

Peak Table | Compound | Group | Calibration Curve

Peak#	Ret. Time	Conc.	Area	Height	Similarity Index	Mark	Peak Start	Peak End	Area%
1	9,273	53,627	4791880	231707	0,000000	M	8,811	9,877	53,627
2	12,572	46,373	4143684	115628	0,000000	M	11,659	14,720	46,373

Chiral

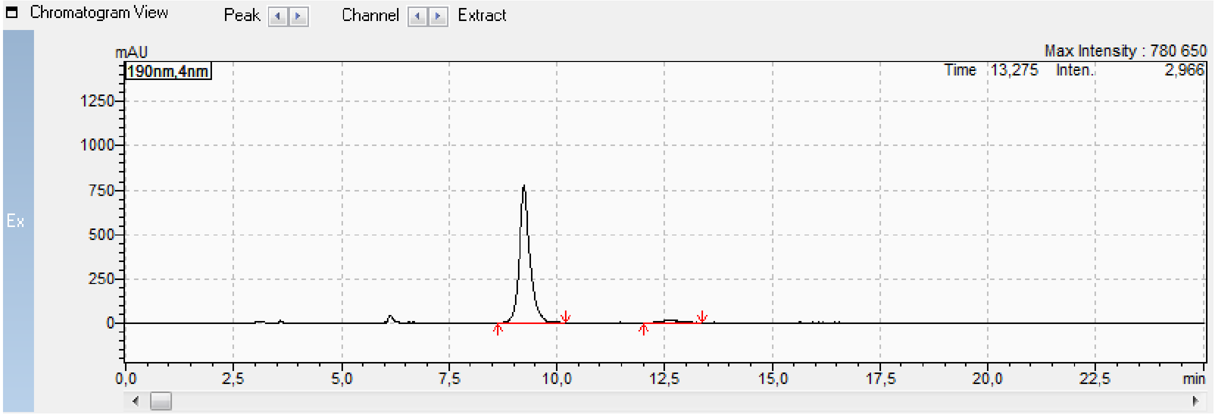

Results View - Peak Table

Peak Table | Compound | Group | Calibration Curve

Peak#	Ret. Time	Conc.	Area	Height	Similarity Index	Mark	Peak Start	Peak End	Area%
1	9,238	96,292	13336510	779142	0,000000	M	8,651	10,219	96,292
2	12,664	3,708	513537	15145	0,000000	M	12,000	13,387	3,708

4s

IA 80:20

Racemate

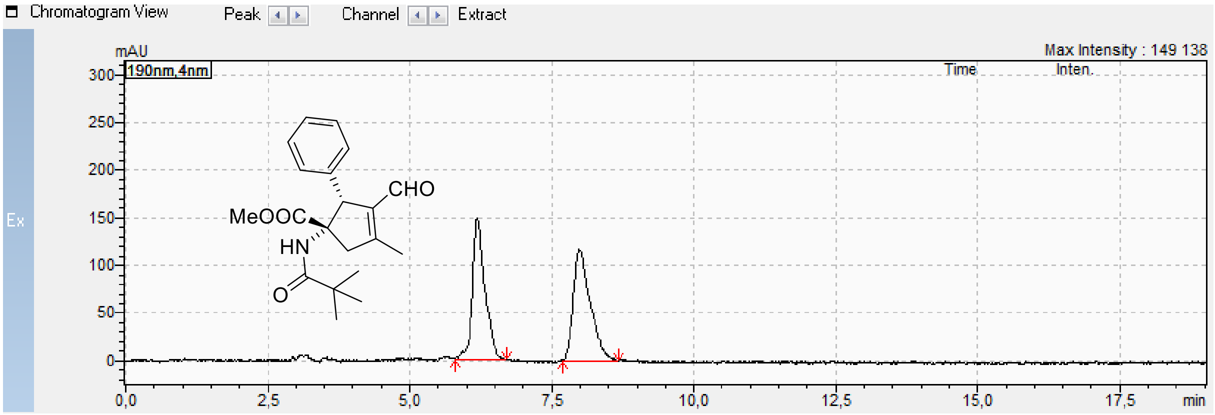

Results View - Peak Table

Peak Table | Compound | Group | Calibration Curve

Peak#	Ret. Time	Conc.	Area	Height	Similarity Index	Mark	Peak Start	Peak End	Area%
1	6,192	48,349	2274214	147913	0,000000	M	5,803	6,720	48,349
2	7,983	51,651	2429494	116483	0,000000	M	7,680	8,672	51,651

Chiral

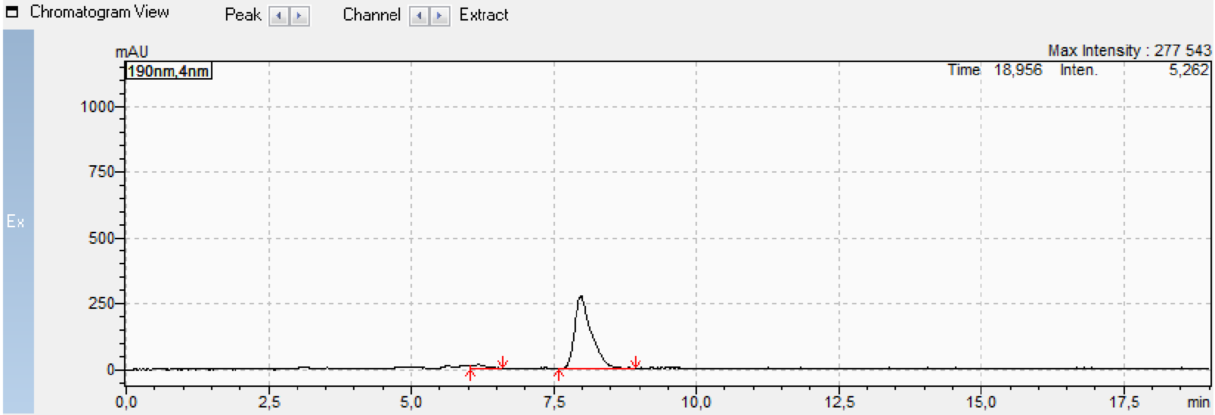

Results View - Peak Table

Peak Table | Compound | Group | Calibration Curve

Peak#	Ret. Time	Conc.	Area	Height	Similarity Index	Mark	Peak Start	Peak End	Area%
1	6,175	4,996	293085	17625	0,000000	M	6,037	6,592	4,996
2	7,984	95,004	5573895	273170	0,000000	M	7,595	8,939	95,004

5a

IA 70:30

Racemate

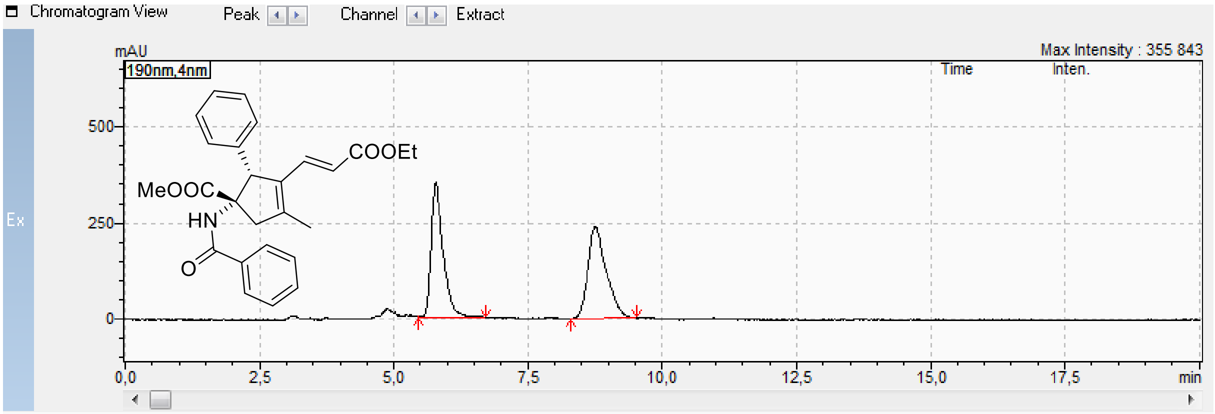

Results View - Peak Table

Peak Table | Compound | Group | Calibration Curve

Peak#	Ret. Time	Conc.	Area	Height	Similarity Index	Mark	Peak Start	Peak End	Area%
1	5,781	48,886	5411310	351025	0,000000	M	5,472	6,709	48,886
2	8,752	51,114	5657974	237391	0,000000	M	8,309	9,515	51,114

Chiral

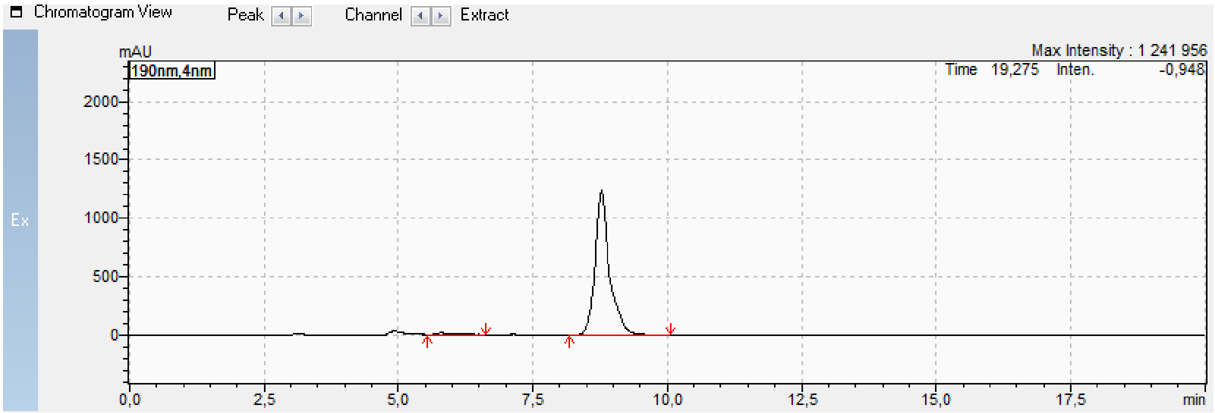

Results View - Peak Table

Peak Table | Compound | Group | Calibration Curve

Peak#	Ret. Time	Conc.	Area	Height	Similarity Index	Mark	Peak Start	Peak End	Area%
1	5,812	1,964	459195	18683	0,000000	M	5,536	6,624	1,964
2	8,771	98,036	22922825	1240926	0,000000	M	8,171	10,048	98,036

6a

IA 70:30

Racemate

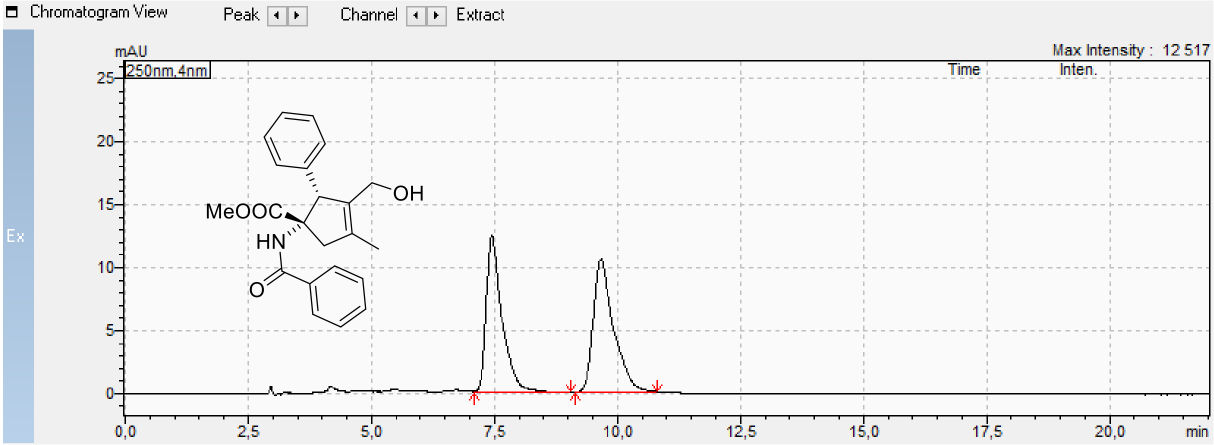

Results View - Peak Table

Peak Table | Compound | Group | Calibration Curve

Peak#	Ret. Time	Area	Height	Peak Start	Peak End	Mark	Conc.	Unit	Area%
1	7,449	279983	12420	7,083	9,056	M	48,105		48,105
2	9,666	302040	10541	9,152	10,805		51,895		51,895

Chiral

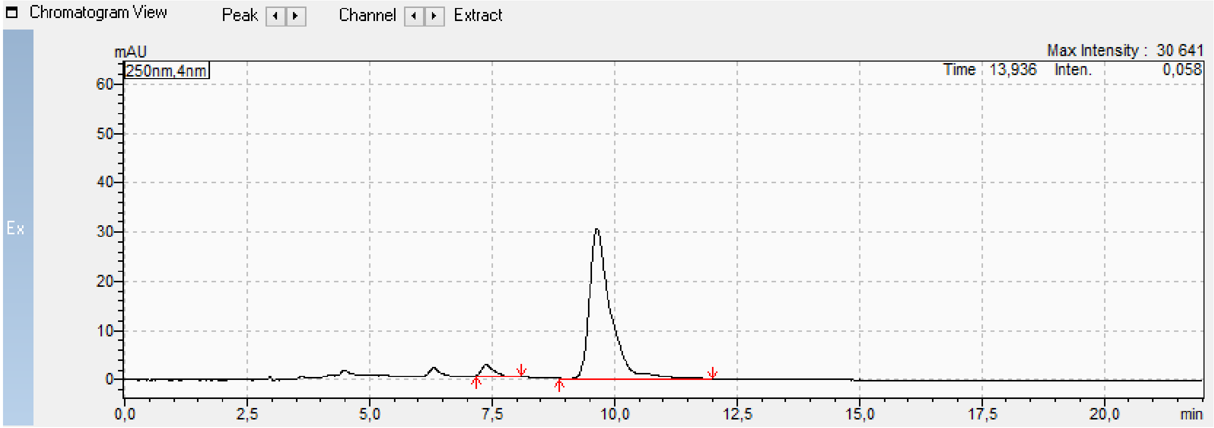

Results View - Peak Table

Peak Table | Compound | Group | Calibration Curve

Peak#	Ret. Time	Area	Height	Peak Start	Peak End	Mark	Conc.	Unit	Area%
1	7,384	39338	2316	7,168	8,096	M	4,169		4,169
2	9,645	904288	30387	8,875	12,000	M	95,831		95,831
